# Design, synthesis, *in silico* studies, and antiproliferative activity of a novel series of thiazole/1,2,3-triazole hybrids as apoptosis inducers and multi-kinase inhibitors endowed with anti-breast cancer activity

**DOI:** 10.1039/d5ra07556d

**Published:** 2026-01-15

**Authors:** Fatma A. M. Mohamed, Saleha Y. M. Alakilli, Hassan H. Alhassan, Sara Osman Yousif, Eid Alatwi, Hesham A. M. Gomaa, Heba Abu Alrub, Bandar A. Alyami, Ahmed H. Abdelhafez, Stefan Bräse, Bahaa G. M. Youssif

**Affiliations:** a Department of Clinical Laboratory Sciences, College of Applied Medical Sciences at Al-Qurayyat, Jouf University Al-Qurayyat 77454 Saudi Arabia fatmaahmed@ju.edu.sa; b Department of Biological Sciences, Faculty of Sciences, King Abdulaziz University Jeddah 23761 Saudi Arabia; c Department of Clinical Laboratories Sciences, College of Applied Medical Sciences, Jouf University Sakaka 72388 Saudi Arabia; d Department of Pharmacology, College of Pharmacy, Jouf University Sakaka 72388 Saudi Arabia; e Department of Pharmaceutical Chemistry, College of Pharmacy, Najran University Najran Saudi Arabia; f PharmaHelp Foundation Inc. Falls Church Virginia USA; g Institute of Biological and Chemical Systems, IBCS-FMS, Karlsruhe Institute of Technology 76131 Karlsruhe Germany braese@kit.edu; h Department of Pharmaceutical Organic Chemistry, Faculty of Pharmacy, Assiut University Assiut-71526 Egypt bgyoussif2@gmail.com +201044353895

## Abstract

A novel series of thiazole/1,2,3-triazole hybrids has been developed and evaluated for their *in vitro* anticancer efficacy. Compounds 10c, 10e, 10k, 10m, 10n, and 10o exhibited superior anticancer efficacy against the evaluated cancer cell lines, demonstrating a favorable safety profile, particularly against MCF-7 breast cancer, compared to erlotinib. The *in vitro* anti-breast cancer assay of compounds 10e and 10k demonstrated potent antiproliferative activity against the MCF-7 breast cancer cell line, with IC_50_ values of 24 nM and 21 nM, respectively, relative to the reference erlotinib, which exhibited an IC_50_ value of 40 nM. To elucidate their antiproliferative mechanism, tests for EGFR, HER-2, VEGFR-2, and BRAF^V600E^ kinases were performed. Compounds 10e and 10k exhibited the highest potency as multi-EGFR/HER-2/VEGFR-2 kinase inhibitors, with IC_50_ values of 73 ± 4 nM (EGFR), 31 ± 2 nM (HER-2), and 20 ± 1 nM (VEGFR-2), and 69 ± 4 nM (EGFR), 29 ± 1 nM (HER-2), and 21 ± 1 nM (VEGFR-2), respectively. The BRAF^V600E^ inhibitory testing results indicated weak to moderate effectiveness for the evaluated compounds. Findings from assays of apoptotic markers (Bax, Bcl2, and p53) indicate that apoptosis may contribute to the antiproliferative effects of the examined compounds. Analysis revealed that the 1,2,3-triazole moiety, the *para*-substituted methoxy group, and the chalcone moiety are essential variables in enhancing activity. The *in silico* docking studies against EGFR, HER-2, and VEGFR-2 revealed the importance of the phenyl 1,2,3-triazole moiety and the chalcone side chain in anticancer activity. The most potent compounds demonstrated drug-like properties and could serve as prototypes for future optimization. Compounds 10e and 10k may serve as examples of multi-targeting anticancer agents that function by blocking the EGFR, HER-2, and VEGFR-2 kinases.

## Abstract 2

This study reported the development of a novel series of thiazole/1,2,3-triazole hybrids and *in vitro* anticancer efficacy. Compounds 10c, 10e, 10k, 10m, 10n, and 10o exhibited superior anticancer efficacy, particularly against MCF-7 breast cancer.

## Graphical abstract

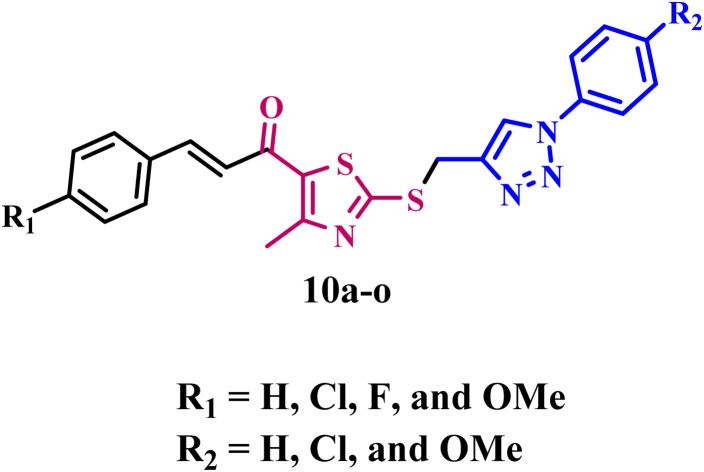

## 1. Introduction

Cancer is characterized as a group of disorders that infiltrate other regions of the body.^1^ Cancer is the second biggest cause of global mortality, following cardiac diseases, and constitutes a significant health burden.^2–4^ Despite the availability of numerous effective anticancer therapies, their side effects, such as drug resistance, a lack of differentiation between malignant and benign cells, limitations of radiotherapy, and the need for surgical intervention, highlight the need for effective alternatives that employ a variety of mechanisms to achieve success.^5–7^

Protein tyrosine kinases (PTKs) serve a key role in human cell signaling, directing cellular proliferation, differentiation, angiogenesis, and many regulatory processes.^8,9^ The overexpression of PTKs is essential in the genesis and progression of cancer due to their significant roles in cellular hemostasis.^10^ Moreover, PTK malfunction is fundamental to most cancer types, with PTKs constituting over 60% of all oncoproteins and proto-oncoproteins, which are pivotal in cancer pathology.^11^ Epidermal growth factor receptor (EGFR or ErbB1) and human epidermal growth factor receptor 2 (HER-2 or ErbB2) are two PTKs within the Erb-B family, which are overexpressed in a variety of solid tumors, including colon, breast, ovarian, lung, and prostate cancers.^12,13^ As a result, both receptors have been identified as plausible targets for cancer therapy.^14,15^ Vascular endothelial growth factor receptor-2 (VEGFR-2)^16^ is another crucial PTK that plays a role in angiogenesis, the process of producing new blood vessels, which is essential in various diseases, including cancer.^17^ VEGFR-2 is important to cancer progression and development, acting as the primary regulator of angiogenesis.^18^ The activation of VEGFR-2 by vascular endothelial growth factor (VEGF) is essential for the launch of tumor angiogenesis.^19^ Downstream signaling cascades and particular endothelial activities, such as enhanced permeability of vascular endothelial cells and augmented endothelial proliferation, ultimately result in angiogenesis.^20^ Inhibiting the VEGF/VEGFR-2 signaling network is thus an effective therapeutic strategy for suppressing tumor growth.^21^ In addition, inhibiting VEGFR-2 has been demonstrated to increase apoptosis in tumor cells, boosting the anticancer effect.^22,23^

Heterocycles, particularly azoles, play a crucial role in contemporary medicinal chemistry due to their numerous applications in drug design and discovery.^24,25^ Their potential use in various medical sectors, particularly as anticancer drugs, are being investigated. Thiazole, a five-membered heterocyclic moiety containing sulfur and nitrogen, garnered considerable attention due to its significant biological properties.^26,27^ Thiazole and its derivatives represent one of the most potent groups of chemicals, renowned for their extensive range of activities, with anticancer properties being paramount.^28,29^ Furthermore, thiazole-containing compounds have been found in a variety of clinically approved anticancer medications, including dasatinib (a tyrosine kinase inhibitor),^30^ dabrafenib (an inhibitor BRAF protein kinase),^31^ and patellamide A (cytotoxicity against multidrug-resistant malignancy).^32^

**Fig. 1:**
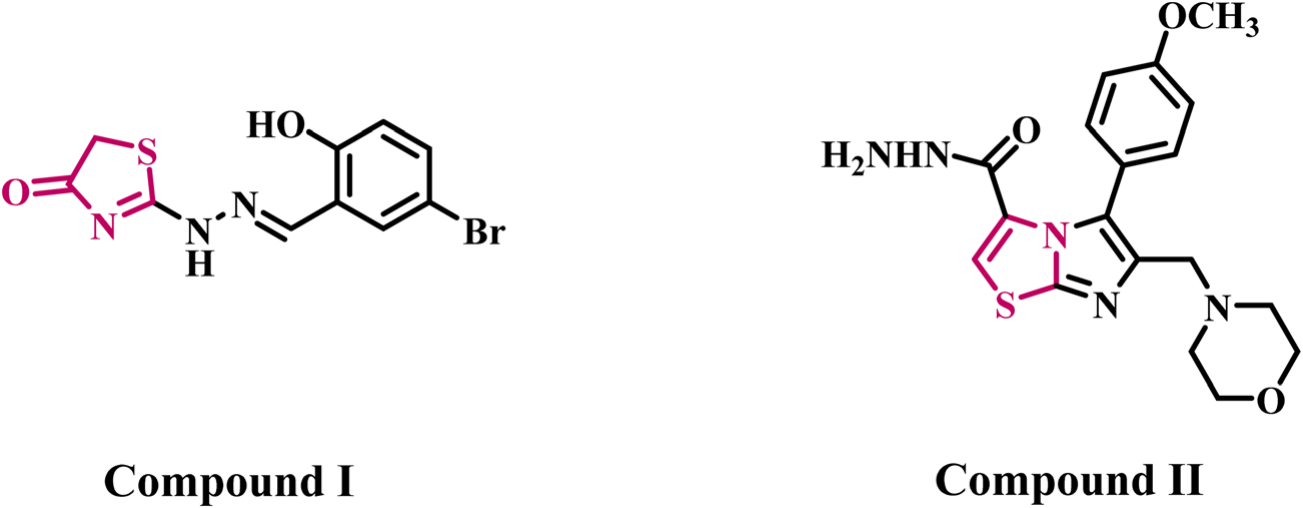
Structures of thiazole derivatives I and II as dual EGFR/HER-2 inhibitors.

Lv *et al.*^33^ presented two series of thiazole-derived compounds and evaluated their inhibitory effects on EGFR and HER-2 kinases. Various synthesized compounds exhibited significant inhibitory effects against EGFR and HER-2. Compound I (Fig. 1) exhibited the highest EGFR and HER-2 inhibitory activity (IC_50_ = 0.09 µM for EGFR and IC_50_ = 0.42 µM for HER-2) in breast cancer (MCF-7) cancer cell lines relative to erlotinib. The EGFR molecular docking model revealed that compound I had excellent binding to the hydroxyl group and established hydrogen bonds with Met 769 and Cys 751.

Sabry *et al.* reported a novel series of thiazole-based derivatives as potent anti-proliferative agents for the development of multi-targeting anticancer therapeutics. Compound II (Fig. 1) exhibited the highest antiproliferative efficacy against EGFR (IC_50_ = 0.122 µM) and HER-2 (IC_50_ = 0.078 µM) kinases, as well as MCF-7 breast cancer. Additionally, compound II was observed to induce cytotoxicity in MCF-7 breast cancer cells through apoptosis and cell cycle arrest at the G1/S phase. Compound II was determined to comply with Lipinski's rule of five and had a favorable ADMET profile. Finally, the molecular modeling investigations of compound II revealed a strong binding affinity for the Lys745 and Arg841 amino acid residues in the EGFR kinase active site, as well as Met801 in the HER-2 kinase active site.^34^

On the other hand, 1,2,3-triazoles are nitrogenous heterocycles characterized by the presence of three nitrogen atoms inside the ring structure. 1,2,3-Triazoles are stable entities that engage with biological targets through hydrogen bond formation, therefore serving as significant scaffolds in pharmaceutical development.^35–38^ 1,2,3-Triazoles have been demonstrated to possess anticancer properties through various mechanisms, including their function as tyrosine kinase inhibitors.^39^

**Fig. 2:**
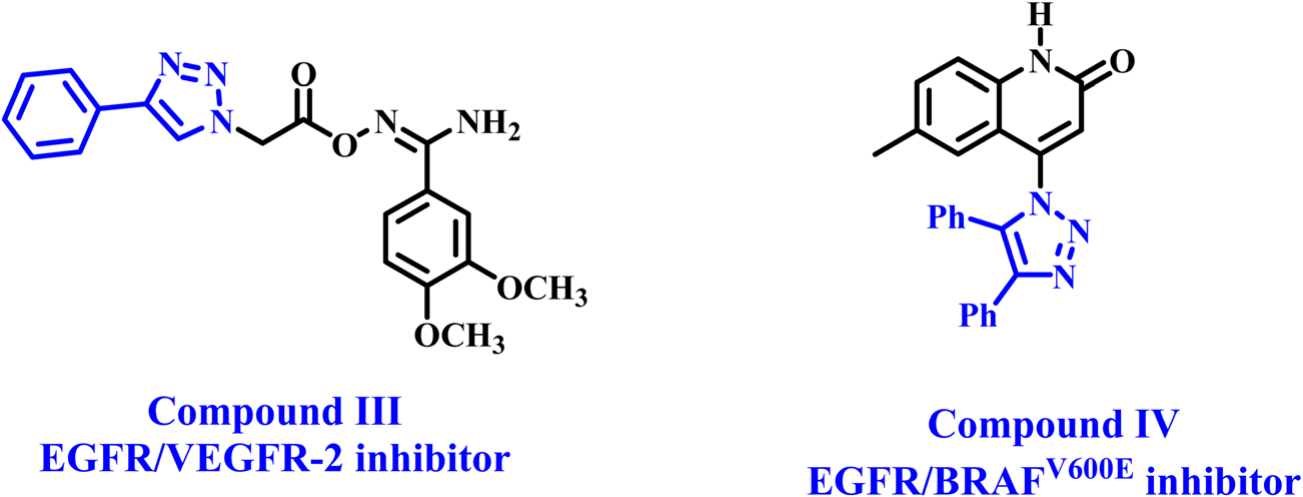
Structures of 1,2,3-triazole-based anticancer agents III and IV.

In a recent publication from our lab,^39^ we described the design and synthesis of a new series of 1,2,3-triazole-based aryl carboximidamide derivatives as antiproliferative agents with multi-targeting inhibitory action. The results revealed that compound III (Fig. 2) is the most potent EGFR/VEGFR-2 inhibitor, with IC_50_ values of 83 and 1.80 nM against EGFR and VEGFR-2, respectively. The apoptotic markers experiment indicated that compound III significantly elevated caspase-3 and Bax levels while reducing the anti-apoptotic Bcl-2 protein levels. The ADMET of compound III demonstrated safety and an excellent pharmacokinetic profile.

In another paper,^35^ we present the antiproliferative efficacy of new 1,2,3-triazole/quinoline hybrids as multi-target inhibitors of EGFR, BRAF^V600E^, and EGFR^T790M^. Compound IV (Fig. 2) exhibited the highest inhibitory potency against the examined molecular targets, with IC_50_ values of 57, 68, and 9.70 nM, respectively. The apoptotic assay results indicated that compound IV acts as an activator of caspase-3, caspase-8, and Bax, while also serving as a down-regulator of the antiapoptotic protein Bcl-2. Compound IV exhibited notable antioxidant activity at 10 µM, demonstrating DPPH radical scavenging of 73.5%, compared to Trolox's 77.6%.

**Fig. 3:**
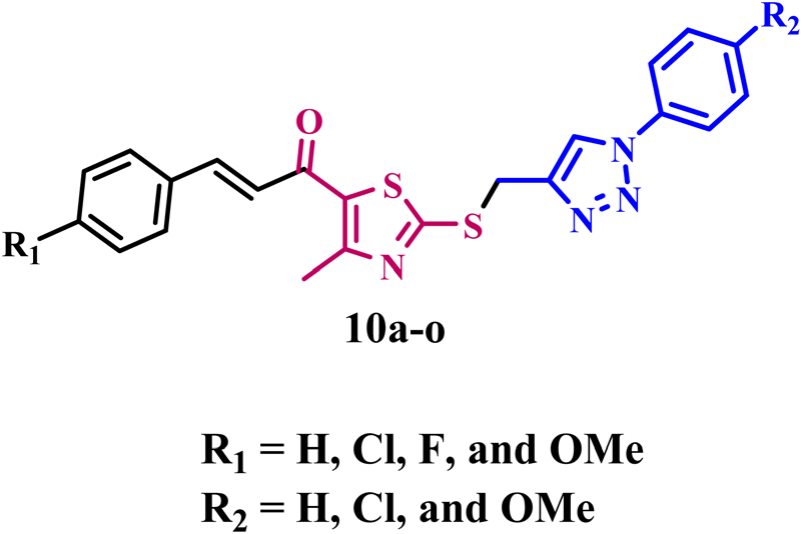
Structures of the new compounds 10a–o.

Inspired by prior data and as part of our ongoing efforts to identify a dual or multi-targeted antiproliferative agent,^35,40–45^ we present the design, synthesis, and antiproliferative efficacy of a novel series of thiazole/1,2,3-triazole hybrids (10a–o, Fig. 3) as multi-targeted inhibitors. The new compounds 10a–o are composed of thiazole and 1,2,3-triazole moieties, which, as indicated in the introduction, are known as antiproliferative agents that inhibit RTKs such as EGFR, HER-2, VEGFR-2, and the protein kinase BRAF^V600E^. The novel compounds also integrate the chalcone moiety, which is widely known for its anticancer effects, with the intention of increasing antiproliferative activity.^46,47^ The selection of various substituents (R_1_ and R_2_), whether as electron-withdrawing or electron-donating groups or atoms, to examine the influence of their electronic effects. All the newly synthesized compounds were validated by ^1^H NMR, ^13^C NMR, and elemental microanalysis.

The newly synthesized compounds were evaluated for their antiproliferative efficiency against a panel of four cancer cell lines. The most effective antiproliferative agents were subsequently evaluated as inhibitors of EGFR, BRAF^V600E^, HER-2, and VEGFR-2. Moreover, the apoptotic potential of the most potent derivatives against Bcl-2, p53, and Bax were examined. Finally, *in silico* studies were performed to investigate the binding interactions with the targeted receptors, as well as the pharmacokinetic properties of the new compounds.

## 2. Experimental

### 2.1. Chemistry

General details: refer to Appendix A (SI File).

#### 2.1.1. General procedure for the synthesis of compounds (6a–e)

A mixture of thiazole chalcones 5a–e (4 mmol), propargyl bromide (4 mmol; 476 mg), anhydrous sodium carbonate (6 mmol; 636 mg), and sodium iodide (8 mmol, 1.2 g) in acetone was stirred at room temperature for 6 h. The solvent was evaporated under reduced pressure, and the formed precipitate was thoroughly washed with a 10% sodium thiosulfate solution and distilled water. The product was then recrystallized from ethanol.

##### 2.1.1.1. (*E*)-1-(4-Methyl-2-(prop-2-yn-1-ylthio)thiazol-5-yl)-3-phenylprop-2-en-1-one (6a)

Yellow crystals; 0.983 g, 82% yield; mp 177–179 °C; ^1^H NMR (400 MHz, DMSO-*d*_6_) *δ* 7.81 (dd, *J* = 6.7, 2.9 Hz, 2H), 7.70 (d, *J* = 15.5 Hz, 1H), 7.47–7.46 (m, 3H), 7.39 (d, *J* = 15.5 Hz, 1H), 4.20 (d, *J* = 2.6 Hz, 2H), 3.35 (t, *J* = 2.6 Hz, 1H), 2.70 (s, 3H); ^13^C NMR (100 MHz, DMSO-*d*_6_) *δ* 181.98, 167.99, 158.55, 144.26, 134.62, 132.81, 131.39, 129.50, 129.32, 124.80, 79.27, 75.46, 22.03, 18.77; anal. calcd. For C_16_H_13_NOS_2_: C, 64.19%; H, 4.38%; N, 4.68%. Found: C, 64.29%; H, 4.32%; N, 4.75%.

##### 2.1.1.2. (*E*)-3-(4-Chlorophenyl)-1-(4-methyl-2-(prop-2-yn-1-ylthio)thiazol-5-yl)prop-2-en-1-one (6b)

Yellow crystals; 1.042 g, 78% yield; mp 183–184 °C; ^1^H NMR (400 MHz, DMSO-*d*_6_) *δ* 7.85 (d, *J* = 8.5 Hz, 2H), 7.68 (d, *J* = 15.6 Hz, 1H), 7.52 (d, *J* = 8.5 Hz, 2H), 7.41 (d, *J* = 15.5 Hz, 1H), 4.20 (d, *J* = 2.6 Hz, 2H), 3.35 (t, *J* = 2.6 Hz, 1H), 2.70 (s, 3H); ^13^C NMR (100 MHz, DMSO-*d*_6_) *δ* 181.82, 168.12, 158.72, 142.77, 135.87, 133.58, 132.70, 131.02, 129.52, 125.48, 79.25, 75.45, 22.04, 18.77; anal. calcd. For C_16_H_12_ClNOS_2_: C, 57.56%; H, 3.62%; N, 4.20%. Found: C, 57.71%; H, 3.55%; N, 4.18%

##### 2.1.1.3. (*E*)-3-(4-Fluorophenyl)-1-(4-methyl-2-(prop-2-yn-1-ylthio)thiazol-5-yl)prop-2-en-1-one (6c)

Yellow crystals; 1.016 g, 80% yield; mp 180–182 °C; ^1^H NMR (400 MHz, DMSO-*d*_6_) *δ* 7.90 (dd, *J* = 8.7, 5.6 Hz, 2H), 7.70 (d, *J* = 15.5 Hz, 1H), 7.37–7.28 (m, 3H), 4.19 (d, *J* = 2.6 Hz, 2H), 3.35 (t, *J* = 2.6 Hz, 1H), 2.69 (s, 3H); ^13^C NMR (100 MHz, DMSO-*d*_6_) *δ* 181.42, 167.51, 164.80, 162.32, 158.10, 142.59, 132.29, 131.24, 124.18, 116.16, 78.79, 74.97, 21.55, 18.28; anal. calcd. For C_16_H_12_FNOS_2_: C, 60.55%; H, 3.81%; N, 4.41%. Found: C, 60.68%; H, 3.70%; N, 4.52%.

##### 2.1.1.4. (*E*)-1-(4-Methyl-2-(prop-2-yn-1-ylthio)thiazol-5-yl)-3-(*p*-tolyl)prop-2-en-1-one (6d)

Yellow crystals; 1.066 g, 85% yield; mp 187–188 °C; ^1^H NMR (400 MHz, DMSO-*d*_6_) *δ* 7.72–7.66 (m, 3H), 7.34 (d, *J* = 15.5 Hz, 1H), 7.28 (d, *J* = 7.7 Hz, 2H), 4.20 (d, *J* = 2.0 Hz, 2H), 3.36 (s, 1H), 2.70 (s, 3H), 2.36 (s, 3H); ^13^C NMR (100 MHz, DMSO-*d*_6_) *δ* 181.86, 167.55, 162.15, 158.13, 143.53, 134.62, 131.38, 128.67, 122.17, 115.32, 79.36, 75.34, 21.95, 21.58, 18.78; anal. calcd. For C_17_H_15_NOS_2_: C, 65.15%; H, 4.82%; N, 4.47%. Found: C, 65.25%; H, 4.75%; N, 4.52%.

##### 2.1.1.5. (*E*)-3-(4-Methoxyphenyl)-1-(4-methyl-2-(prop-2-yn-1-ylthio)thiazol-5-yl)prop-2-en-1-one (6e)

Brown crystals; 1.068 g, 81% yield; mp 192–194 °C; ^1^H NMR (400 MHz, DMSO-*d*_6_) *δ* 7.77 (d, *J* = 8.6 Hz, 2H), 7.66 (d, *J* = 15.4 Hz, 1H), 7.23 (d, *J* = 15.4 Hz, 1H), 7.01 (d, *J* = 8.6 Hz, 2H), 4.18 (d, *J* = 2.4 Hz, 2H), 3.82 (s, 3H), 2.68 (s, 3H); ^13^C NMR (100 MHz, DMSO-*d*_6_) *δ* 181.83, 167.53, 162.10, 158.19, 144.38, 132.93, 131.30, 127.15, 122.14, 115.00, 79.30, 75.32, 55.87, 21.99, 18.66; anal. calcd. For C_17_H_15_NO_2_S_2_: C, 61.98%; H, 4.59%; N, 4.25%. Found: C, 61.87%; H, 4.68%; N, 4.16%.

#### 2.1.2. General procedure for the synthesis of compounds (9a–c)^48^

Aniline derivatives 7a–c (1 mmol) were dissolved in 15 mL of distilled water containing 10 mL of concentrated HCl and cooled in an ice bath. Then a solution of sodium nitrite (1.3 mmol, 90 mg) in water was added dropwise over 15 min, and the mixture was stirred for an additional 30 min at 0 °C to complete diazotization. Subsequently, a solution of sodium azide (1.3 mmol, 85 mg) in water was added dropwise over 15 min, and the reaction mixture was stirred for 2 h at 0 °C. The formed azides were extracted with methylene chloride (3 × 20 mL), and the combined organic layers were dried over anhydrous sodium sulfate, filtered, and concentrated under reduced pressure to afford the corresponding azides 9a–c as yellow oils which were used directly in the next step without further purification. The procedure was adapted from reported methods.^48^

#### 2.1.3. General procedure for the synthesis of compounds (10a–o)

Compounds 6a–e (1 mmol) and aromatic azides 9a–c (1.1 mmol) were dissolved in a 1 : 1 THF water mixture (20 mL). Then, sodium (l)-ascorbate (99 mg, 0.5 mmol) and copper sulfate (25 mg, 0.1 mmol) were added portion-wise. The reaction mixture was stirred at room temperature for 5 h. The formed precipitate was filtered off, washed with water, and then recrystallized from absolute ethanol.

##### 2.1.3.1. (*E*)-1-(4-Methyl-2-(((1-phenyl-1*H*-1,2,3-triazol-4-yl)methyl)thio)thiazol-5-yl)-3-phenylprop-2-en-1-one (10a)

Yellow powder; 0.298 g, 71% yield; mp 223–224 °C; ^1^H NMR (400 MHz, DMSO-*d*_6_) *δ* 8.84 (s, 1H), 7.90 (d, *J* = 8.0 Hz, 2H), 7.81–7.80 (m, 2H), 7.69 (d, *J* = 15.5 Hz, 1H), 7.60 (t, *J* = 7.8 Hz, 2H), 7.51 (d, *J* = 7.4 Hz, 1H), 7.48–7.46 (m, 3H), 7.38 (d, *J* = 15.5 Hz, 1H), 4.73 (s, 2H), 2.72 (s, 3H); ^13^C NMR (100 MHz, DMSO-*d*_6_) *δ* 181.92, 168.56, 158.67, 144.16, 143.69, 136.95, 134.61, 132.37, 131.38, 130.38, 129.49, 129.30, 129.26, 124.89, 122.61, 120.60, 28.33, 18.77; anal. calcd. For C_22_H_18_N_4_OS_2_: C, 63.14%; H, 4.34%; N, 13.39%. Found: C, 63.30%; H, 4.43%; N, 13.27%.

##### 2.1.3.2. (*E*)-3-(4-Chlorophenyl)-1-(4-methyl-2-(((1-phenyl-1*H*-1,2,3-triazol-4-yl)methyl)thio)thiazol-5-yl)prop-2-en-1-one (10b)

Yellow powder; 0.340 g, 75% yield; mp 221–223 °C; ^1^H NMR (400 MHz, DMSO-*d*_6_) *δ* 8.83 (s, 1H), 7.88 (d, *J* = 7.7 Hz, 2H), 7.84 (d, *J* = 8.5 Hz, 2H), 7.67 (d, *J* = 15.5 Hz, 1H), 7.59 (t, *J* = 7.8 Hz, 2H), 7.52–7.47 (m, 3H), 7.39 (d, *J* = 15.5 Hz, 1H), 4.72 (s, 2H), 2.71 (s, 3H); ^13^C NMR (100 MHz, DMSO-*d*_6_) *δ* 181.91, 168.60, 159.70, 158.65, 144.18, 134.60, 133.50, 132.36, 131.38, 130.36, 129.51, 129.32, 125.00, 122.71, 122.34, 115.30, 28.34, 18.74; anal. calcd. For C_22_H_17_ClN_4_OS_2_: C, 58.33%; H, 3.78%; N, 12.37%. Found: C, 58.45%; H, 3.68%; N, 12.42%.

##### 2.1.3.3. (*E*)-3-(4-Fluorophenyl)-1-(4-methyl-2-(((1-phenyl-1*H*-1,2,3-triazol-4-yl)methyl)thio)thiazol-5-yl)prop-2-en-1-one (10c)

Yellow powder; 0.363 g, 83% yield; mp 236–238 °C; ^1^H NMR (400 MHz, DMSO-*d*_6_) *δ* 8.83 (s, 1H), 7.89–7.88 (m, 4H), 7.68 (d, *J* = 15.5 Hz, 1H), 7.59 (t, *J* = 7.7 Hz, 2H), 7.48 (t, *J* = 7.3 Hz, 1H), 7.35–7.27 (m, 3H), 4.72 (s, 2H), 2.71 (s, 3H); ^13^C NMR (100 MHz, DMSO-*d*_6_) *δ* 181.93, 168.63, 159.78, 158.55, 143.40, 140.35, 134.73, 132.30, 131.72, 131.62, 131.36, 125.04, 123.58, 122.32, 116.33, 114.95, 28.31, 18.74; anal. calcd. For C_22_H_17_FN_4_OS_2_: C, 60.53%; H, 3.93%; N, 12.84%. Found: C, 60.61%; H, 4.03%; N, 12.70%.

##### 2.1.3.4. (*E*)-1-(4-Methyl-2-(((1-phenyl-1*H*-1,2,3-triazol-4-yl)methyl)thio)thiazol-5-yl)-3-(*p*-tolyl)prop-2-en-1-one (10d)

Yellow powder; 0.363 g, 74% yield; mp 230–233 °C; ^1^H NMR (400 MHz, DMSO-*d*_6_) *δ* 8.84 (s, 1H), 7.90 (d, *J* = 7.9 Hz, 2H), 7.70–7.64 (m, 3H), 7.60 (t, *J* = 7.7 Hz, 2H), 7.50 (t, *J* = 7.4 Hz, 1H), 7.32 (d, *J* = 15.5 Hz, 1H), 7.27 (d, *J* = 7.9 Hz, 2H), 4.73 (s, 2H), 2.71 (s, 3H), 2.35 (s, 3H); ^13^C NMR (100 MHz, DMSO-*d*_6_) *δ* 181.77, 167.99, 162.04, 158.41, 144.35, 143.00, 137.05, 132.62, 132.37, 130.31, 127.01, 124.21, 122.50, 120.96, 114.90, 113.98, 28.28, 21.56, 18.74; anal. calcd. For C_23_H_20_N_4_OS_2_: C, 63.86%; H, 4.66%; N, 12.95%. Found: C, 64.02%; H, 4.75%; N, 13.08%.

##### 2.1.3.5. (*E*)-3-(4-Methoxyphenyl)-1-(4-methyl-2-(((1-phenyl-1*H*-1,2,3-triazol-4-yl)methyl)thio)thiazol-5-yl)prop-2-en-1-one (10e)

Yellow powder; 0.323 g, 72% yield; mp 242–244 °C; ^1^H NMR (400 MHz, DMSO-*d*_6_) *δ* 8.84 (s, 1H), 7.90 (d, *J* = 7.8 Hz, 2H), 7.77 (d, *J* = 8.6 Hz, 1H), 7.66 (d, *J* = 15.4 Hz, 1H), 7.60 (t, *J* = 7.7 Hz, 2H), 7.50 (t, *J* = 7.3 Hz, 1H), 7.24 (d, *J* = 15.4 Hz, 1H), 7.02 (d, *J* = 8.6 Hz, 2H), 4.73 (s, 2H), 3.83 (s, 3H), 2.71 (s, 3H); ^13^C NMR (100 MHz, DMSO-*d*_6_) *δ* 181.80, 168.05, 162.09, 158.23, 144.28, 143.74, 136.96, 132.56, 131.30, 130.39, 129.26, 127.22, 122.61, 122.32, 120.60, 115.00, 55.90, 28.33, 18.71; anal. calcd. For C_23_H_20_N_4_O_2_S_2_: C, 61.59%; H, 4.49%; N, 12.49%. Found: C, 61.63%; H, 4.57%; N, 12.70%.

##### 2.1.3.6. (*E*)-1-(2-(((1-(4-Chlorophenyl)-1*H*-1,2,3-triazol-4-yl)methyl)thio)-4-methylthiazol-5-yl)-3-phenylprop-2-en-1-one (10f)

Yellow powder; 0.318 g, 70% yield; mp 231–232 °C; ^1^H NMR (400 MHz, DMSO-*d*_6_) *δ* 8.85 (s, 1H), 7.94 (d, *J* = 8.8 Hz, 2H), 7.80 (dd, *J* = 6.3, 2.7 Hz, 2H), 7.70–7.65 (m, 3H), 7.47–7.46 (m, 3H), 7.38 (d, *J* = 15.5 Hz, 1H), 4.72 (s, 2H), 2.71 (s, 3H); ^13^C NMR (100 MHz, DMSO-*d*_6_) *δ* 181.96, 168.52, 158.67, 144.19, 143.94, 135.75, 134.61, 133.53, 132.40, 131.40, 130.35, 129.51, 129.31, 124.90, 122.72, 122.32, 28.26, 18.77; anal. calcd. For C_22_H_17_ClN_4_OS_2_: C, 58.33%; H, 3.78%; N, 12.37%. Found: C, 58.17%; H, 3.66%; N, 12.49%.

##### 2.1.3.7. (*E*)-3-(4-Chlorophenyl)-1-(2-(((1-(4-chlorophenyl)-1*H*-1,2,3-triazol-4-yl)methyl)thio)-4-methylthiazol-5-yl)prop-2-en-1-one (10g)

Yellow powder; 0.322 g, 66% yield; mp 233–236 °C; ^1^H NMR (400 MHz, DMSO-*d*_6_) *δ* 8.86 (s, 1H), 7.94 (d, *J* = 6.8 Hz, 2H), 7.85 (d, *J* = 5.9 Hz, 2H), 7.68–7.66 (m, 3H), 7.53 (d, *J* = 3.8 Hz, 2H), 7.40 (d, *J* = 14.3 Hz, 1H), 4.73 (s, 2H), 2.72 (s, 3H); ^13^C NMR (100 MHz, DMSO-*d*_6_) *δ* 181.89, 168.44, 159.79, 144.15, 143.29, 135.40, 133.01, 132.45, 131.39, 130.38, 126.31, 122.29, 121.23, 117.26, 116.05, 114.24, 28.33, 18.72; anal. calcd. For C_22_H_16_Cl_2_N_4_OS_2_: C, 54.21%; H, 3.31%; N, 11.49%. Found: C, 54.35%; H, 3.24%; N, 11.36%.

##### 2.1.3.8. (*E*)-1-(2-(((1-(4-Chlorophenyl)-1*H*-1,2,3-triazol-4-yl)methyl)thio)-4-methylthiazol-5-yl)-3-(4-fluorophenyl)prop-2-en-1-one (10h)

Yellow powder; 0.368 g, 78% yield; mp 243–245 °C; ^1^H NMR (400 MHz, DMSO-*d*_6_) *δ* 8.86 (s, 1H), 7.95–7.90 (m, 4H), 7.70–7.68 (m, 3H), 7.37–7.31 (m, 3H), 4.73 (s, 2H), 2.72 (s, 3H); ^13^C NMR (100 MHz, DMSO-*d*_6_) *δ* 181.89, 168.60, 160.33, 158.42, 143.04, 133.09, 132.33, 131.68, 131.30, 130.42, 126.22, 122.58, 121.29, 116.73, 116.17, 114.89, 28.37, 18.78; anal. calcd. For C_22_H_16_ClFN_4_OS_2_: C, 56.11%; H, 3.42%; N, 11.90%. Found: C, 56.22%; H, 3.50%; N, 11.99%.

##### 2.1.3.9. (*E*)-1-(2-(((1-(4-Chlorophenyl)-1*H*-1,2,3-triazol-4-yl)methyl)thio)-4-methylthiazol-5-yl)-3-(*p*-tolyl)prop-2-en-1-one (10i)

Yellow powder; 0.323 g, 69% yield; mp 234–237 °C; ^1^H NMR (400 MHz, DMSO-*d*_6_) *δ* 8.87 (s, 1H), 7.96–7.93 (m, 2H), 7.69–7.68 (m, 5H), 7.33–7.28 (m, 3H), 4.73 (s, 2H), 2.71 (s, 3H), 2.36 (s, 3H); ^13^C NMR (100 MHz, DMSO-*d*_6_) *δ* 181.80, 168.47, 160.86, 158.31, 144.22, 143.61, 136.93, 134.59, 131.58, 131.20, 130.81, 129.41, 124.62, 122.38, 116.47, 114.26, 28.36, 21.55, 18.78; anal. calcd. For C_23_H_19_ClN_4_OS_2_: C, 59.15%; H, 4.10%; N, 12.00%. Found: C, 59.07%; H, 4.16%; N, 12.10%.

##### 2.1.3.10. (*E*)-1-(2-(((1-(4-Chlorophenyl)-1*H*-1,2,3-triazol-4-yl)methyl)thio)-4-methylthiazol-5-yl)-3-(4-methoxyphenyl)prop-2-en-1-one (10j)

Yellow powder; 0.353 g, 73% yield; mp 222–224 °C; ^1^H NMR (400 MHz, DMSO-*d*_6_) *δ* 8.86 (s, 1H), 7.95–7.94 (m, 2H), 7.77–7.76 (m, 2H), 7.67–7.66 (m, 3H), 7.23 (d, *J* = 14.7 Hz, 2H), 7.02–7.01 (m, 2H), 4.72 (s, 2H), 3.82 (s, 3H), 2.71 (s, 3H); ^13^C NMR (100 MHz, DMSO-*d*_6_) *δ* 181.78, 167.98, 162.09, 158.22, 144.27, 143.96, 135.75, 133.51, 132.57, 131.29, 130.33, 127.21, 122.69, 122.29, 114.99, 114.23, 55.89, 28.26, 18.71; anal. calcd. For C_23_H_19_ClN_4_O_2_S_2_: C, 57.20%; H, 3.97%; N, 11.60%. Found: C, 57.27%; H, 4.11%; N, 11.69%.

##### 2.1.3.11. (*E*)-1-(2-(((1-(4-Methoxyphenyl)-1*H*-1,2,3-triazol-4-yl)methyl)thio)-4-methylthiazol-5-yl)-3-phenylprop-2-en-1-one (10k)

Yellow powder; 0.301 g, 67% yield; mp 220–221 °C; ^1^H NMR (400 MHz, DMSO-*d*_6_) *δ* 8.73 (s, 1H), 7.81–7.79 (m, 4H), 7.69 (d, *J* = 15.5 Hz, 1H), 7.48 (s, 3H), 7.39 (d, *J* = 15.6 Hz, 1H), 7.13 (d, *J* = 8.3 Hz, 2H), 4.71 (s, 2H), 3.83 (s, 3H), 2.72 (s, 3H); ^13^C NMR (100 MHz, DMSO-*d*_6_) *δ* 181.93, 168.62, 159.80, 158.67, 144.17, 134.62, 132.36, 131.47, 131.38, 130.38, 129.50, 129.31, 124.91, 122.59, 122.28, 115.35, 56.04, 28.38, 18.77; anal. calcd. For C_23_H_20_N_4_O_2_S_2_: C, 61.59%; H, 4.49%; N, 12.49%. Found: C, 61.43%; H, 4.58%; N, 12.35%.

##### 2.1.3.12. (*E*)-3-(4-Chlorophenyl)-1-(2-(((1-(4-methoxyphenyl)-1*H*-1,2,3-triazol-4-yl)methyl)thio)-4-methylthiazol-5-yl)prop-2-en-1-one (10l)

Yellow powder; 0.411 g, 85% yield; mp 241–243 °C; ^1^H NMR (400 MHz, DMSO-*d*_6_) *δ* 8.71 (s, 1H), 7.84 (d, *J* = 8.4 Hz, 2H), 7.78 (d, *J* = 9.0 Hz, 2H), 7.66 (d, *J* = 15.5 Hz, 1H), 7.52 (d, *J* = 8.4 Hz, 2H), 7.39 (d, *J* = 15.5 Hz, 1H), 7.12 (d, *J* = 9.0 Hz, 2H), 4.70 (s, 2H), 3.82 (s, 3H), 2.70 (s, 3H); ^13^C NMR (100 MHz, DMSO-*d*_6_) *δ* 181.84, 168.10, 159.79, 158.24, 143.92, 139.81, 136.96, 135.48, 131.45, 130.38, 127.23, 125.05, 122.71, 122.43, 116.77, 114.35, 56.05, 28.37, 18.75; anal. calcd. For C_23_H_19_ClN_4_O_2_S_2_: C, 57.20%; H, 3.97%; N, 11.60%. Found: C, 57.12%; H, 4.07%; N, 11.47%.

##### 2.1.3.13. (*E*)-3-(4-Fluorophenyl)-1-(2-(((1-(4-methoxyphenyl)-1*H*-1,2,3-triazol-4-yl)methyl)thio)-4-methylthiazol-5-yl)prop-2-en-1-one (10m)

Yellow powder; 0.378 g, 81% yield; mp 228–230 °C; ^1^H NMR (400 MHz, DMSO-*d*_6_) *δ* 8.72 (s, 1H), 7.89 (dd, *J* = 8.2, 5.8 Hz, 2H), 7.79 (d, *J* = 8.9 Hz, 2H), 7.69 (d, *J* = 15.5 Hz, 1H), 7.36–7.28 (m, 3H), 7.12 (d, *J* = 8.9 Hz, 2H), 4.70 (s, 2H), 3.82 (s, 3H), 2.71 (s, 3H); ^13^C NMR (100 MHz, DMSO-*d*_6_) *δ* 181.87, 168.64, 159.80, 158.69, 142.97, 132.32, 131.78, 131.69, 131.32, 130.36, 124.79, 122.58, 122.28, 116.64, 116.42, 115.36, 56.04, 28.37, 18.76; anal. calcd. For C_23_H_19_FN_4_O_2_S_2_: C, 59.21%; H, 4.11%; N, 12.01%. Found: C, 59.39%; H, 4.02%; N, 12.18%.

##### 2.1.3.14. (*E*)-1-(2-(((1-(4-Methoxyphenyl)-1*H*-1,2,3-triazol-4-yl)methyl)thio)-4-methylthiazol-5-yl)-3-(*p*-tolyl)prop-2-en-1-one (10n)

Yellow powder: 0.357 g, 77% yield; mp 235–236 °C; ^1^H NMR (400 MHz, DMSO-*d*_6_) *δ* 8.73 (s, 1H), 7.90 (d, *J* = 8.4 Hz, 2H), 7.78 (d, *J* = 9.0 Hz, 2H), 7.65 (d, *J* = 15.5 Hz, 1H), 7.34 (d, *J* = 15.5 Hz, 1H), 7.27 (d, *J* = 9.0 Hz, 2H), 7.12 (d, *J* = 8.4 Hz, 2H), 4.72 (s, 2H), 3.82 (s, 3H), 2.71 (s, 3H), 2.36 (s, 3H); *δ*; ^13^C NMR (100 MHz, DMSO-*d*_6_) *δ* 181.78, 168.55, 161.12, 158.70, 144.34, 143.73, 137.80, 134.61, 131.47, 131.22, 130.31, 129.50, 123.59, 120.17, 116.39, 113.81, 56.05, 28.32, 21.54, 18.77; anal. calcd. For C_24_H_22_N_4_O_2_S_2_: C, 62.32%; H, 4.79%; N, 12.11%. Found: C, 62.25%; H, 4.93%; N, 12.20%.

##### 2.1.3.15. (*E*)-3-(4-Methoxyphenyl)-1-(2-(((1-(4-methoxyphenyl)-1*H*-1,2,3-triazol-4-yl)methyl)thio)-4-methylthiazol-5-yl)prop-2-en-1-one (10o)

Yellow powder; 0.393 g, 82% yield; mp 227–228 °C; ^1^H NMR (400 MHz, DMSO-*d*_6_) *δ* 8.72 (s, 1H), 7.78 (t, *J* = 9.0 Hz, 4H), 7.66 (d, *J* = 15.4 Hz, 1H), 7.23 (d, *J* = 15.4 Hz, 1H), 7.12 (d, *J* = 8.8 Hz, 2H), 7.01 (d, *J* = 8.4 Hz, 2H), 4.70 (s, 2H), 3.82 (s, 6H), 2.70 (s, 3H); ^13^C NMR (100 MHz, DMSO-*d*_6_) *δ* 181.89, 168.29, 161.17, 158.60, 144.19, 143.06, 137.01, 132.54, 131.30, 130.32, 129.56, 124.67, 122.29, 120.34, 116.24, 113.19, 56.04, 55.93, 28.32, 18.79; anal. calcd. For C_24_H_22_N_4_O_3_S_2_: C, 60.23%; H, 4.63%; N, 11.71%. Found: C, 60.15%; H, 4.75%; N, 11.60%.

### 2.2. Biology

#### 2.2.1. Cell viability assay

The human mammary (MCF-10A) gland epithelial normal cell line was used to assess the effects of compounds 10a–o on cell viability. Following a 4-day incubation period on MCF-10A cells, the cell viability of 10a–o was assessed using the MTT test.^49,50^ For further information, see Appendix A.

#### 2.2.2. Antiproliferative assay

The antiproliferative effect of 10a–o was assessed using the MTT assay on four human cancer cell lines: MCF-7 for breast cancer, A-549 for lung cancer, Panc-1 for pancreatic cancer, and HT-29 for colon cancer. All cell lines were obtained from ATCC (American Type Cell Culture). Erlotinib served as a ref. 51 and 52. Using dose–response tests, the IC_50_ values for novel compounds were determined. Three replicates of each concentration were used in at least two independent experiments, yielding the reported values. Appendix A (SI File) contains experimental details.

#### 2.2.3. EGFR inhibitory assay

The EGFR-TK assay was employed to evaluate the inhibitory effectiveness of the most potent derivatives, 10c, 10e, 10k, 10m, 10n and 10o, against EGFR, using erlotinib as the reference compound.^53,54^ Appendix A (SI File) contains experimental details.

#### 2.2.4. HER-2 inhibitory assay

Using the kinase assay,^45^ the compounds 10c, 10e, 10k, 10m, 10n, and 10o were evaluated for their capacity to inhibit HER-2. Lapatinib was the reference substance. Refer to Appendix A for further information.

#### 2.2.5. BRAF^V600E^ inhibitory assay

An *in vitro* investigation evaluated the efficacy of derivatives 10c, 10e, and 10k as inhibitors of BRAF^V600E^. Vemurafenib served as the reference medication.^55^ Refer to Appendix A for additional experimental details.

#### 2.2.6. VEGFR-2 inhibitory assay

The inhibitory effects of compounds 10c, 10e, and 10k on VEGFR-2 were evaluated by kinase assays, with Sorafenib as the control drug.^21^ Refer to Appendix A for additional experimental details.

#### 2.2.7. Apoptotic markers assays

Compounds 10e and 10k were assessed for their capacity to induce apoptosis in MCF-7 breast cancer cells by examining the expression of crucial apoptotic markers, namely Bcl-2, p53, and Bax.^56^ For details of all experimental studies, see Appendix A (SI File).

### 2.3. *In silico* studies

For details of all *in silico* studies, see Appendix A (SI File).

## 3. Results and discussion

### 3.1. Chemistry

**Scheme 1:**
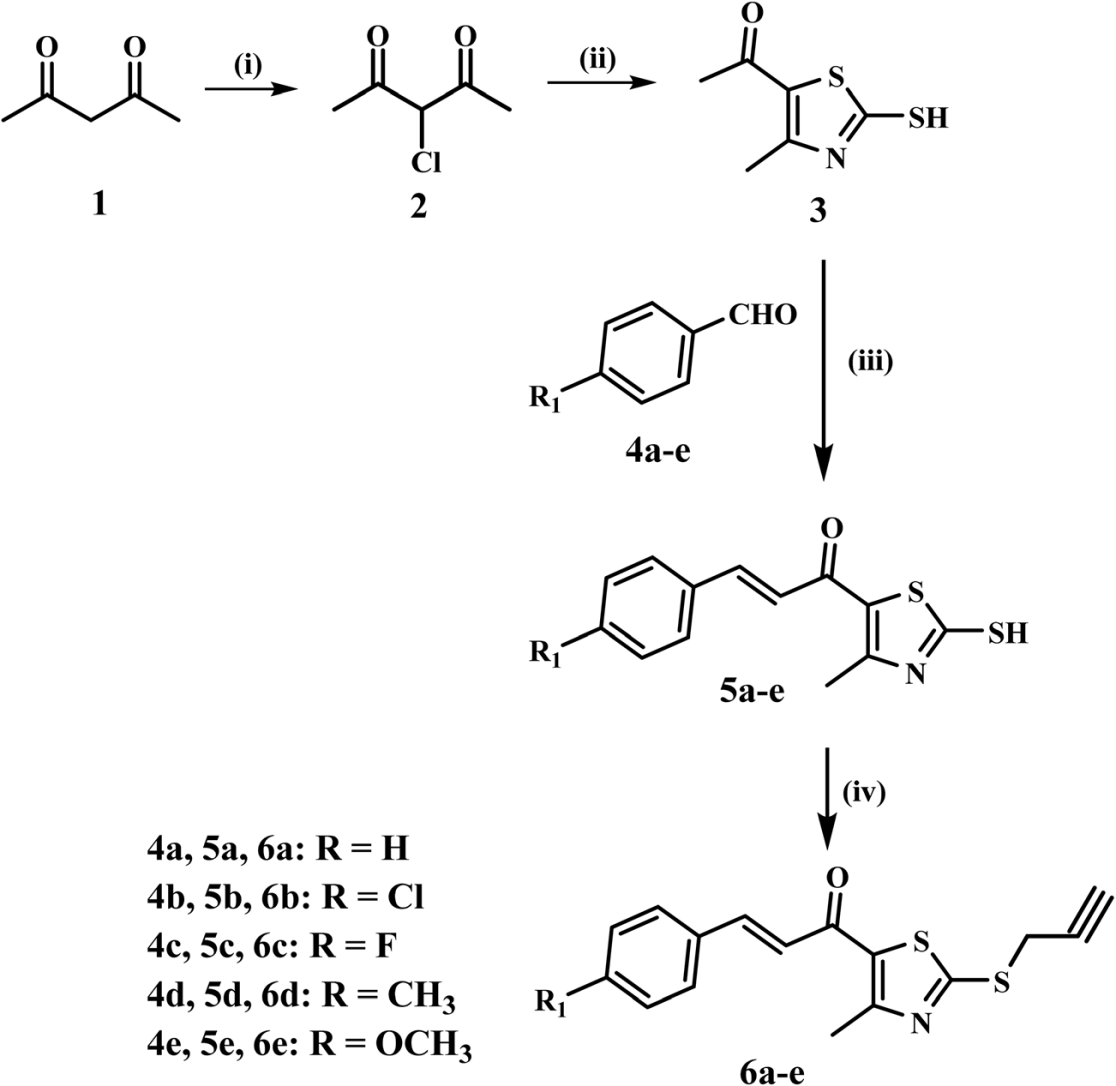
Synthesis of key intermediates 6a–e. Reagents and conditions: (i) SO_2_Cl_2_, toluene, 0 °C, 12 h; (ii) NH_3_, CS_2_, EtOH, rt, 6 h; (iii) appropriate aromatic aldehyde, 60% NaOH, EtOH, 0 °C, 18 h; (iv) propargyl bromide, Na_2_CO_3_, NaI, acetone, rt, 6 h.

Scheme 1 depicts the steps in the synthesis of intermediates 6a–e. The procedure begins with the reaction of acetylacetone (1) with sulfuryl chloride in toluene at 0 °C for 12 hours, resulting in the selective chlorination of the active methylene group of acetylacetone to yield compound 2.^57^ The chlorinated product 2 reacts with ammonia and carbon disulfide in ethanol at room temperature for six hours, making the thiazole intermediate 3. This reaction has a moderate yield of 69%. Subsequently, thiazole 3 undergoes a reaction with the corresponding aldehydes 4a–e in basic conditions within ethanol at 0 °C for 18 hours. The Claisen–Schmidt condensation effectively generates thiazole chalcones 5a–e with yields varying from good to excellent (63% to 91%).^58^ The thiazole chalcones (5a–e) undergo *S*-alkylation with propargyl bromide in the presence of sodium carbonate and sodium iodide in acetone at ambient temperature for six hours. This phase provides the new intermediates 6a–e in good yields (78–85%).^59^

Compounds 6a–e were characterized *via*^1^H NMR, ^13^C NMR, and elemental analysis. The ^1^H NMR spectra of compounds 6a–e showed different signals, revealing their structural properties. The absence of the NH proton at *δ* ∼13.6 ppm in compounds 5a–e, together with the presence of an alkyne proton, is corroborated by a distinctive triplet signal at *δ* 3.34–3.36 ppm (*J* = 2.6 Hz), indicating the terminal acetylene group inserted during the *S*-alkylation process. The doublet signal at *δ* 4.18–4.20 ppm corresponds to the methylene group adjacent to the sulfur bridge. The ^13^C NMR spectra also showed characteristic signals including two distinct signals at *δ* 78.78–79.36 ppm and 74.97–75.46 ppm represent the two sp carbons of the alkyne group introduced during the *S*-alkylation step. The methylene group adjacent to the sulfur bridge appears at *δ* 21.55–22.04 ppm.

**Scheme 2:**
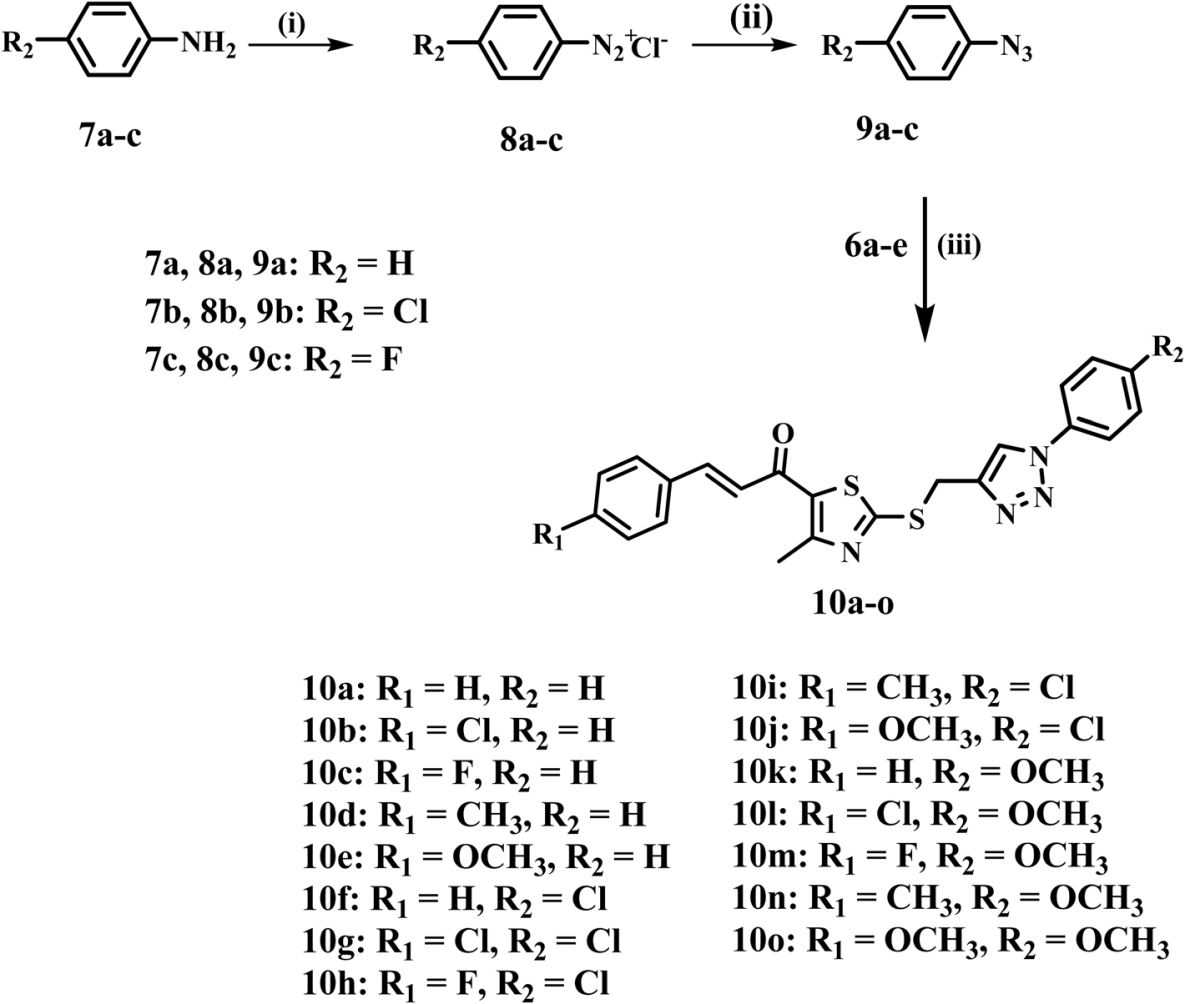
Synthesis of target compounds 10a–o. Reagents and conditions: (i) NaNO_2_, HCl, 0 °C, 30 min; (ii) NaN_3_, HCl, 0 °C, 2 h; (iii) CuSO_4_·5H_2_O, Na ascorbate, THF, rt, 5 h.

Scheme 2 describes the synthesis of the desired compounds 10a–o. The synthesis involves the diazotization of aniline derivatives 7a–c into their corresponding diazonium salts 8a–c using sodium nitrite and hydrochloric acid at 0 °C. The procedure occurs effectively under mild conditions. The diazonium salts are subsequently reacted with sodium azide in an acidic environment at the same temperature, yielding azide derivatives 9a–c.^48^ In the final phase, azides 9a–c are subjected to a reaction with propargyl-functionalized thiazole intermediates 6a–e under copper(i)-catalyzed azide–alkyne cycloaddition (CuAAC) conditions. The click reaction, catalyzed by CuSO_4_ and sodium ascorbate in THF at ambient temperature, proceeds efficiently, yielding the regioselective synthesis of the desired 1,2,3-triazole derivatives 10a–o with good yields (66–85%).

The structures of 10a–o were confirmed using ^1^H NMR, ^13^C NMR, and elemental analysis. The ^1^H NMR spectra of the 1,2,3-triazoles 10a–o exhibited a new singlet signal at *δ* ∼8.71–8.87 ppm for the triazole CH proton, accompanied by the absence of the terminal alkyne proton (*δ* ∼3.34–3.36 ppm), and a downfield shift of the methylene protons adjacent to sulfur to *δ* ∼4.70–4.73 ppm, indicative of the deshielding effect of the triazole ring. Furthermore, in the ^13^C NMR, the absence of alkyne carbon signals confirmed cycloaddition, while the methylene carbon was observed at *δ* ∼28 ppm. The distinctive proton signals for the thiazole methyl group and chalcone protons were mostly unchanged, so demonstrating the integrity of these moieties.

The ^1^H NMR spectrum of compound 10l, for example, exhibits characteristic signals, including a singlet at 2.70 ppm for the methyl group on the thiazole ring and another singlet at 3.82 ppm for the methoxy group. The methylene group is seen as a singlet at 4.70 ppm. The chalcone protons are apparent as two separate doublets at 7.39 ppm and 7.69 ppm. In contrast, the successful cyclization of the triazole ring is indicated by the CH proton, which appears as a singlet at 8.71 ppm. The ^13^C NMR spectrum of compound 10l showed a signal at *δ* 181.84 ppm corresponding to the carbonyl group, while the methoxy group gave a signal at *δ* 56.05 ppm. The methylene group appeared at *δ* 28.37 ppm, and the methyl group on the thiazole ring was observed at *δ* 18.75 ppm. Importantly, the disappearance of the alkyne signals at *δ* 79.30 ppm and 75.32 ppm, which were present in the starting material 6b used for the synthesis of 10l, confirms the successful cyclization.

### 3.2. Biology

#### 3.2.1. Cell viability assay

This study investigates the effects of the novel compounds 10a–o on normal cell lines to assess their safety. The normal human mammary gland epithelial MCF-10A cell line was used to assess the viability of the examined compounds. Cell viability was assessed using the MTT test after four days of incubation of MCF-10A cells with 50 µM of each examined compound.^49,50^Table 1 results indicate that none of the tested compounds exhibited cytotoxicity, with all compounds maintaining cell viability above 87% at a concentration of 50 µM.

**Table 1:** IC_50_ values of compounds 10a–o and erlotinib against four cancer cell lines

Comp.	Cell viability %	Antiproliferative activity IC_50_ ± SEM (nM)				
		A-549	MCF-7	Panc-1	HT-29	Average (GI_50_)
10a	90	45 ± 4	41 ± 3	48 ± 4	48 ± 4	46
10b	89	55 ± 5	51 ± 5	58 ± 5	58 ± 5	56
10c	88	30 ± 2	26 ± 2	30 ± 2	32 ± 2	30
10d	91	43 ± 3	38 ± 3	44 ± 4	42 ± 3	42
10e	90	27 ± 2	24 ± 2	28 ± 2	28 ± 2	27
10f	92	56 ± 5	54 ± 5	58 ± 5	58 ± 5	57
10g	89	62 ± 6	60 ± 6	64 ± 6	64 ± 6	63
10h	93	66 ± 6	62 ± 6	69 ± 6	68 ± 6	66
10i	91	36 ± 3	32 ± 3	38 ± 3	38 ± 3	36
10j	90	50 ± 4	46 ± 4	52 ± 4	52 ± 4	50
10k	87	24 ± 2	21 ± 2	26 ± 2	26 ± 2	24
10l	92	51 ± 4	47 ± 4	53 ± 5	54 ± 5	51
10m	87	40 ± 3	35 ± 3	40 ± 3	41 ± 3	39
10n	90	33 ± 3	30 ± 2	36 ± 3	36 ± 3	34
10o	91	31 ± 2	28 ± 2	32 ± 2	34 ± 3	31
Erlotinib	ND	30 ± 3	40 ± 3	30 ± 3	30 ± 3	33

#### 3.2.2. Antiproliferative assay

The MTT assay^51,52^ was employed to assess the antiproliferative efficacy of novel compounds 10a–o against four human cancer cell lines, including erlotinib as a control: colon (HT-29), pancreatic (Panc-1), lung (A-549), and breast (MCF-7) cancer cell lines. Table 1 displays the median inhibitory concentration (IC_50_) for each compound across all examined cancer cell lines, as well as the GI_50_ (mean IC_50_), which represents the average of the IC_50_ values for the four cancer cell lines.

The examined compounds 10a–o exhibited remarkable antiproliferative activity, with GI_50_ values ranging from 24 nM to 66 nM against the four assessed cancer cell lines, in contrast to the standard Erlotinib, which demonstrated a GI_50_ value of 33 nM. Remarkably, all evaluated compounds exhibited a greater affinity for the breast cancer (MCF-7) cell line compared to the other cell lines studied. Compounds 10c, 10e, 10k, 10m, 10n, and 10o were identified as the six most potent derivatives, with GI_50_ values ranging from 24 nM to 39 nM. In particular, compounds 10c, 10e, 10k, and 10o (GI_50_ values of 30, 27, 24, and 31, respectively) demonstrated greater potency than Erlotinib (GI_50_ value of 33 nM).

**Fig. 4:**
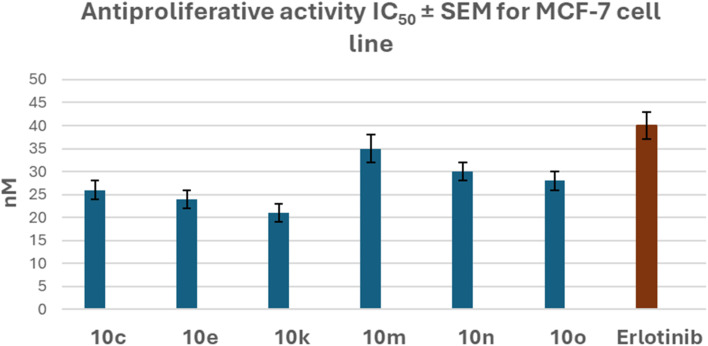
Antiproliferative activity (IC_50_ ± SEM) of compounds 10c, 10e, 10k, 10m, 10n, and 10o compared to Erlotinib against the MCF-7 cell line.

All six compounds exhibited greater potency than erlotinib against the MCF-7 breast cancer cell lines, with IC_50_ values ranging from 21 nM to 35 nM, compared to erlotinib's IC_50_ value of 40 nM, demonstrating at least a 1.2-fold increase in potency over erlotinib (Fig. 4).

Compound 10k (R_1_ = H, R_2_ = OCH_3_) exhibited the highest potency among the newly synthesized derivatives 10a–o, with a GI_50_ value of 24 nM, which is 1.4-fold superior to the reference erlotinib (GI_50_ = 33 nM) against the four tested cancer cell lines. Moreover, 10k had an IC_50_ value of 21 nM against the breast cancer (MCF-7) cell line, which was twice as potent as erlotinib's IC_50_ value of 40 nM.

Based on the findings, the type of substitution found on both phenyl rings at the chalcone (R_1_) and 1,2,3-triazole (R_2_) moieties appears to be critical for activity. For example, compounds 10l (R_1_ = Cl, R_2_ = OCH_3_), 10m (R_1_ = F, R_2_ = OCH_3_), 10n (R_1_ = CH_3_, R_2_ = OCH_3_), and 10o (R_1_ = OCH_3_, R_2_ = OCH_3_), all possessing the same backbone as compound 10k but differing in substituents (R_1_) at position 4 of the phenyl group within the chalcone moiety, exhibited GI_50_ values of 51, 39, 34, and 31, which are 2.2-, 1.6-, 1.4-, and 1.3-fold less potent than 10k, respectively. These data indicate that substituting the four-position of the phenyl group within the chalcone moiety reduces activity, and that substitutions with electron-donating groups (OCH_3_ and CH_3_) are more favorable for antiproliferative activity compared to electron-withdrawing groups (F and Cl).

Compound 10e (R_1_ = OCH_3_, R_2_ = H) exhibited the second-highest activity, with a GI_50_ of 27 nM, slightly less active than 10k (GI_50_ = 24 nM), but 1.2-fold more effective than the reference erlotinib (GI_50_ = 33 nM) against the four tested cancer cell lines. Compound 10e showed substantial antiproliferative activity against the MCF-7 breast cancer cell line, with an IC_50_ value of 24 nM, which is 1.7 times more potent than erlotinib (IC_50_ = 40 nM).

Also, the four-position substitution of the phenyl group in the 1,2,3-triazole moiety is essential for antiproliferative activity. Compounds 10j (R_1_ = OCH_3_, R_2_ = Cl) and 10o (R_1_ = OCH_3_, R_2_ = OCH_3_) share the same structure as 10e (R_1_ = OCH_3_, R_2_ = H) but possess different substituents at the *para* position of the phenyl group of the 1,2,3-triazole moiety (R_2_), exhibiting GI_50_ values of 50 and 31 nM, respectively, which are 1.9- and 1.2-fold less potent than 10e. The observations suggest that substituting the phenyl group at the four-position of the 1,2,3-triazole moiety lowered activity, with the following order of increasing activity: H > OCH_3_ > Cl. Moreover, these observations provide further evidence that replacement with electron-donating groups is more advantageous for the antiproliferative activity of this class of organic compounds than substitution with electron-withdrawing groups.

Another aspect to consider is the difference in potency between compounds 10a (R_1_ = H, R_2_ = H), 10e (R_1_ = OCH_3_, R_2_ = H), and 10k (R_1_ = H, R_2_ = OCH_3_). Compound 10a has a GI_50_ value of 46 nM, indicating a potency 1.9-fold and 1.7-fold lower than that of 10k and 10e. According to the results, at least one phenyl group must be replaced with a methoxy group, and the optimal combination for activity is an unsubstituted phenyl ring within the chalcone moiety and a *p*-methoxyphenyl group on the 1,2,3-triazole moiety.

Ultimately, compounds 10g (R_1_ = Cl, R_2_ = Cl) and 10h (R_1_ = F, R_2_ = Cl) had the lowest efficacy, evidenced by their GI_50_ values of 63 and 66 nM, respectively. This indicates that electron-withdrawing halogen atoms are not preferred for antiproliferative action.

#### 3.2.3. EGFR inhibitory assay

The EGFR-TK assay was employed to evaluate the inhibitory effectiveness of the most potent derivatives, 10c, 10e, 10k, 10m, 10n, and 10o against EGFR, using erlotinib as the reference compound.^53,54^ The IC_50_ values (nM) are displayed in Table 2. Compared to the reference erlotinib, which has an IC_50_ of 80 nM, the compounds investigated had GI_50_ values ranging from 69 to 87 nM, indicating a high level of EGFR inhibitory action.

**Table 2:** IC_50_ values of compounds 10c, 10e, 10k, 10m, 10n and 10o against EGFR, HER-2, BRAF^V600E^, and VEGFR-2^a^

Compound	EGFR inhibition IC_50_ ± SEM (nM)	HER-2 inhibition IC_50_ ± SEM (nM)	BRAF^V600E^ inhibition IC_50_ ± SEM (nM)	VEGFR-2 inhibition IC_50_ ± SEM (nM)
10c	77 ± 4	34 ± 2	76 ± 5	27 ± 1
10e	73 ± 4	31 ± 2	69 ± 5	20 ± 1
10k	69 ± 4	29 ± 1	63 ± 4	21 ± 1
10m	87 ± 5	43 ± 2	ND	ND
10n	83 ± 5	39 ± 2	ND	ND
10o	79 ± 4	37 ± 2	ND	ND
Erlotinib	80 ± 5	ND	ND	ND
Lapatinib	ND	26 ± 1	ND	ND
Vemurafenib	ND	ND	30 ± 3	ND
Sorafenib	ND	ND	ND	0.17 ± 0.001

a ND: Not Determined.

This *in vitro* experiment matches the antiproliferative assay. The most efficient antiproliferative derivatives, 10e and 10k, were also shown to be the best EGFR inhibitors, with IC_50_ values of 73 ± 4 and 69 ± 4 nM, respectively, which is 1.2-fold stronger than erlotinib as an EGFR inhibitor. Compounds 10c and 10o exhibited strong EGFR inhibitory activity, with IC_50_ values of 77 and 79 nM, respectively, which are comparable to that of erlotinib (IC_50_ = 80 nM). Ultimately, compounds 10m and 10n exhibited slightly reduced efficacy relative to erlotinib, with IC_50_ values of 83 and 87 nM, respectively. These findings suggest that the studied compounds 10c, 10e, 10k, and 10m are efficient antiproliferative agents that may operate as EGFR inhibitors.

#### 3.2.4. HER-2 inhibitory assay

An *in vitro* study was performed to evaluate the anti-HER-2 efficacy of derivatives 10c, 10e, 10k, and 10m–o. Lapatinib functioned as the standard medicine.^45^ The findings are displayed in Table 2. The findings demonstrated that the analyzed compounds had potent HER-2 inhibitory activity, with IC_50_ values ranging from 29 to 43 nM, compared to the reference lapatinib, which had an IC_50_ value of 26 nM.

Again, compounds 10e and 10k, the most effective antiproliferative and EGFR inhibitors, displayed the highest potency as HER-2 inhibitors with IC_50_ values of 29 ± 1 and 31 ± 2 nM, indicating comparable efficacy to the reference lapatinib. Compounds 10c, 10n, and 10o exhibited significant activity as HER-2 inhibitors, with IC_50_ values of 34, 39, and 37 nM, respectively, which is slightly less potent than compounds 10e and 10k, as well as the reference medication lapatinib. Eventually, compound 10m was identified as the least effective derivative of HER-2 inhibitors, exhibiting an IC_50_ value of 43 ± 2 nM, which is 1.7-fold less potent than lapatinib (IC_50_ value = 26 nM). These *in vitro* studies indicate that compounds 10c, 10e, and 10k exhibit significant antiproliferative activity and may function as dual EGFR/HER-2 inhibitors.

#### 3.2.5. BRAF^V600E^ inhibitory assay

An *in vitro* study assessed the potency of derivatives 10c, 10e, and 10k as BRAF^V600E^ inhibitors. Vemurafenib was used as a reference drug.^55^Table 2 summarizes the findings. The enzyme assay revealed that the evaluated compounds notably inhibited BRAF^V600E^, with IC_50_ values between 63 and 76 nM. All analyzed derivatives have higher IC_50_ values, indicating reduced potency compared to vemurafenib (IC_50_ = 30 ± 3). Compounds 10k and 10e displayed the most pronounced inhibitory effect against BRAF^V600E^ (IC_50_ = 63 and 69 nM, respectively), indicating at least a 2-fold decreased potency relative to the reference vemurafenib. Compound 10c had the lowest potency as a BRAF inhibitor, with an IC_50_ value of 76 nM, rendering it 2.5-fold less efficient than vemurafenib. The study's findings indicate that the compounds 10k and 10e exhibit moderate anti-BRAF^V600E^ activity, requiring structural modifications to enhance their efficacy against BRAF.

#### 3.2.6. VEGFR-2 inhibitory assay

The inhibitory effects of compounds 10c, 10e, and 10k on VEGFR-2 were evaluated by kinase assays, with sorafenib as the control drug.^21^Table 2 displays the results as IC_50_ values. The results indicated that the examined compounds significantly inhibited VEGFR-2, exhibiting IC_50_ values between 21 and 27 nM, compared to sorafenib, which demonstrated an IC_50_ of 0.17 nM. In every case, the compounds examined exhibited lower potency than sorafenib as VEGFR-2 inhibitors, although displayed more potency than EGFR and HER-2 inhibitors. Compounds 10k and 10e, identified as the most effective antiproliferative, EGFR, and HER-2 inhibitors, had substantial activity as VEGFR-2 inhibitors with IC_50_ values of 21 and 20 nM, indicating their potential as multi-kinase EGFR/HER-2/VEGFR-2 inhibitors.

### 3.3. Apoptotic markers assays

Apoptosis, or programmed cell death, is regarded as a crucial mechanism for the elimination of undesirable cells by the body.^60^ Consequently, inducing apoptosis in cancer cells will result in their inevitable demise and enhance the alleviation of cancer proliferation. A comprehensive understanding of apoptosis elucidates that it is influenced by the expression of caspases and Bcl-2 family proteins, which encompass both anti-apoptotic and pro-apoptotic members.^61^ The induction of apoptosis is regarded as one of the most effective approaches for targeting cancer.

Compounds 10e and 10k were assessed for their capacity to induce apoptosis in MCF-7 breast cancer cells by examining the expression of crucial apoptotic markers, namely Bcl-2, p53, and Bax. The findings are presented in Table 3. The Bcl-2 protein family, consisting of pro-apoptotic proteins (Bax) and anti-apoptotic proteins (Bcl-2), predominantly governs apoptosis. We examined the concentrations of Bcl-2 and Bax proteins in MCF7 breast cancer cells subjected to treatment with compounds 10e and 10k. Results indicate a significant 9-fold elevation in Bax levels and a 4.5-fold reduction in Bcl-2 levels for 10k compared to the control untreated cells. Furthermore, compound 10e exhibited a notable 8.6-fold elevation in Bax levels and a three-fold reduction in Bcl-2 levels. These observations suggest that apoptosis may play a role in the antiproliferative activities of the investigated compounds.

**Table 3:** Outcomes of apoptotic experiments for 10e and 10k against Bax, p53, and Bcl2

Compound no.	Bcl-2 (ng mL^−1^)	Bax (pg mL^−1^)	p53 (pg mL^−1^)
10e	1.30 ± 0.001	520 ± 3	360 ± 2
10k	1.10 ± 0.001	550 ± 3	385 ± 2
Control MCF-7 cell	5	60	65

The capacity for p53 overexpression to trigger apoptosis may clarify the common inactivation of p53 enzymes by cancer cells during transformation. The p53 levels in cancer cells subjected to compounds 10e and 10k exhibited a substantial increase, surpassing those of the untreated control cells by a minimum of 5-fold. This observation indicates that greater levels of the p53 protein may govern the apoptotic process in these new compounds.

### 3.4. Docking studies

#### 3.4.1. Docking into EGFR active site

In this study, molecular docking was performed to investigate the interactions of selected compounds (10k, 10e, and 10h) with the active site of the epidermal growth factor receptor (EGFR). The EGFR structure used for docking was retrieved from the Protein Data Bank (PDB ID: 1M17),^62^ where the co-crystallized ligand, erlotinib, served as a reference. Docking was performed using Autodock Vina,^63,64^ a widely used software for predicting protein-ligand binding poses and affinities. The docking results were then visualized using Discovery Studio Visualizer, which enabled a detailed examination of the ligand–receptor interactions.

**Fig. 5:**
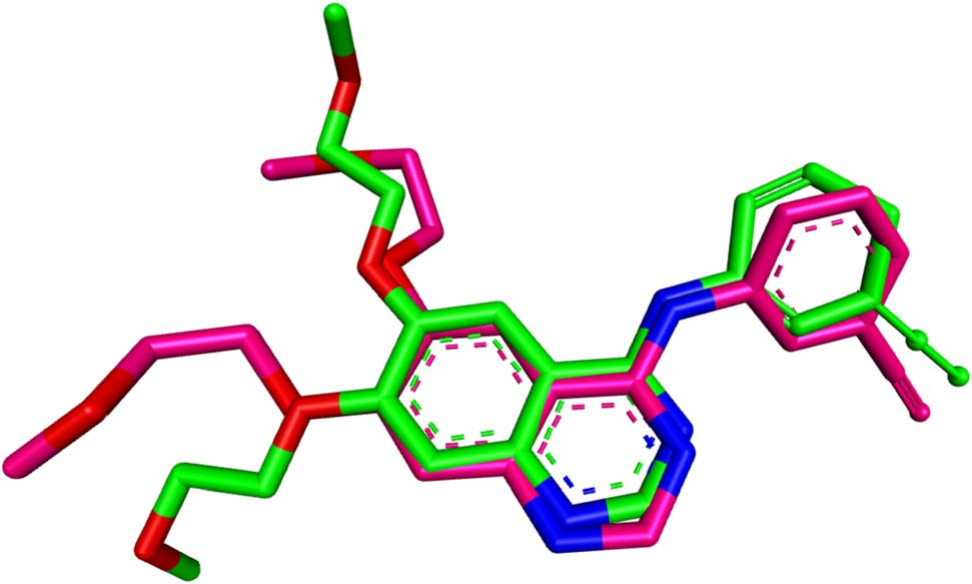
Superimposition of redocked (green) and co-crystallized (magenta) poses of erlotinib in the EGFR active site (RMSD = 1.4429 Å).

First, the docking procedure was validated by redocking erlotinib into the EGFR active site, comparing the predicted binding pose and affinity with the experimental values obtained from the crystal structure. The redocking results revealed a binding affinity of −9.4 kcal mol^−1^ and an RMSD of 1.4429 Å when compared to the original co-crystallized structure as shown in Fig. 5. The RMSD value of less than 2.0 Å confirmed that the docking protocol accurately reproduced the ligand's binding pose, ensuring the reliability and precision of the method. With this validation in place, the docking procedure was applied to the compounds of interest: 10k, 10e, and 10h.

**Fig. 6:**
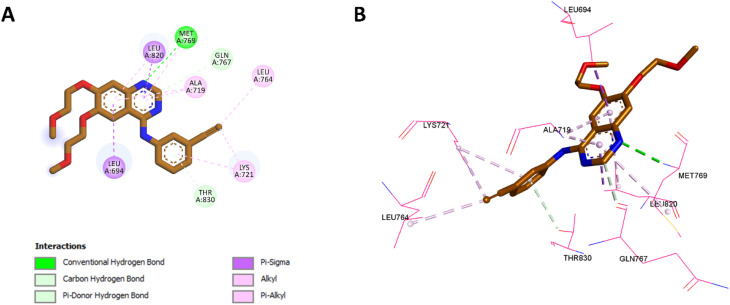
2D (A) and 3D (B) interactions of erlotinib within the EGFR active site.

**Fig. 7:**
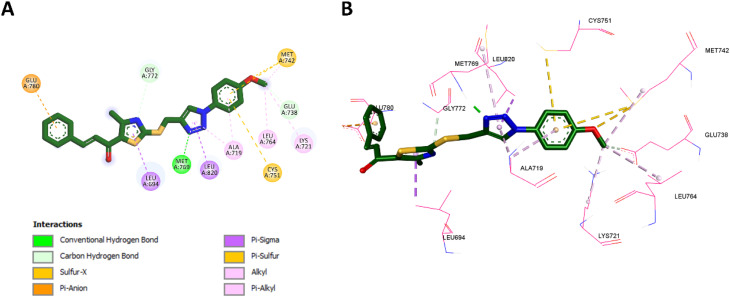
2D (A) and 3D (B) interactions of 10k within the EGFR active site.

For compound 10k (Fig. 7), the 1,2,3-triazole group formed a classical hydrogen bond with Met769, an interaction similar to that made by erlotinib's quinazoline ring (Fig. 6), which is critical for EGFR binding. The triazole ring of 10k also established hydrophobic interactions with Ala719 and Leu820, mirroring those of erlotinib with these residues. In addition, the benzene rings in 10k engaged in Pi-sulfur interactions with Cys751 and Met742, which were absent in erlotinib. The thiazole ring contributed to Pi-sigma interactions with Leu694, a feature shared with erlotinib. Notably, the methoxy group on the benzene ring of 10k made significant contributions by forming hydrophobic interactions with Leu764 and Lys721, similar to the interactions of the ethynyl group in erlotinib with these residues. Moreover, the methoxy group in 10k uniquely formed a non-classical hydrogen bond with Glu738, which erlotinib lacks. These distinctive interactions, including the Pi-sulfur contacts and additional hydrogen bonds with Glu738 and Gly772, enhance the binding affinity of 10k, accounting for its superior potency compared to erlotinib.

**Fig. 8:**
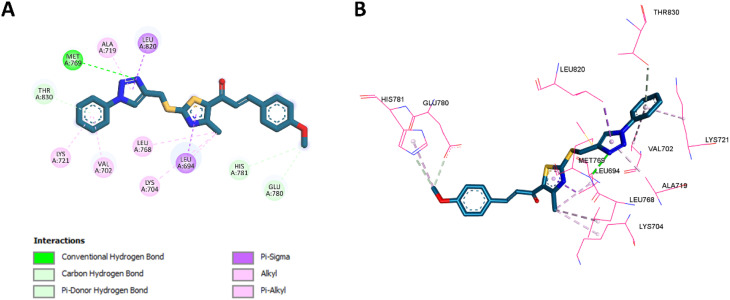
2D (A) and 3D (B) interactions of 10e within the EGFR active site.

In the case of compound 10e (Fig. 8), the 1,2,3-triazole group formed a classical hydrogen bond with Met769, mimicking the essential role of erlotinib's quinazoline ring in EGFR inhibition. Like 10k, the triazole ring in 10e displayed hydrophobic interactions with Ala719 and Leu820, similar to those made by erlotinib's quinazoline ring. The thiazole ring of 10e showed Pi-sigma interactions with Leu694, a conserved feature also observed in erlotinib. Importantly, the methoxy group in 10e played a critical role by forming non-classical hydrogen bonds with His781 and Glu780, interactions absent in erlotinib, underscoring the significance of the methoxy group in enhancing binding. Additionally, 10e formed a non-classical (Pi-donor) hydrogen bond with Thr830, a novel interaction not observed in erlotinib. These unique contributions, particularly the hydrogen bond with Thr830 and the stabilizing interactions involving the methoxy group with His781 and Glu780, likely account for 10e's improved potency relative to erlotinib. However, it remains slightly less potent than 10k.

**Fig. 9:**
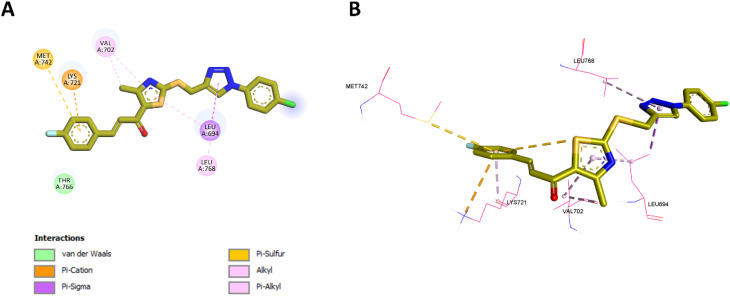
2D (A) and 3D (B) interactions of 10h within the EGFR active site.

Moving to compound 10h (Fig. 9), the 1,2,3-triazole group exhibited hydrophobic (Pi-sigma) interactions with Leu694, similar to the interactions of erlotinib's quinazoline ring with the same residue. However, unlike 10k and 10e, 10h adopted a distinct binding pose that prevented its triazole ring from forming the crucial hydrogen bond with Met769, an interaction vital for binding and present in erlotinib and the more potent compounds. The absence of this key interaction likely contributes to 10h's lower potency. Furthermore, the lack of a stabilizing methoxy group, which was replaced by chloro and fluoro groups that failed to engage in significant interactions, further weakened 10h's binding affinity, resulting in reduced activity compared to the other compounds.

#### 3.4.2. Docking into HER-2 active site

**Fig. 10:**
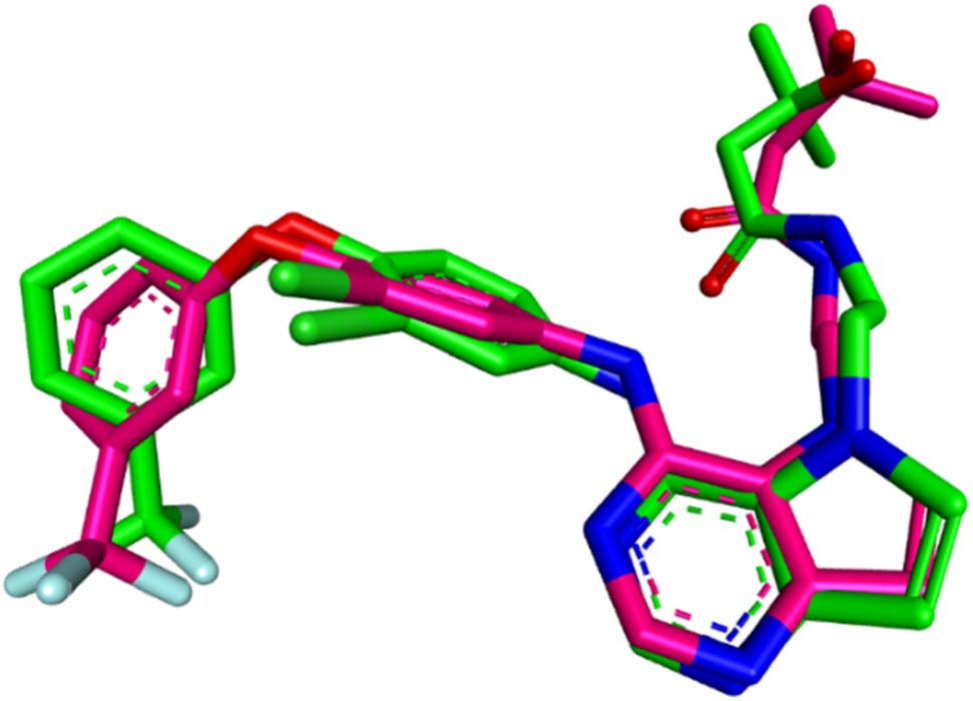
Superimposition of redocked (green) and co-crystallized (magenta) poses of TAK-285 in the HER-2 active site (RMSD = 1.0845 Å).

To validate the docking procedure for HER-2, the co-crystallized ligand TAK-285 (ref. 65) was redocked into the HER-2 active site. The redocking results yielded an RMSD of 1.0845 Å and a binding affinity of −8.3 kcal mol^−1^, indicating that the docking protocol accurately reproduced the ligand's binding pose and affinity. This validation confirms the reliability of the method for docking compounds into the HER-2 active site. The superimposition of the redocked TAK-285 and the co-crystallized pose is shown in Fig. 10.

In addition to validating the docking procedure, we also docked compound 10k, which demonstrated the best binding profile in HER-2, alongside the reference compound lapatinib. The docking results revealed binding affinities of −8.8 kcal mol^−1^ for 10k and −9.2 kcal mol^−1^ for lapatinib, suggesting comparable binding strengths. *In vitro* assays also confirmed that the HER-2 inhibition activity of 10k was similar to that of lapatinib, supporting the docking predictions.

Several interactions between compound 10k and HER-2 were found to be similar to those with lapatinib. Specifically, the nitrogen atom of the 1,2,3-triazole moiety of compound 10k establishes a hydrogen bond with Met801, paralleling the interaction between the nitrogen atom of lapatinib's quinazoline ring and the same residue, which is crucial for HER-2 suppression. Furthermore, the triazole ring of 10k forms hydrophobic interactions with Val734 and Ala751, similar to the hydrophobic interactions established by the quinazoline ring of lapatinib with these residues. The benzene ring of 10k also participates in hydrophobic interactions with Leu796, similar to those observed with the benzene ring of lapatinib.

**Fig. 11:**
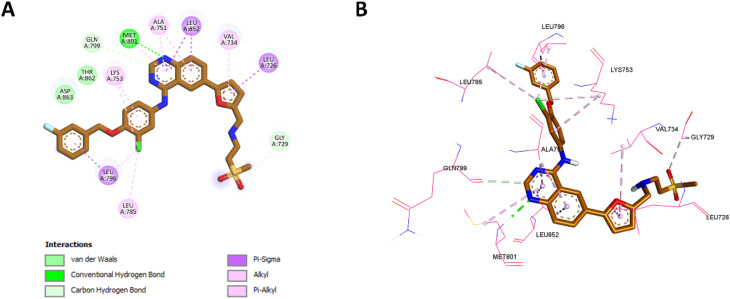
2D (A) and 3D (B) interactions of lapatinib within the HER-2 active site.

**Fig. 12:**
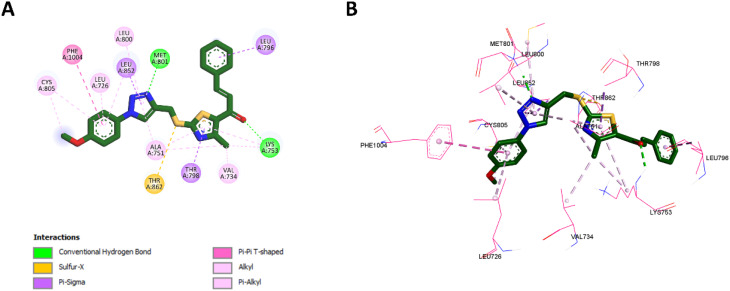
2D (A) and 3D (B) interactions of 10k within the HER-2 active site.

The thiazole ring of 10k forms hydrophobic contacts with Lys753, a feature also observed with lapatinib's benzene ring. Furthermore, compound 10k forms an additional characteristic hydrogen bond with Lys753, an interaction that is not present in lapatinib, which could further stabilize its binding. Notably, the benzene ring of 10k also engages in a unique pi–pi T-shaped interaction with Phe1004, a feature absent in lapatinib. The methoxy group of 10k forms additional hydrophobic interactions with Cys805, further distinguishing it from lapatinib. The 2D and 3D interactions of lapatinib within the HER-2 active site are depicted in Fig. 11, while the corresponding interactions of compound 10k are shown in Fig. 12.

Following the EGFR and HER-2 docking analyses, compounds 10k and 10e were further evaluated for their binding to VEGFR-2 (PDB ID: 3U6J),^66^ given their promising VEGFR-2 inhibition observed *in vitro*. The docking scores indicated strong binding affinities of −9.30 kcal mol^−1^ for 10k and −9.34 kcal mol^−1^ for 10e, compared to −10.15 kcal mol^−1^ for the reference inhibitor sorafenib.

**Fig. 13:**
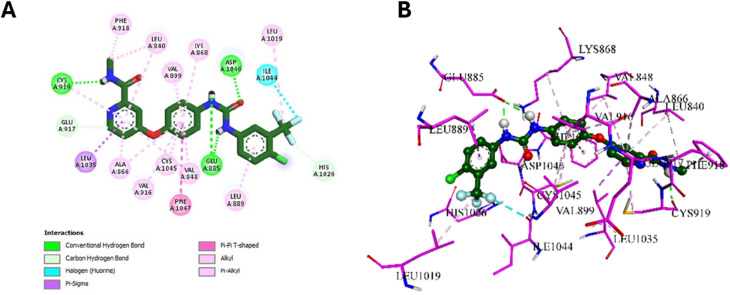
2D (A) and 3D (B) interactions of sorafenib within the VEGFR-2 active site.

**Fig. 14:**
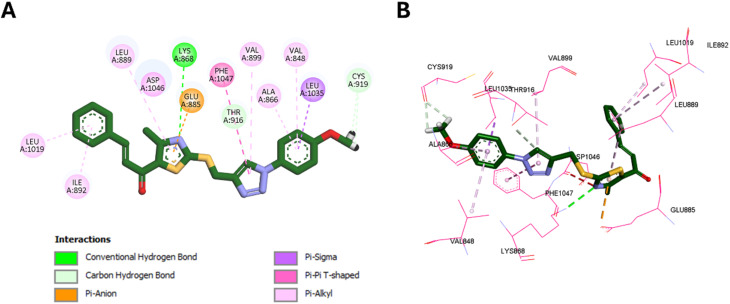
2D (A) and 3D (B) interactions of 10k within the VEGFR-2 active site.

For 10k, several interactions were conserved with those observed in sorafenib, including hydrophobic contacts with Leu889, Val848, Leu1035, and Ala866, as well as hydrogen bonding with Lys868. These collaborative interactions demonstrate a comparable binding orientation inside the VEGFR-2 active region and align with its documented *in vitro* efficacy. In addition, 10k established unique interactions do not present in sorafenib, including an electrostatic interaction with Glu885 *via* the thiazole nitrogen, and C–H bonding with Cys919 through its methoxy group, whereas sorafenib forms a classical H-bond at this position. Moreover, 10k formed additional hydrophobic contacts with Val898 and Ile1044, residues not engaged by sorafenib. These added interactions likely enhance ligand stabilization and correlate well with its strong VEGFR-2 inhibition in biological assays. Visual summaries of the interactions exhibited by sorafenib and 10k are presented in Fig. 13 and 14, respectively.

**Fig. 15:**
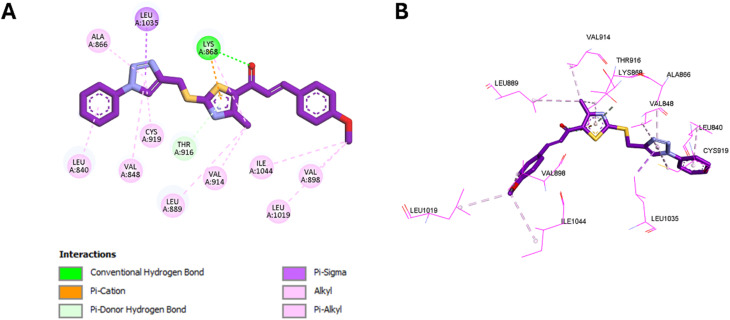
2D (A) and 3D (B) interactions of 10e within the VEGFR-2 active site.

Similarly, 10e retained multiple conserved interactions, such as hydrogen bonding with Lys868 and hydrophobic contacts with Leu889, Val848, Leu1035, and Ala866, aligning with its binding orientation relative to sorafenib. However, 10e also introduced novel interactions, including hydrophobic engagement with Cys919 and Val914, and unique contacts involving its methoxy and thiazole moieties. Notably, unlike 10k, 10e did not interact with Val898 or Ile1044, which may account for its slightly reduced potency *in vitro* despite a marginally higher docking score. These interactions are illustrated in Fig. 15.

#### 3.4.3. ADME prediction

**Fig. 16:**
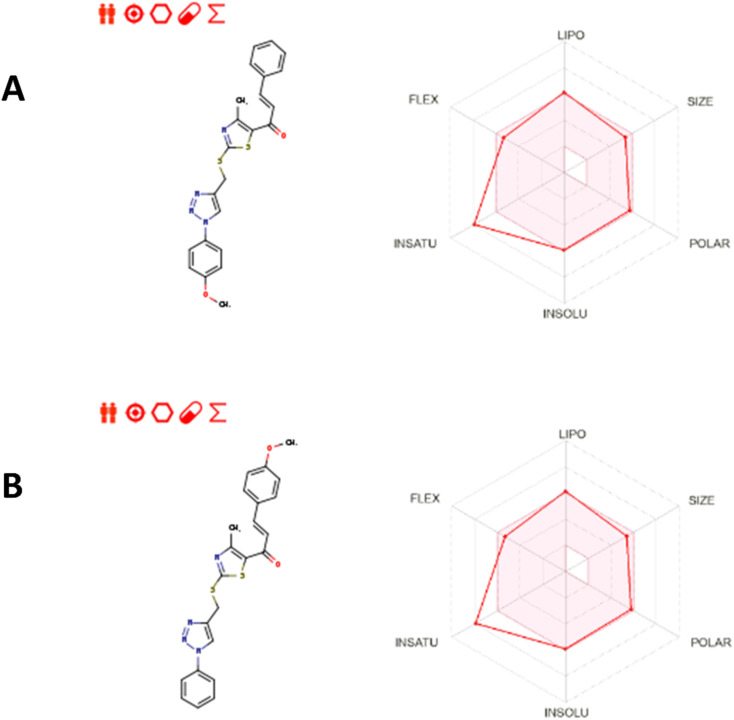
(a) Bioavailability radar plot of compound 10k; (b) bioavailability radar plot of compound 10e.

The physicochemical and pharmacokinetic properties of compounds 10k and 10e were calculated using the SwissADME tool,^67,68^ which provides a comprehensive analysis of drug-likeness, solubility, lipophilicity, and absorption characteristics based on molecular structure. Both compounds have identical molecular formulas (C_23_H_20_N_4_O_2_S_2_) and molecular weights (448.56 g mol^−1^), indicating similar molecular sizes and structures, which could contribute to their comparable pharmacological behavior. As shown in Fig. 16, the bioavailability radar plots for compounds 10k and 10e reflect their promising physicochemical profiles. Both compounds exhibit moderate lipophilicity, solubility, and polarity, suggesting they are well-suited for membrane permeability and targeted delivery to specific tissues or cells. The radar plots indicate that both compounds fall within acceptable ranges for most pharmacokinetic parameters, with moderate potential for oral bioavailability.

Both compounds exhibit moderate lipophilicity. Specifically, compound 10k has a calculated log *P* of 4.54, while compound 10e shows a very similar value of 4.52. This close similarity in lipophilicity suggests that both compounds are likely to display comparable membrane permeability and distribution behavior *in vivo*. Such log *P* values are within the range generally associated with effective cell membrane penetration, supporting their potential for efficient target engagement, particularly in hydrophobic binding pockets of EGFR and HER-2. However, it is worth noting that the compounds exhibit low gastrointestinal (GI) absorption. This is typically considered a disadvantage, but in the context of these compounds, it might be useful. Low GI absorption could limit systemic exposure and potential off-target effects, thereby increasing specificity for the targeted cancer cells. Moreover, compounds with low absorption may sometimes be more effective at localizing to specific tissues, such as tumors, where the drug may accumulate in higher concentrations.

The water solubility results show that both compounds are moderately soluble according to the ESOL model but poorly soluble in the Ali and SILICOS-IT models. While poor solubility can pose challenges to oral bioavailability, it can be leveraged in drug delivery systems that enhance solubility, such as nanoparticles or liposomes, thereby mitigating this issue.

Both compounds demonstrate a bioavailability score of 0.55, indicating moderate potential for oral bioavailability. Furthermore, the compounds do not violate Lipinski's Rule of Five, indicating that they are likely to have favorable pharmacokinetic profiles in terms of absorption, distribution, metabolism, and excretion (ADME). However, both compounds violate Muegge's rule due to an XLOGP3 greater than 5, which suggests that they may exhibit some degree of poor solubility or permeability, aligning with their GI absorption and solubility profiles.

Regarding their interaction with drug-metabolizing enzymes, both compounds inhibit several cytochrome P450 enzymes, such as CYP2C19, CYP2C9, and CYP3A4. These interactions could affect the metabolism of co-administered drugs, which may be a consideration in combination therapies. However, the lack of inhibition of other P450 enzymes and transporters, such as P-glycoprotein (P-gp), suggests that these compounds may have a relatively safe metabolic profile compared to other drugs that interact more broadly with these systems.

Additionally, both compounds are free from PAINS (pan-assay interference compounds) alerts, which is a positive indicator of their chemical quality. Although both compounds exhibit lead-likeness violation due to their molecular weight and rotatable bonds, they are still considered synthetically accessible with ease (accessibility scores close to 4), suggesting that they could be further developed into drug candidates with practical synthesis routes.

Given that compounds 10k and 10e have already been demonstrated to effectively target the EGFR and HER-2 pathways through *in vitro* assays, their physicochemical and pharmacokinetic properties further strengthen their potential as promising candidates for therapeutic development. Their lipophilicity and moderate bioavailability suggest they are well-suited for targeting specific tissues or cells, including those expressing EGFR and HER-2, making them effective in treating cancers driven by these pathways.

### 3.5. Structure–activity relationship analysis (SAR analysis)

The following points can be noted regarding the SAR of novel compounds 10a–o:

(1) The 1,2,3-triazole moiety is crucial for EGFR inhibitory activity, as it establishes a hydrogen bond with Met769, similar to the contact formed by erlotinib's quinazoline ring, which is vital for EGFR binding.

(2) Regarding HER-2 activity, the nitrogen atom of the 1,2,3-triazole moiety forms a hydrogen bond with Met801, analogous to the interaction between the nitrogen atom of lapatinib's quinazoline ring and the same residue, which is essential for HER-2 inhibition.

(3) The thiazole ring establishes a hydrogen bond with Lys868, which is crucial for the suppression of VEGFR-2 activity.

(4) Furthermore, the phenyl group of the 1,2,3-triazole moiety participated in several hydrophobic interactions within the EGFR, VEGFR-2, and HER-2 sites, thereby enhancing binding to the active sites of these enzymes and thereby increasing activity.

(5) The substitution pattern at the *para* position of the phenyl group in the 1,2,3-triazole moiety (R_2_) significantly influences activity, with the methoxy group being the most favored due to hydrophobic interactions with Leu764 and Lys721, analogous to the ethynyl group interactions of erlotinib with these residues or with Cys805 in the HER-2 active site. The methoxy group formed a significant hydrogen bond with Cys919 in VEGFR-2 active site.

(6) The thiazole ring facilitated Pi-sigma interactions with Leu694, a characteristic also present in erlotinib.

(7) The carbonyl oxygen of the chalcone moiety establishes a hydrogen bond with Lys753, an interaction absent in lapatinib, which may further strengthen the binding within the HER-2 active site.

## 4. Conclusion

Compounds 10c, 10e, 10k, 10m, 10n, and 10o demonstrated remarkable antiproliferative efficacy and a favorable safety profile, making them the most active hybrids in this experiment. The MTT antiproliferative experiment revealed that MCF-7 breast cancer cells were the most appropriate targets for these agents. Compounds 10e and 10k have the highest anti-breast cancer activity, with IC_50_ values of 24 and 21 nM against the MCF-7 cell line, respectively. Notably, they exhibited the highest efficacy as multi-kinase EGFR/HER-2/VEGFR-2 inhibitors, while demonstrating moderate anti-BRAF^V600E^ action. In molecular modeling studies, compounds 10e and 10k exhibited significant binding affinity for the crucial amino acids Met769, Glu738, Leu764, and Lys721 in the EGFR kinase active site, as well as Met801 in the HER-2 kinase pocket. Additionally, their binding modes within the VEGFR-2 active site involved key residues such as Lys868, Glu885, and Cys919, further supporting their VEGFR-2 inhibition profile. Moreover, the active compounds conformed to Lipinski's rule of five, exhibiting satisfactory ADMET data that demonstrated their potential for subsequent stages of drug discovery. The active chemicals and their biological effects, along with modeling studies, may serve as prototypes for future research and development. Further research is needed to determine their *in vivo* efficacy and toxicity. Future studies will focus on enhancing BRAF^V600E^ inhibition while maintaining the compounds' anticancer properties. Also, we will empirically ascertain the log *P* values along with other physicochemical parameters for a representative subset of compounds and compare them with the calculated values.

## Conflicts of interest

The authors stated there were no potential conflicts of interest.

## Supplementary Material

RA-016-D5RA07556D-s001

## Data Availability

The authors assert that any data supporting this study can be found in the supplementary information (SI). Supplementary information is available. See DOI: https://doi.org/10.1039/d5ra07556d.
