# Supplementary material for: Design, synthesis, in silico studies, and antiproliferative activity of a novel series of thiazole/1,2,3-triazole hybrids as apoptosis inducers and multi-kinase inhibitors endowed with anti-breast cancer activity
Source: RSC Adv. 2026 Jan 15;16(4):3349–67. doi: 10.1039/d5ra07556d (PMC12805330; doi:10.1039/d5ra07556d)
Supplement: RA-016-D5RA07556D-s001 [file RA-016-D5RA07556D-s001.pdf]

Supplementary Information (SI) for RSC Advances.
This journal is © The Royal Society of Chemistry 2026

# Design, synthesis, *in silico* studies, and antiproliferative activity of novel series of thiazole/1,2,3-triazole hybrids as apoptosis inducers and multi-kinase inhibitors endowed with anti-breast cancer activity

Fatma A.M. Mohamed*[1], Saleha Y. M. Alakilli[2], Hassan H. Alhassan[3], Sara Osman Yousif [1], Eid Alatwi[4], Hesham A.M. Gomaa[4], Heba Abu Alrub[1], Bandar A. Alyami[5], Ahmed H. Abdelhafez[6], Stefan Bräse[7]*, Bahaa G. M. Youssif*[8].

[1]Department of Clinical Laboratory Sciences, College of Applied Medical Sciences at Al-Qurayyat, Jouf University, Al-Qurayyat 77454, Saudi Arabia; [2]Department of Biological Sciences, Faculty of Sciences, King Abdulaziz University, Jeddah 23761, Saudi Arabia; [3]Department of Clinical Laboratories Sciences, College of Applied Medical Sciences, Jouf University, Sakaka 72388, Saudi Arabia; Department of [4]Pharmacology, College of Pharmacy, Jouf University, Sakaka 72388, Saudi Arabia; [5]Department of Pharmaceutical Chemistry, College of Pharmacy, Najran University, Najran, Saudi Arabia; [6]PharmaHelp Foundation Inc., Falls Church, Virginia, USA; [7]Institute of Biological and Chemical Systems, IBCS-FMS, Karlsruhe Institute of Technology, 76131 Karlsruhe, Germany; [8]Department of Pharmaceutical Organic Chemistry, Faculty of Pharmacy, Assiut University, Assiut-71526, Egypt.

**To whom correspondence should be addressed:*

**Fatma A.M. Mohamed**, Ph.D. Department of Clinical Laboratory Sciences, College of Applied Medical Sciences at Al-Qurayyat, Jouf University.

E-mail address: fatmaahmed@ju.edu.sa

**Bahaa G. M. Youssif**, Ph.D. Pharmaceutical Organic Chemistry Department, Faculty of Pharmacy, Assiut University, Assiut 71526, Egypt.

Tel.: +201044353895; E-mail address: bgyoussif2@gmail.com

**Stefan Bräse**

Institute of Biological and Chemical Systems, IBCS-FMS, Karlsruhe Institute of Technology, 76131 Karlsruhe, Germany. E-mail: braese@kit.edu

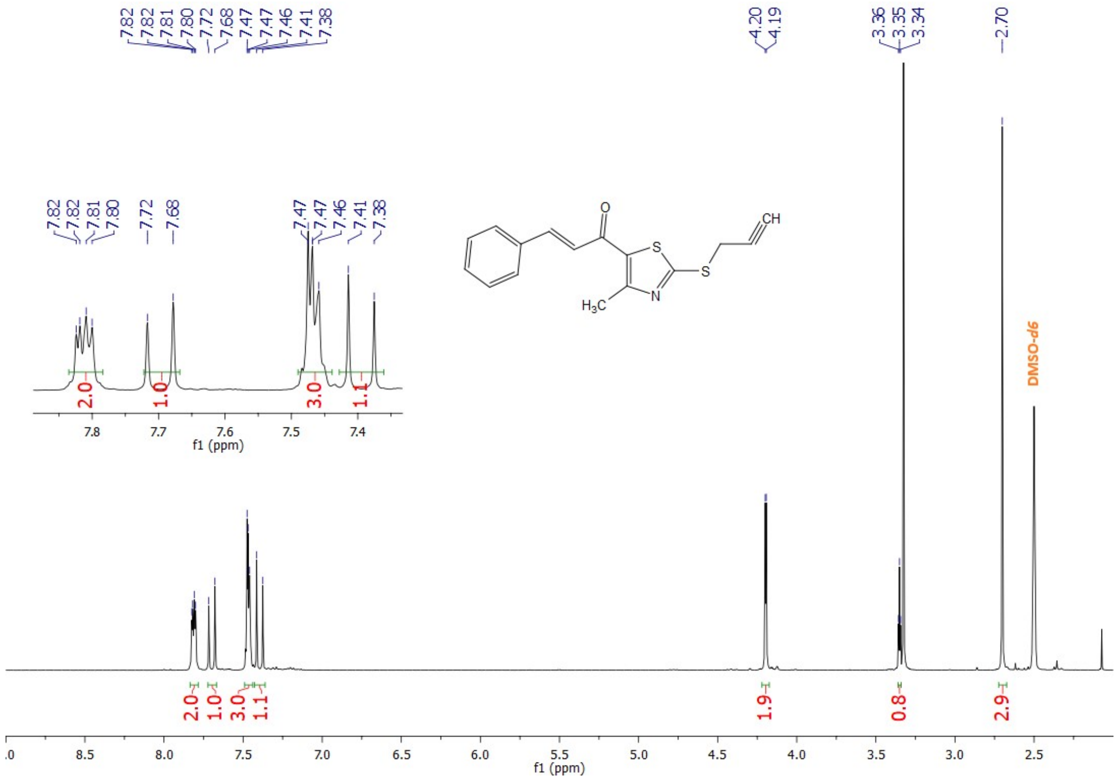


**Figure S1**. $^{1}$H NMR (400 MHz, DMSO-$d_6$) spectrum of **6a**

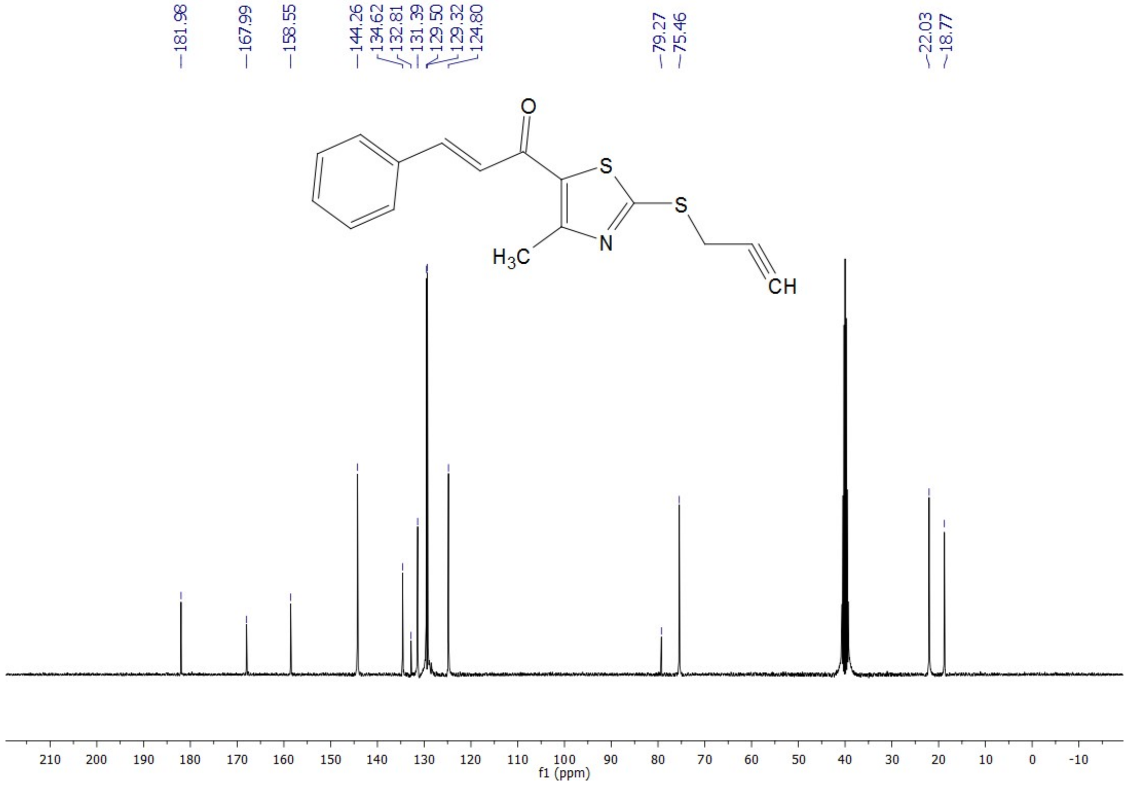


**Figure S2**. $^{13}$C NMR (100 MHz, DMSO-$d_6$) spectrum of **6a**

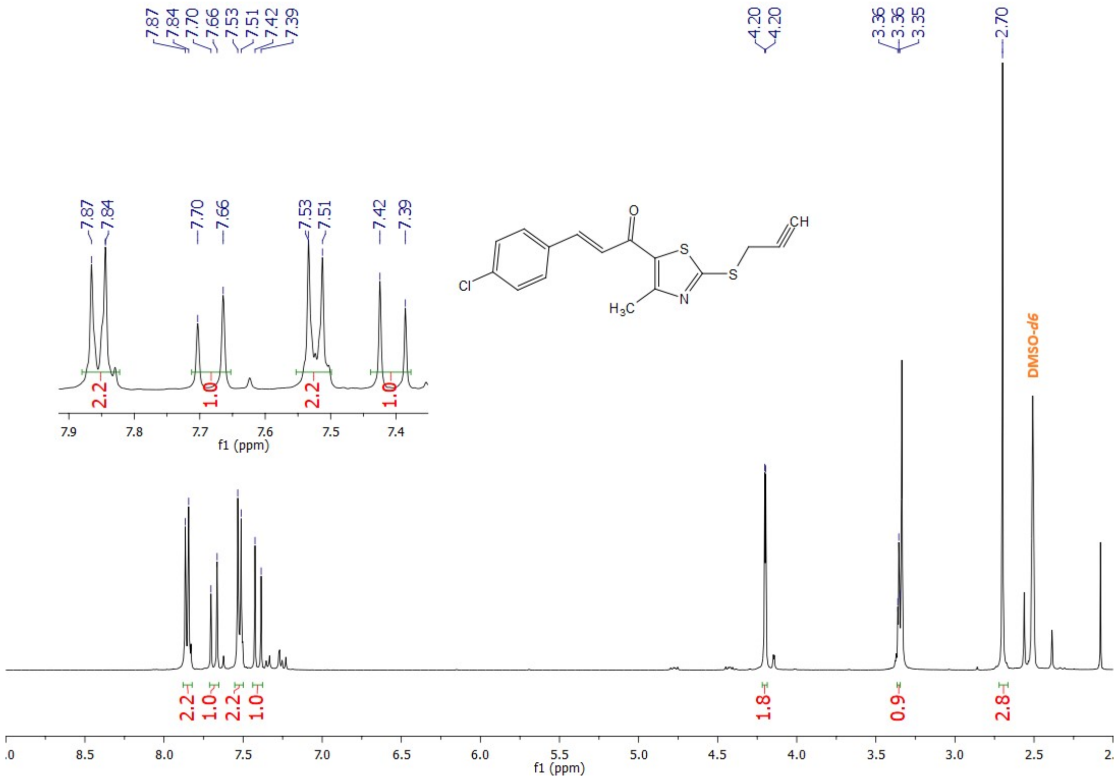


**Figure S3**. $^{1}$H NMR (400 MHz, DMSO-$d_6$) spectrum of **6b**

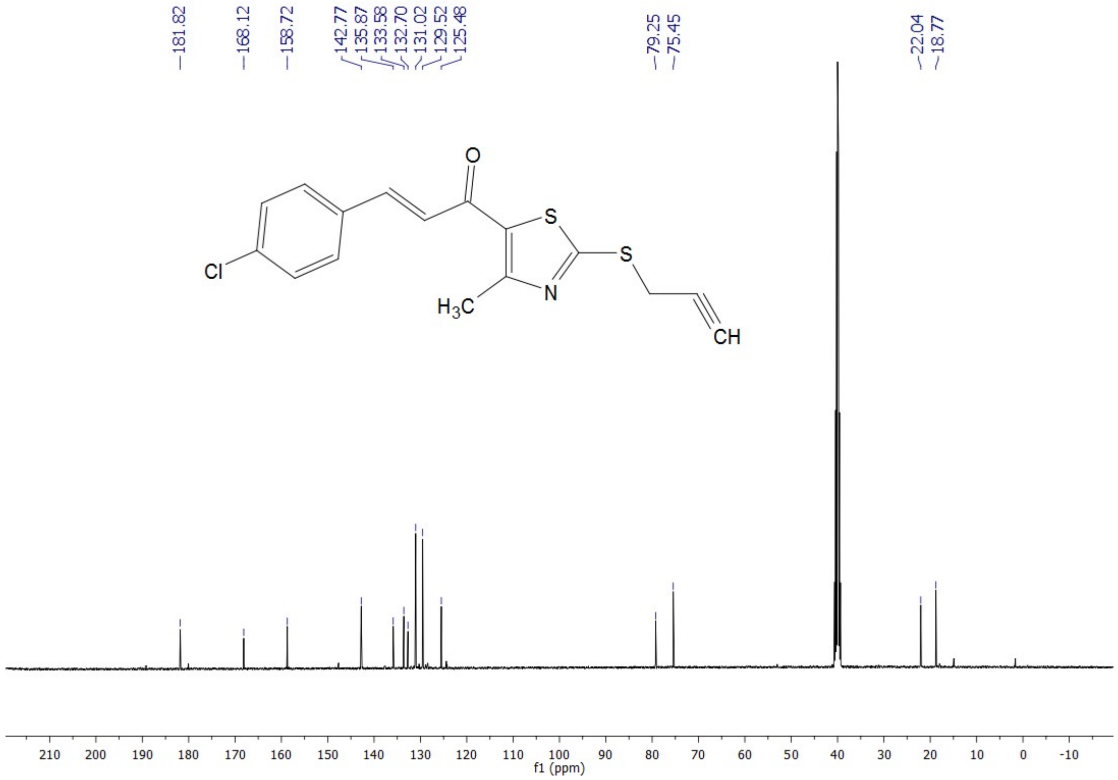


**Figure S4**. $^{13}$C NMR (100 MHz, DMSO-$d_6$) spectrum of **6b**

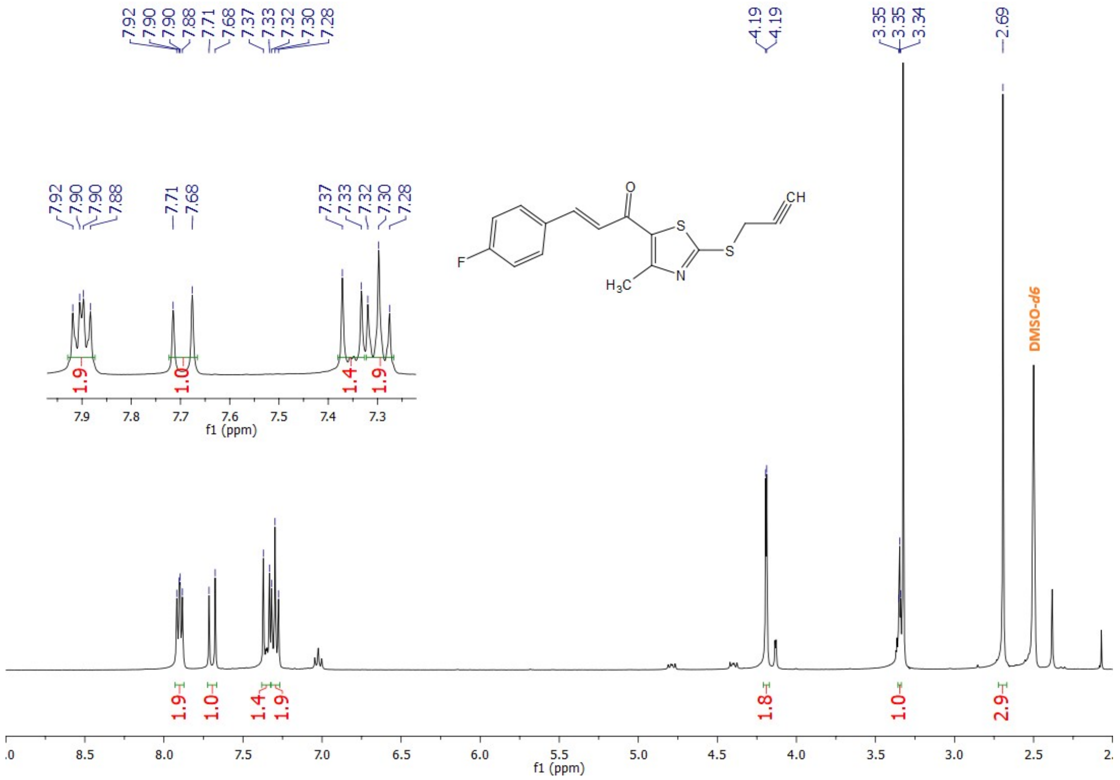


**Figure S5**. $^{1}$H NMR (400 MHz, DMSO-$d_6$) spectrum of **6c**

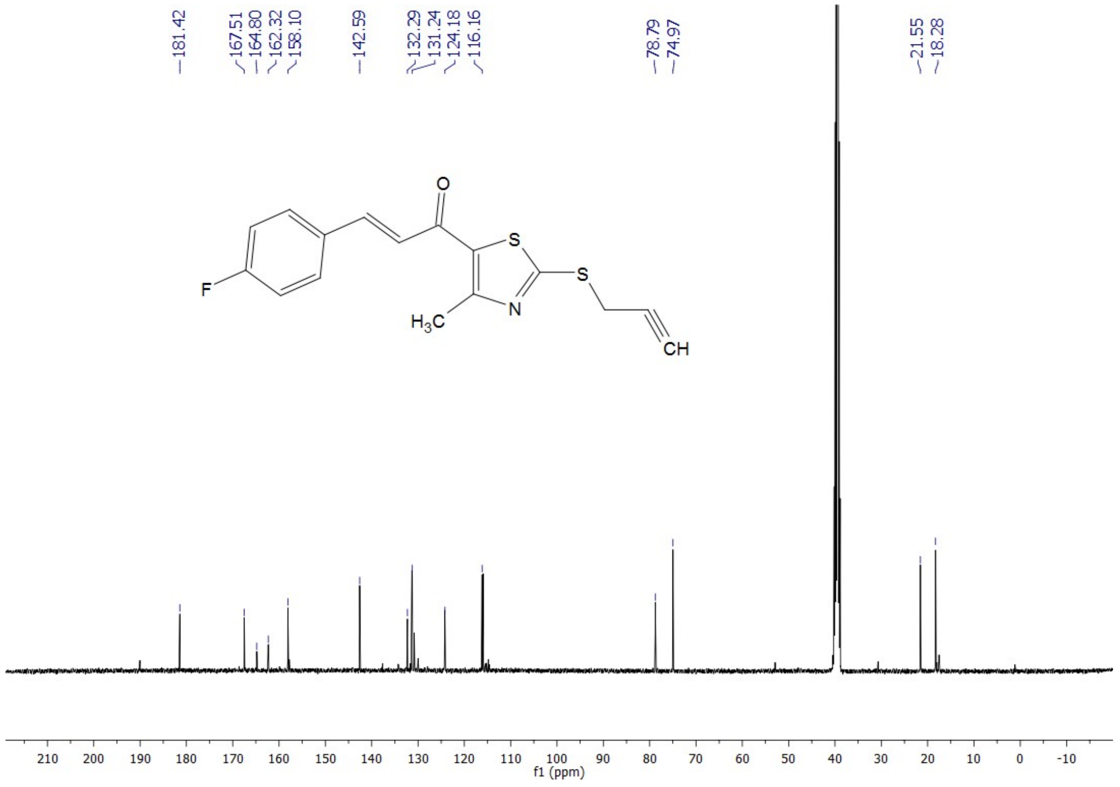


**Figure S6**. $^{13}$C NMR (100 MHz, DMSO-$d_6$) spectrum of **6c**

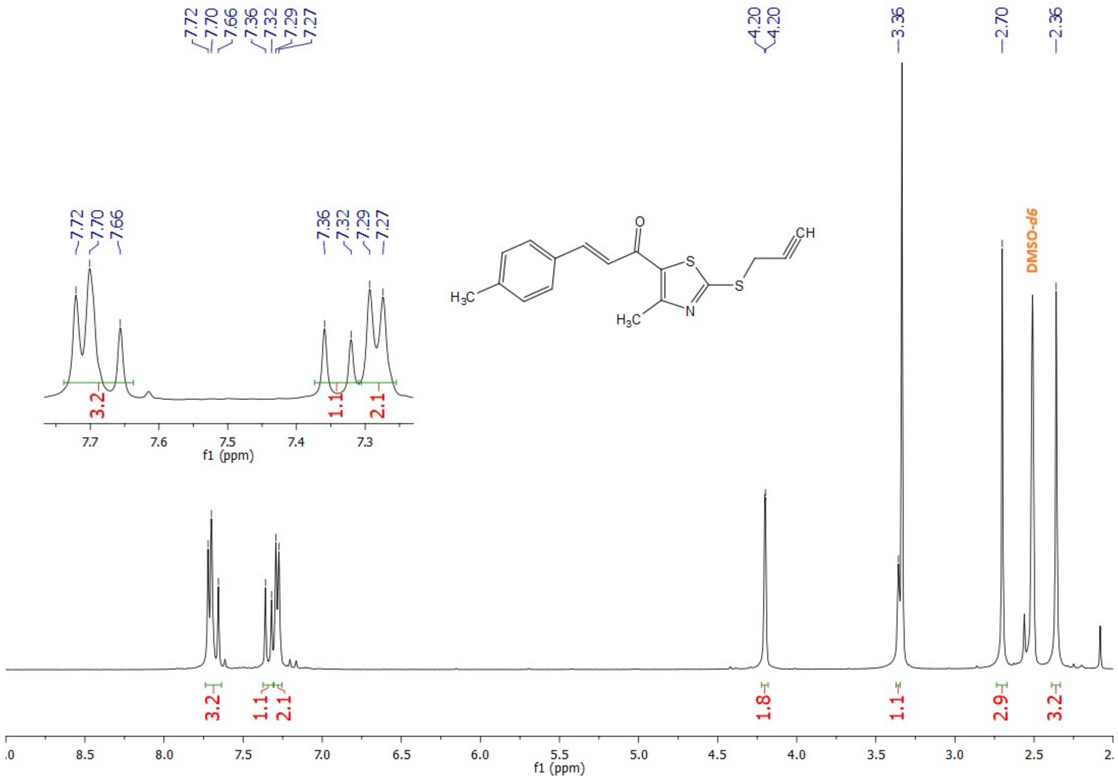


**Figure S7**. $^{1}$H NMR (400 MHz, DMSO-$d_6$) spectrum of **6d**

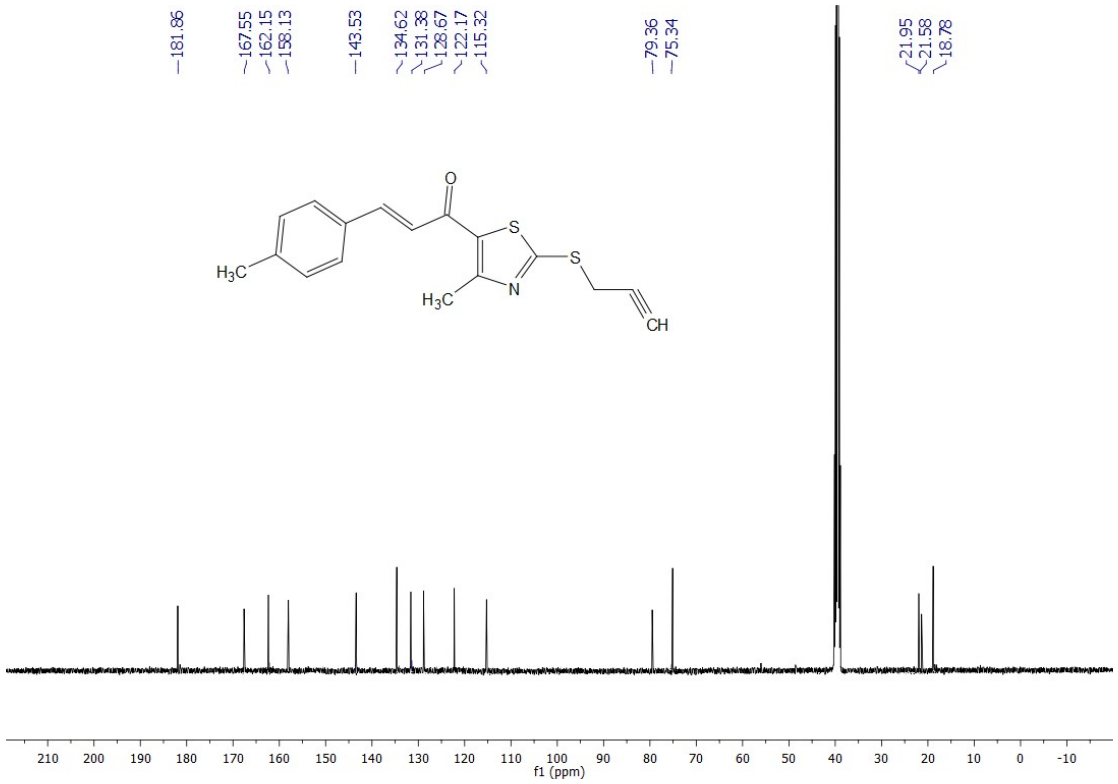


**Figure S8**. $^{13}$C NMR (100 MHz, DMSO-$d_6$) spectrum of **6d**

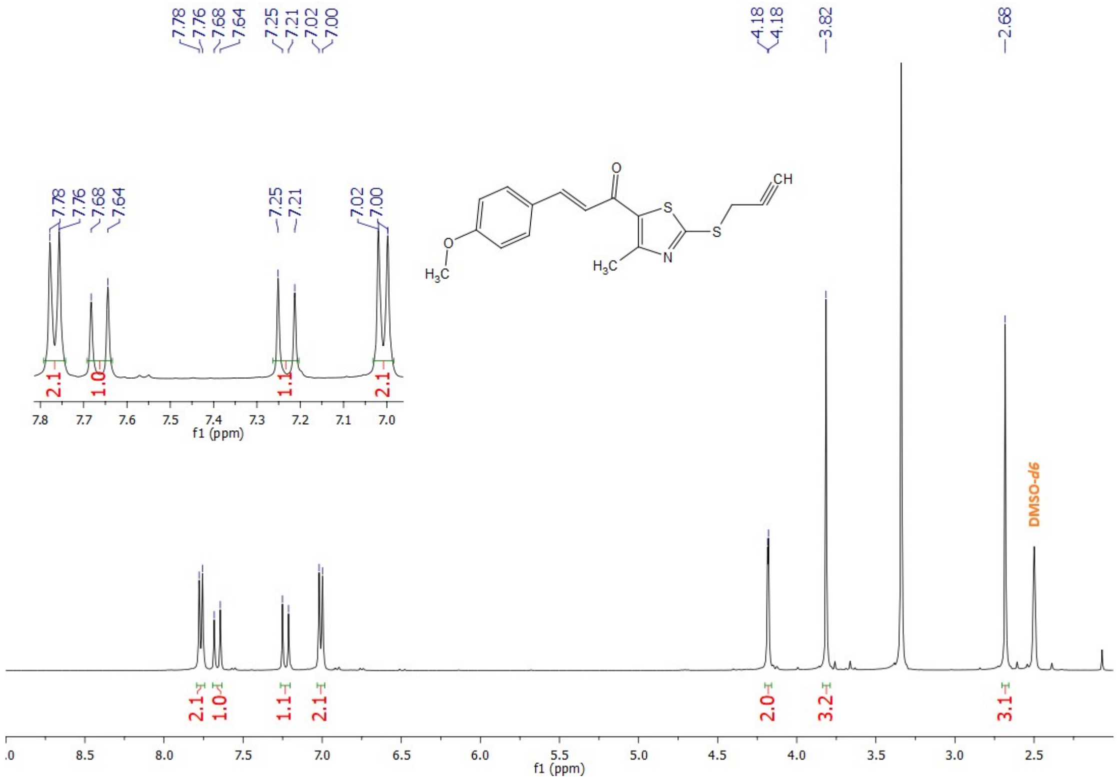


**Figure S9**. $^{1}$H NMR (400 MHz, DMSO-$d_6$) spectrum of **6e**

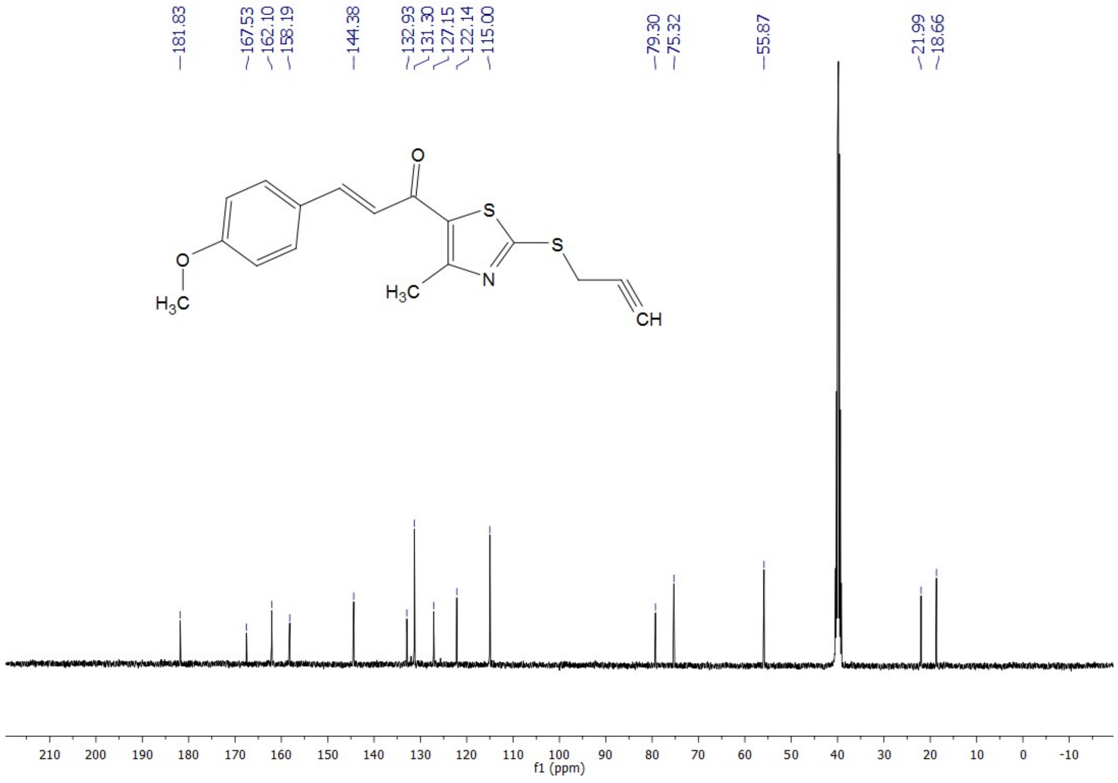


**Figure S10**. $^{13}$C NMR (100 MHz, DMSO-$d_6$) spectrum of **6e**

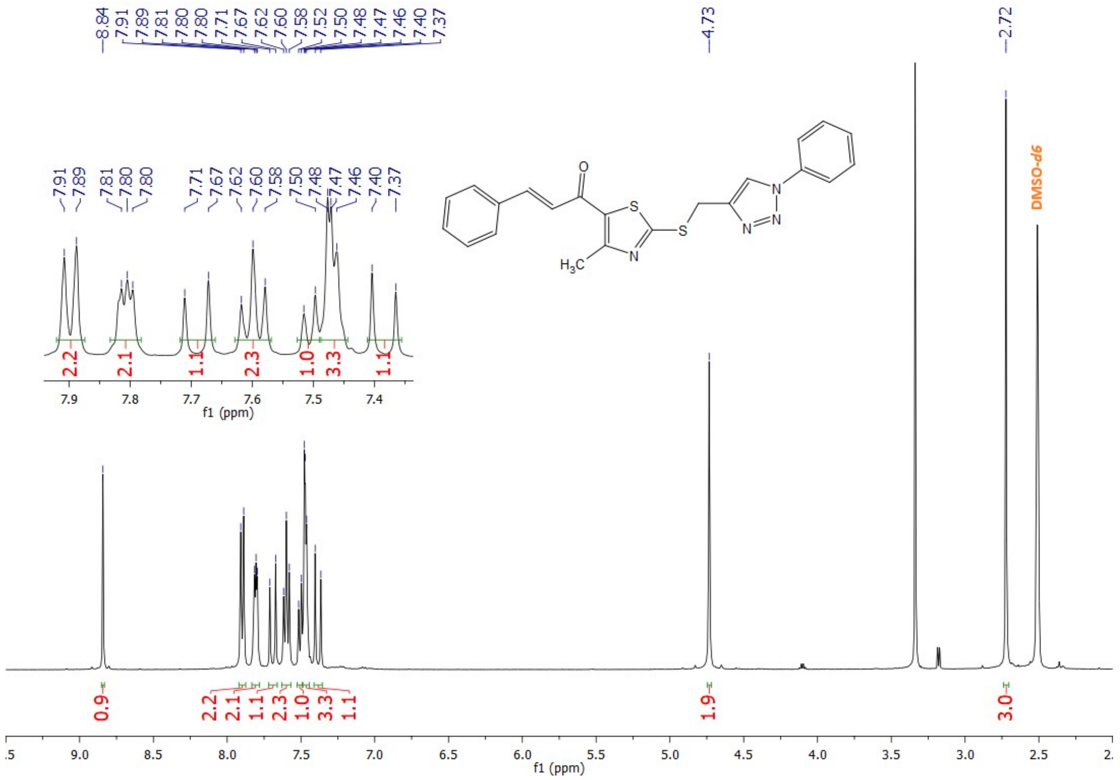


**Figure S11**. $^{1}$H NMR (400 MHz, DMSO-$d_6$) spectrum of **10a**

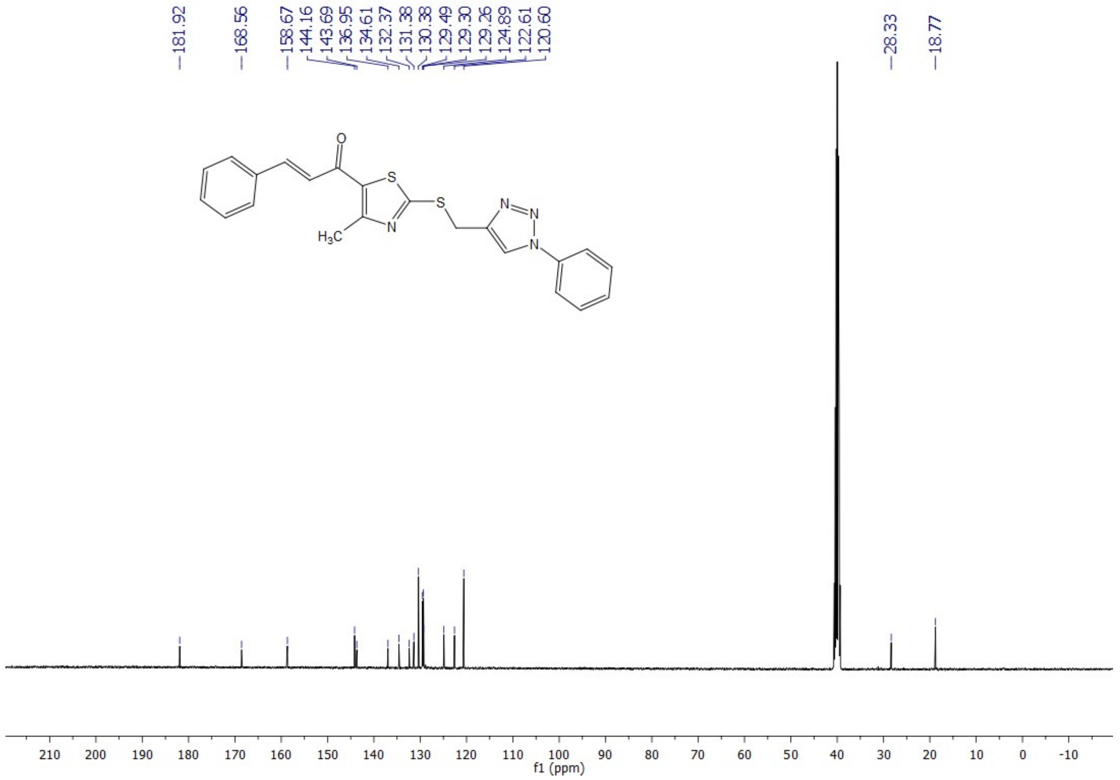


**Figure S12**. $^{13}$C NMR (100 MHz, DMSO-$d_6$) spectrum of **10a**

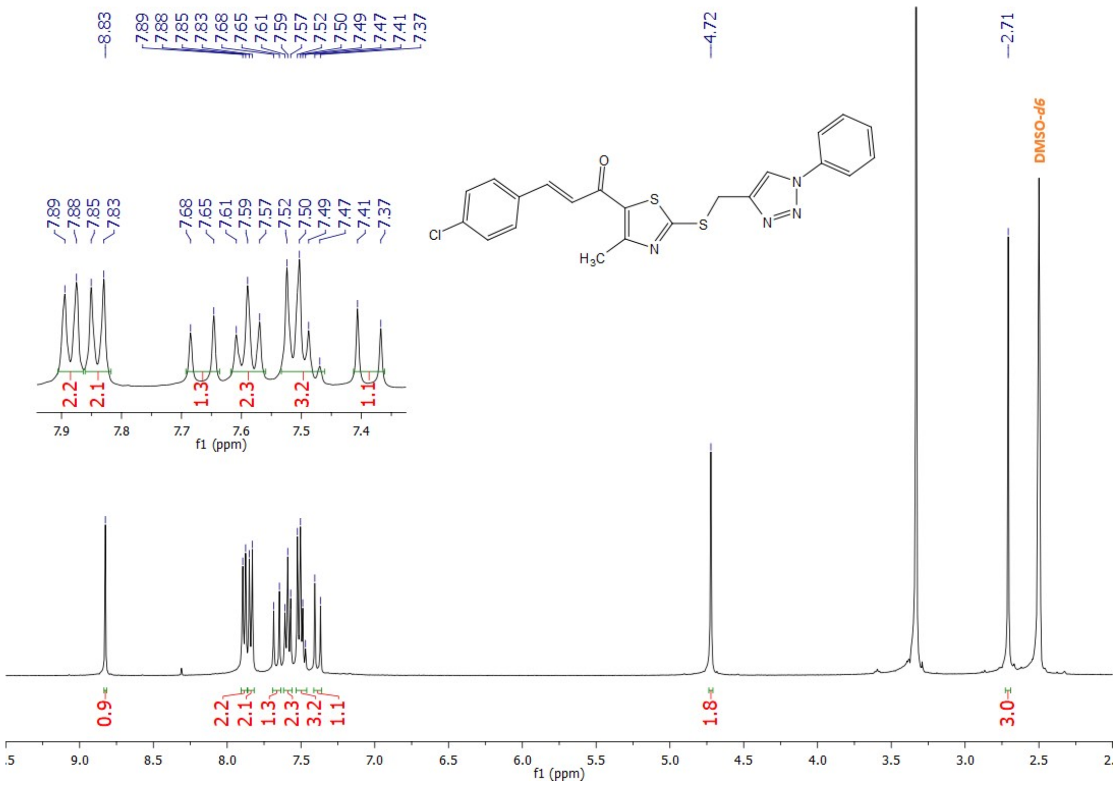


**Figure S13**. $^{1}$H NMR (400 MHz, DMSO-$d_6$) spectrum of **10b**

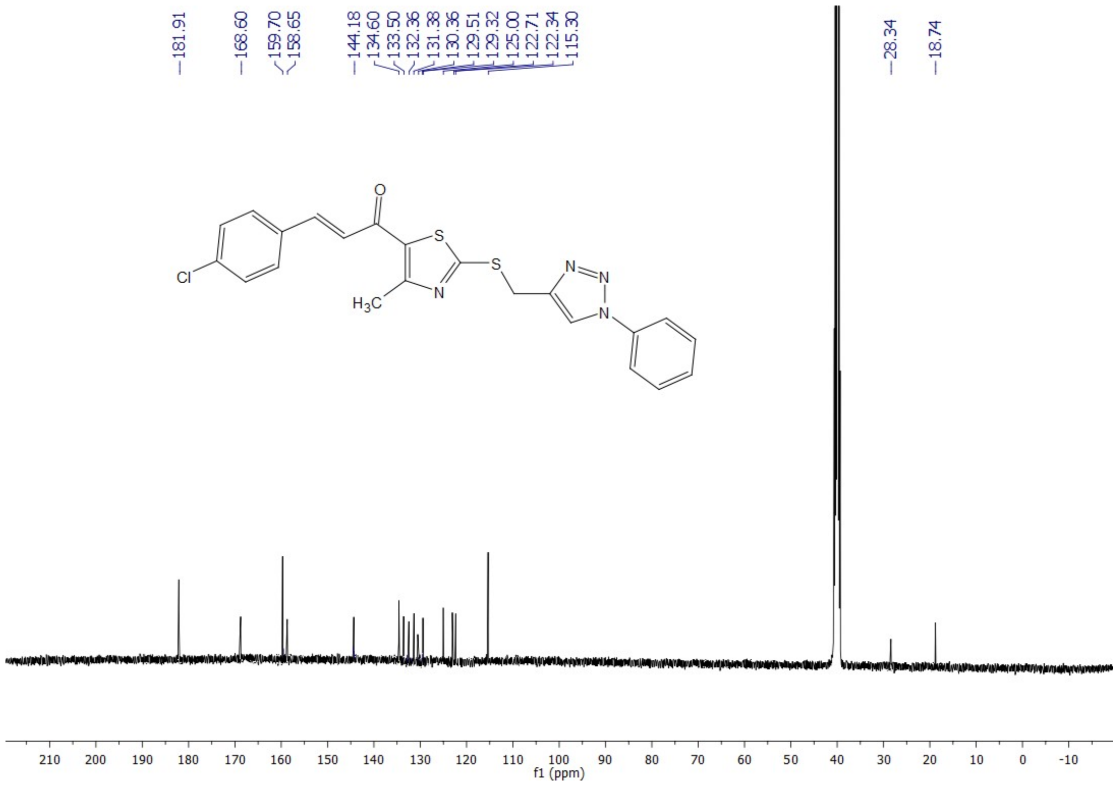


**Figure S14**. $^{13}$C NMR (100 MHz, DMSO-$d_6$) spectrum of **10b**

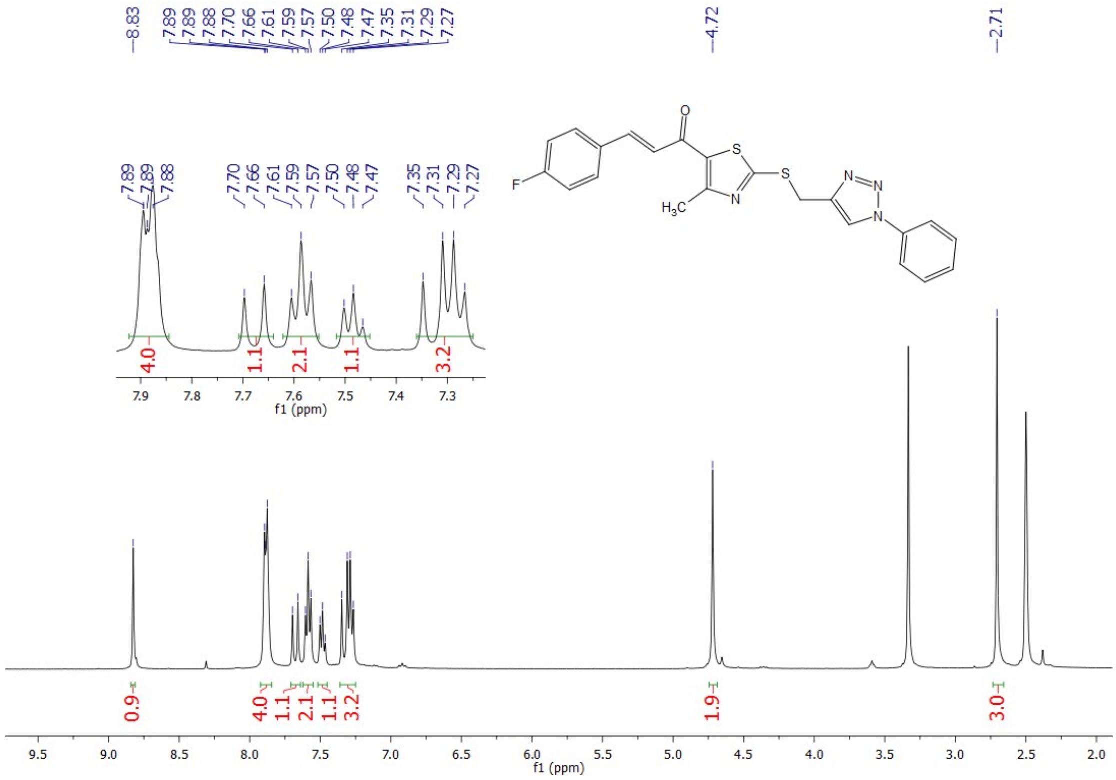


**Figure S15**. $^{1}$H NMR (400 MHz, DMSO-$d_6$) spectrum of **10c**

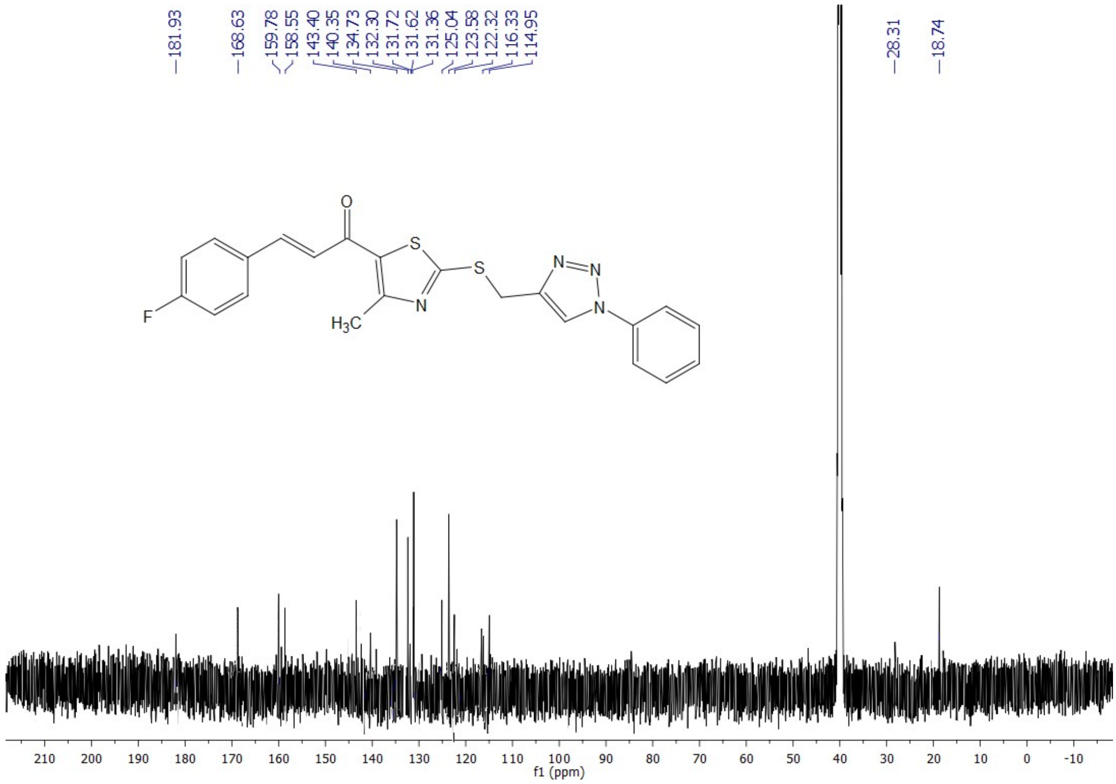


**Figure S16**. $^{13}$C NMR (100 MHz, DMSO-$d_6$) spectrum of **10c**

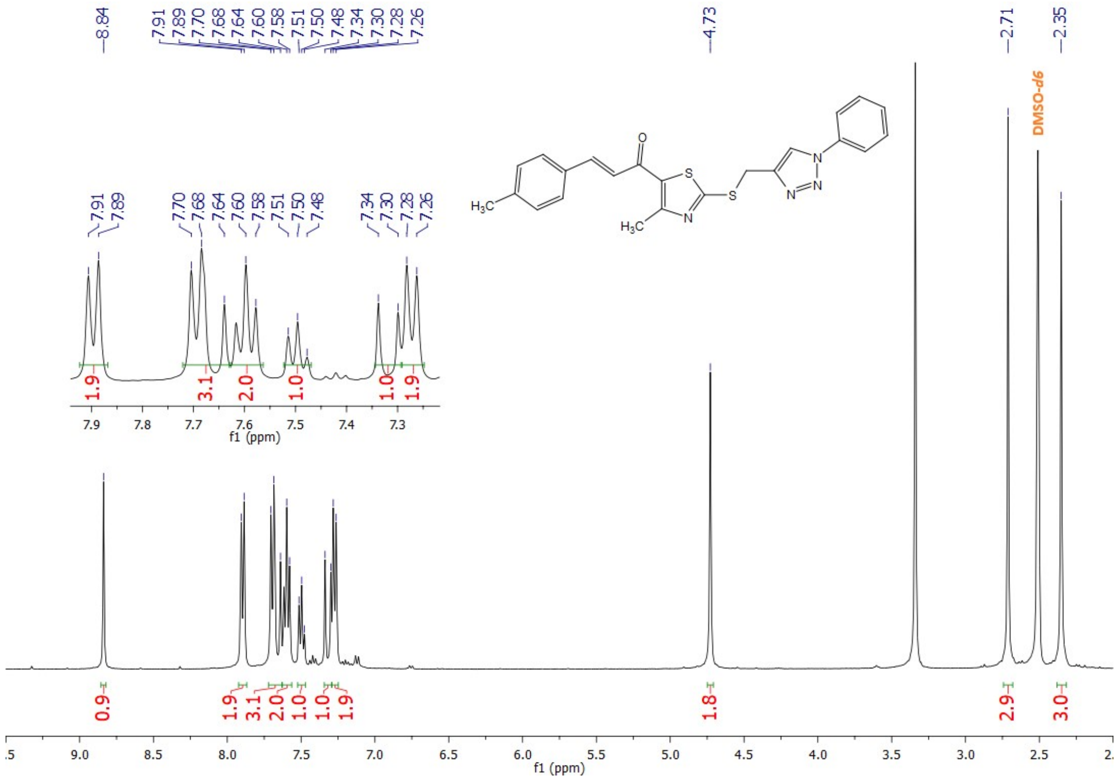

**Figure S17**. $^{1}$H NMR (400 MHz, DMSO-$d_6$) spectrum of **10d**

z

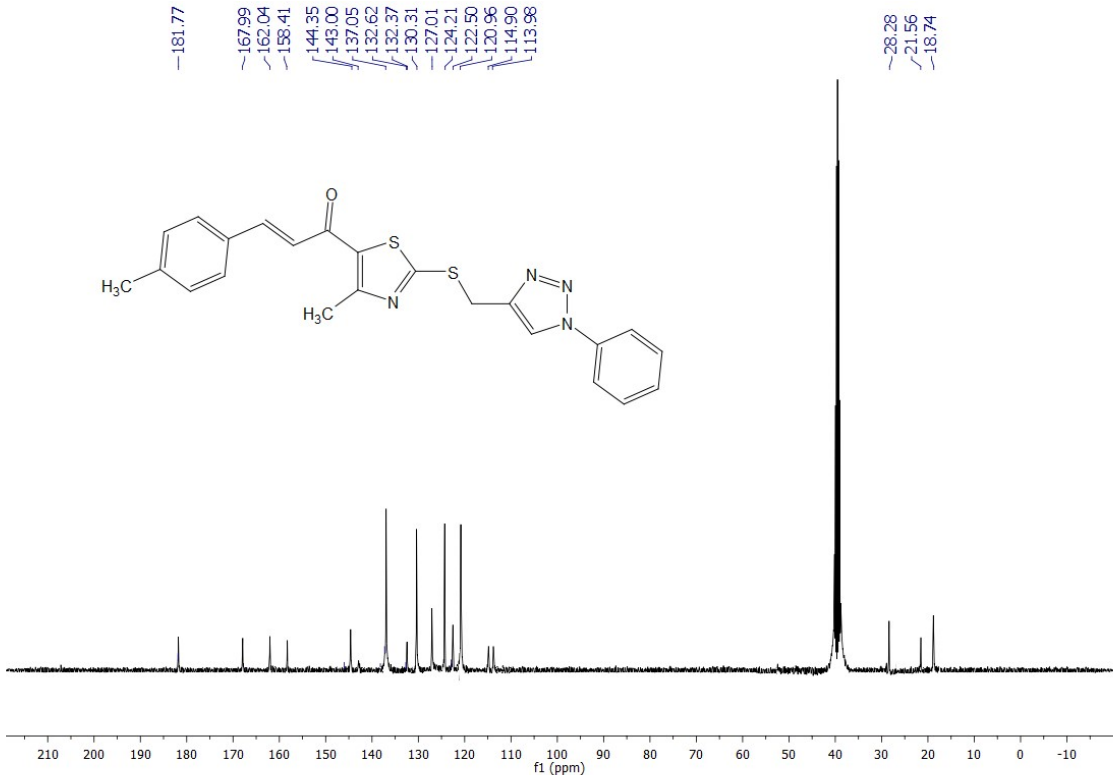

**Figure S18**. $^{13}$C NMR (100 MHz, DMSO-$d_6$) spectrum of **10d**

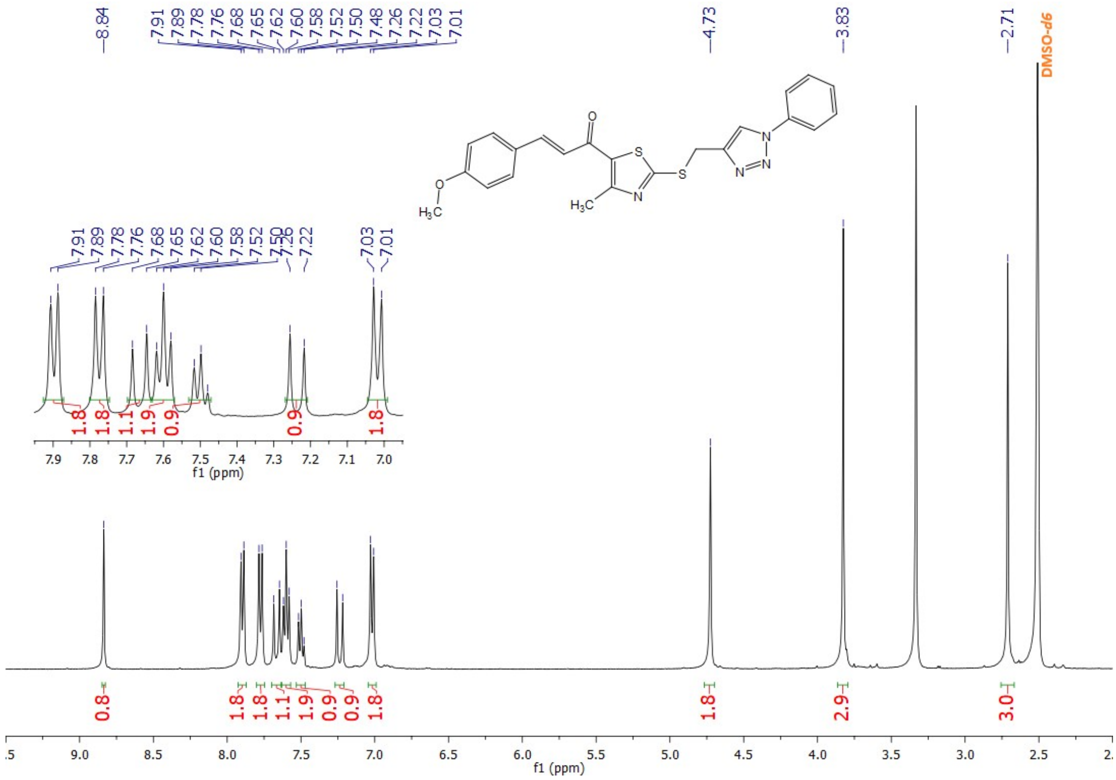


**Figure S19**. $^{1}$H NMR (400 MHz, DMSO-$d_6$) spectrum of **10e**

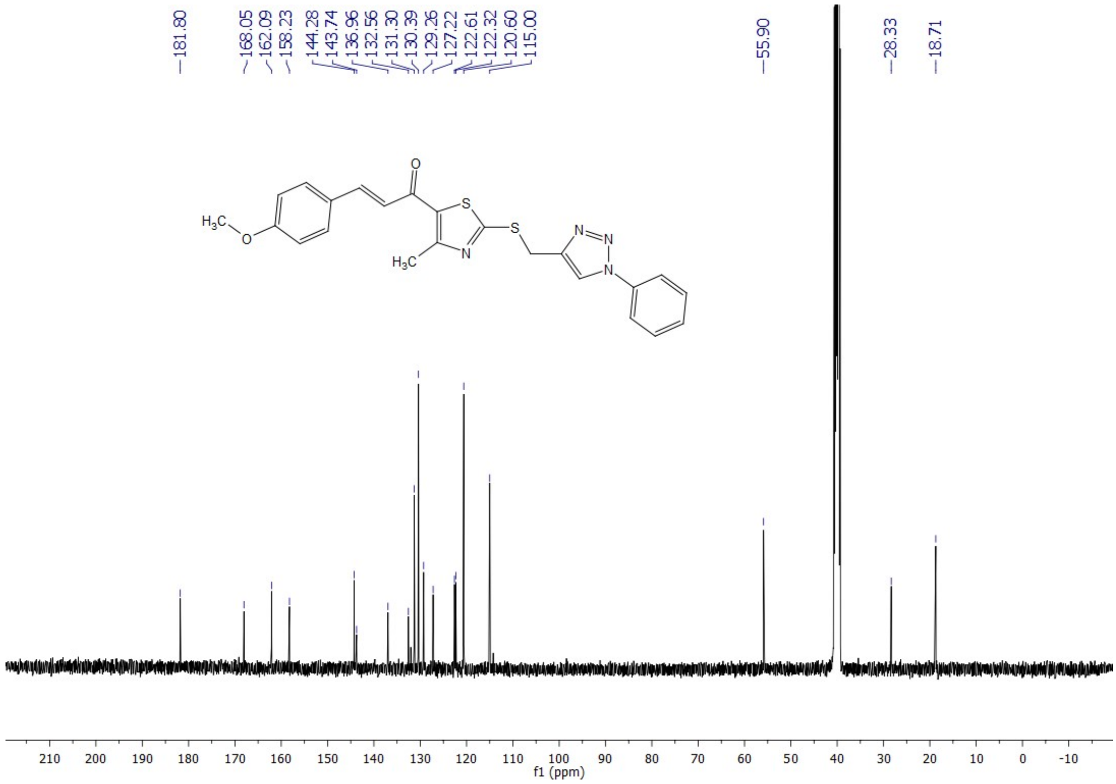


**Figure S20**. $^{13}$C NMR (100 MHz, DMSO-$d_6$) spectrum of **10e**

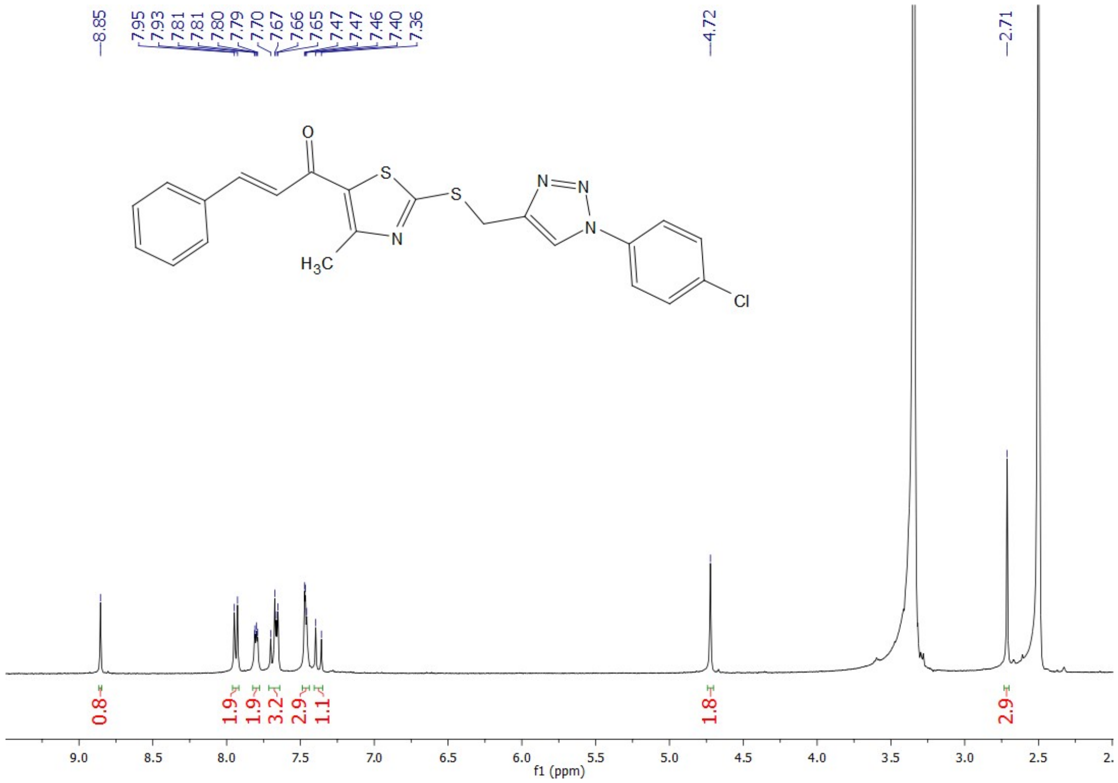


**Figure S21**. $^{1}H$ NMR (400 MHz, DMSO-$d_6$) spectrum of **10f**

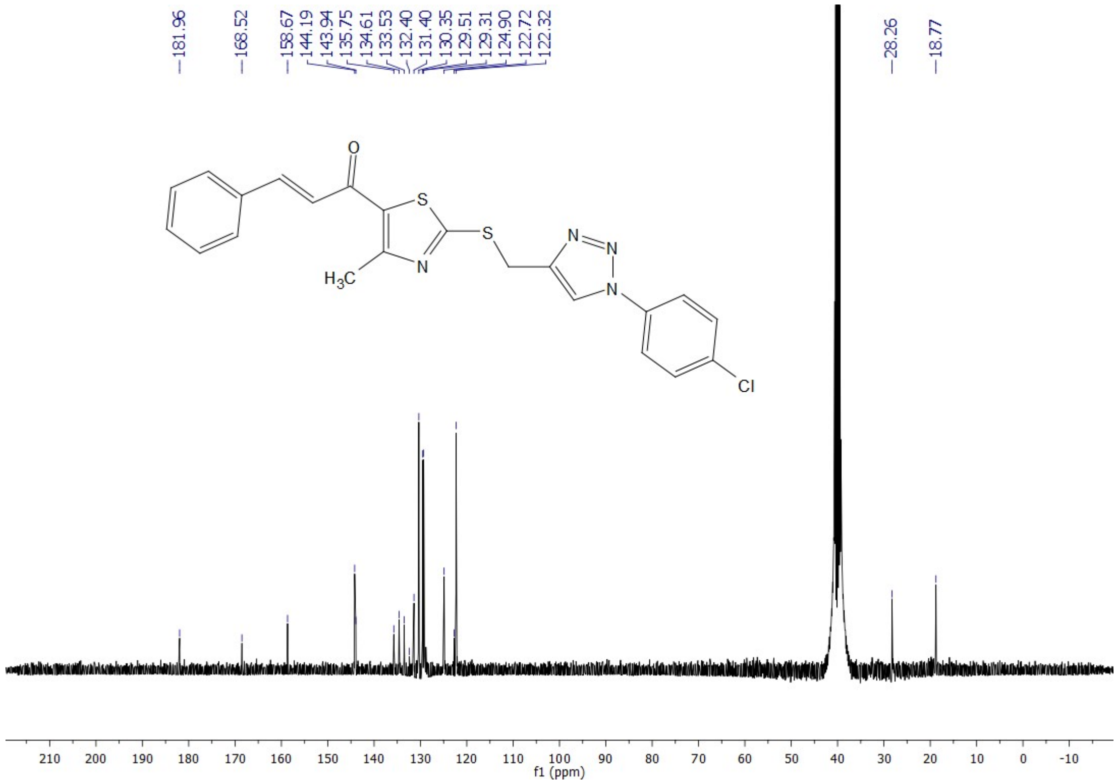


**Figure S22**. $^{13}C$ NMR (100 MHz, DMSO-$d_6$) spectrum of **10f**

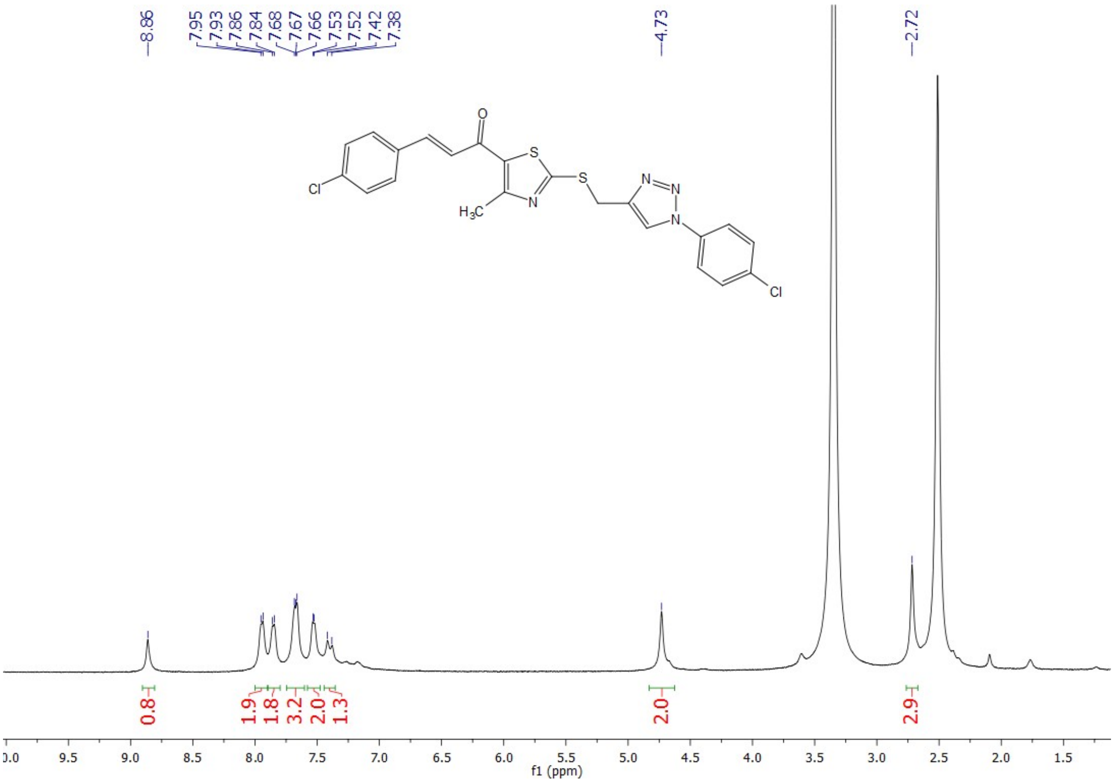


**Figure S23**. $^{1}$H NMR (400 MHz, DMSO-$d_6$) spectrum of **10g**

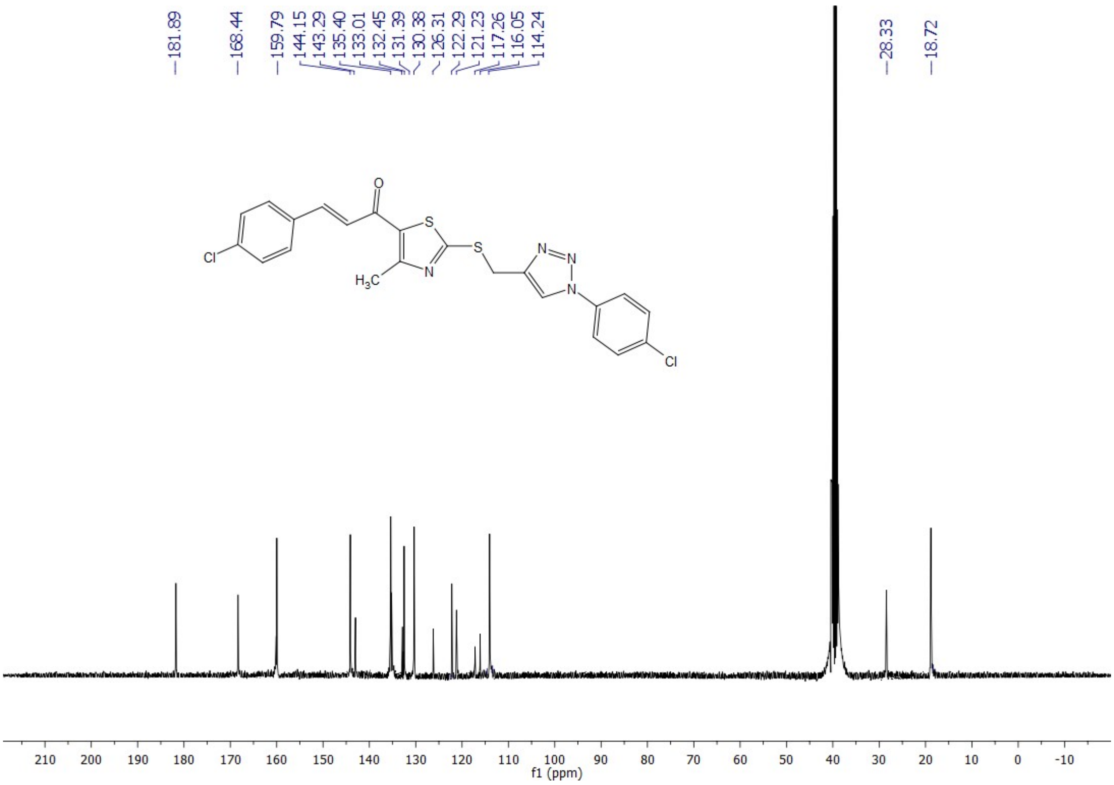


**Figure S24**. $^{13}$C NMR (100 MHz, DMSO-$d_6$) spectrum of **10g**

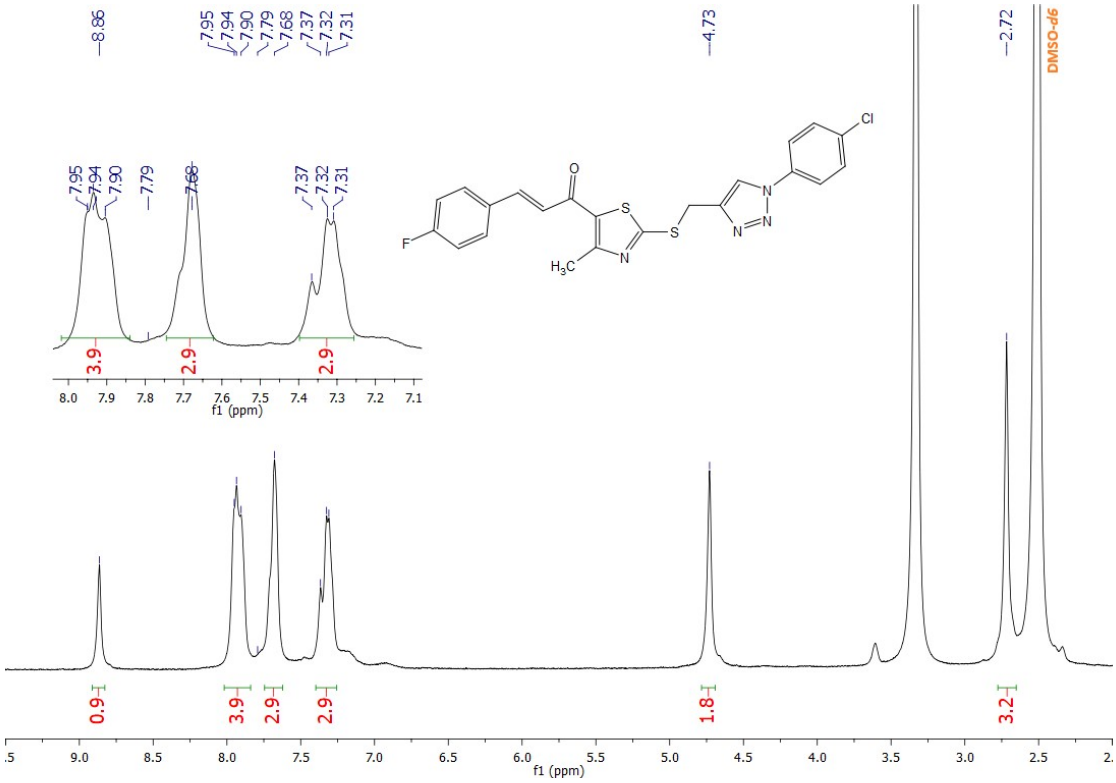


**Figure S25**. $^{1}$H NMR (400 MHz, DMSO-$d_6$) spectrum of **10h**

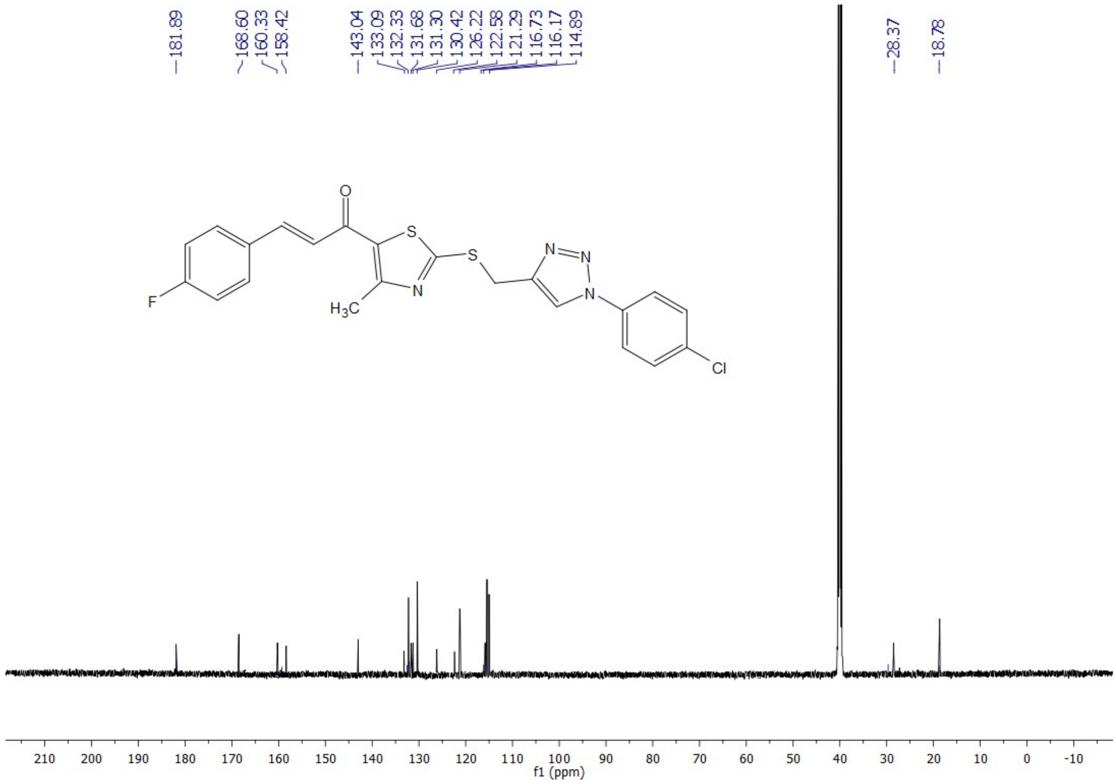


**Figure S26**. $^{13}$C NMR (100 MHz, DMSO-$d_6$) spectrum of **10h**

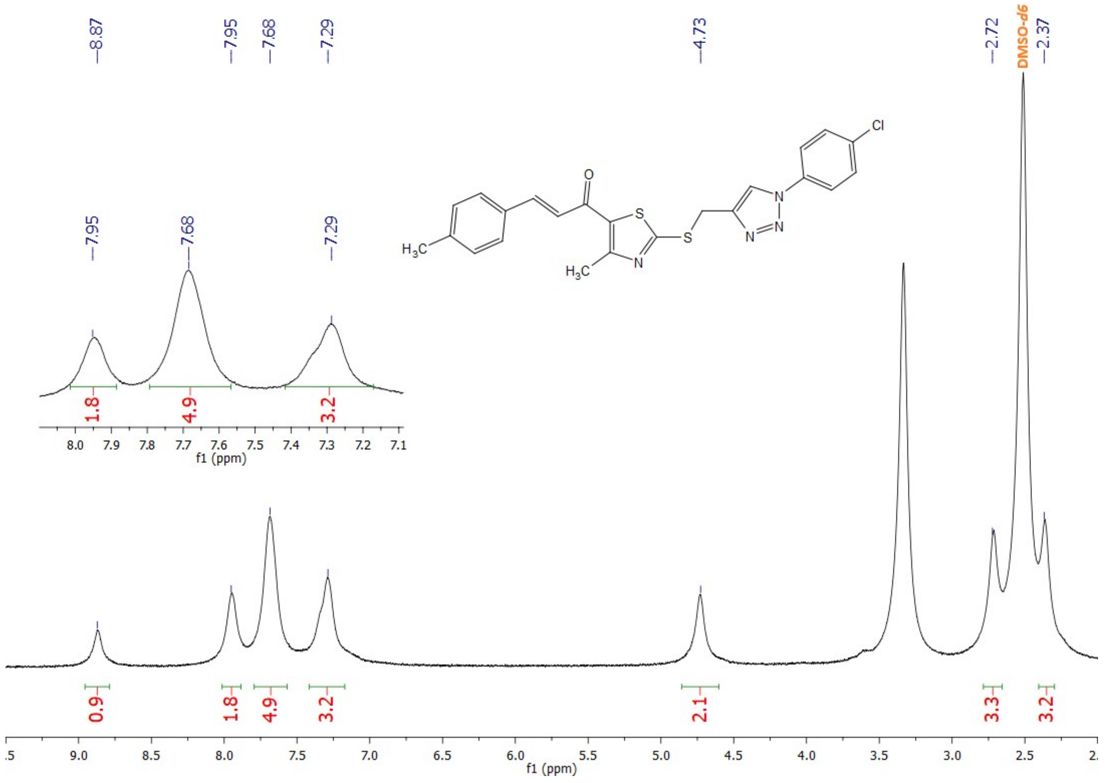


**Figure S27**. $^{1}$H NMR (400 MHz, DMSO-$d_6$) spectrum of **10i**

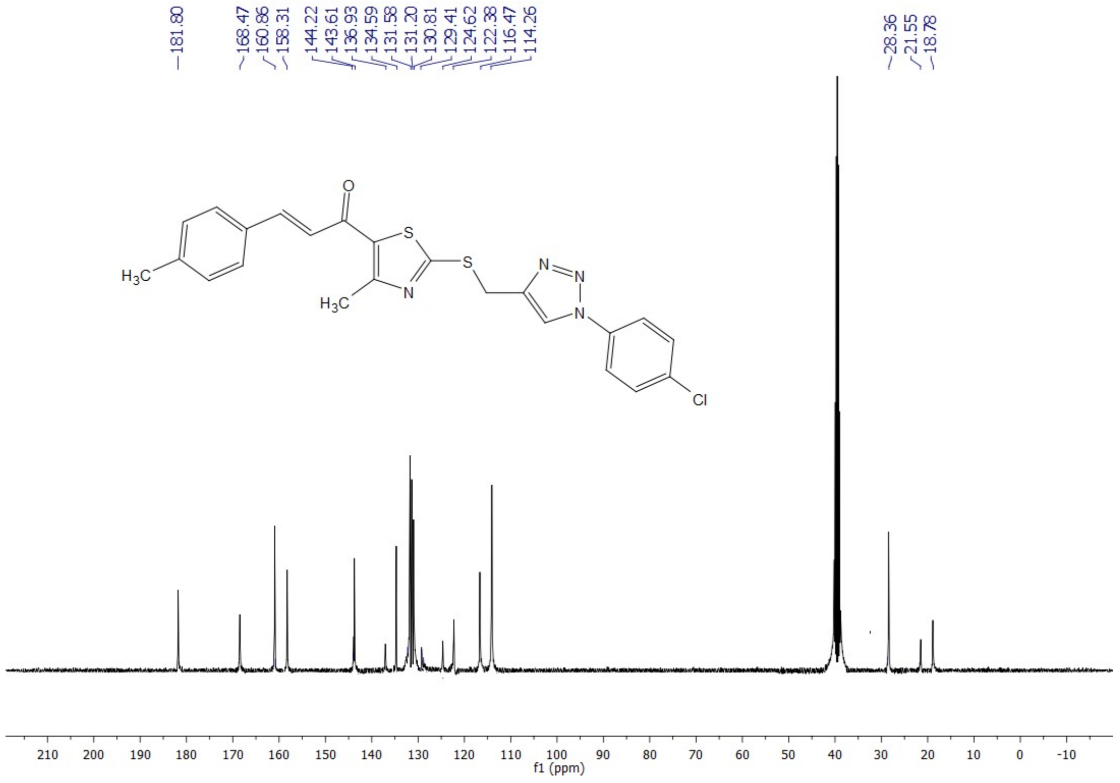


**Figure S28**. $^{13}$C NMR (100 MHz, DMSO-$d_6$) spectrum of **10i**

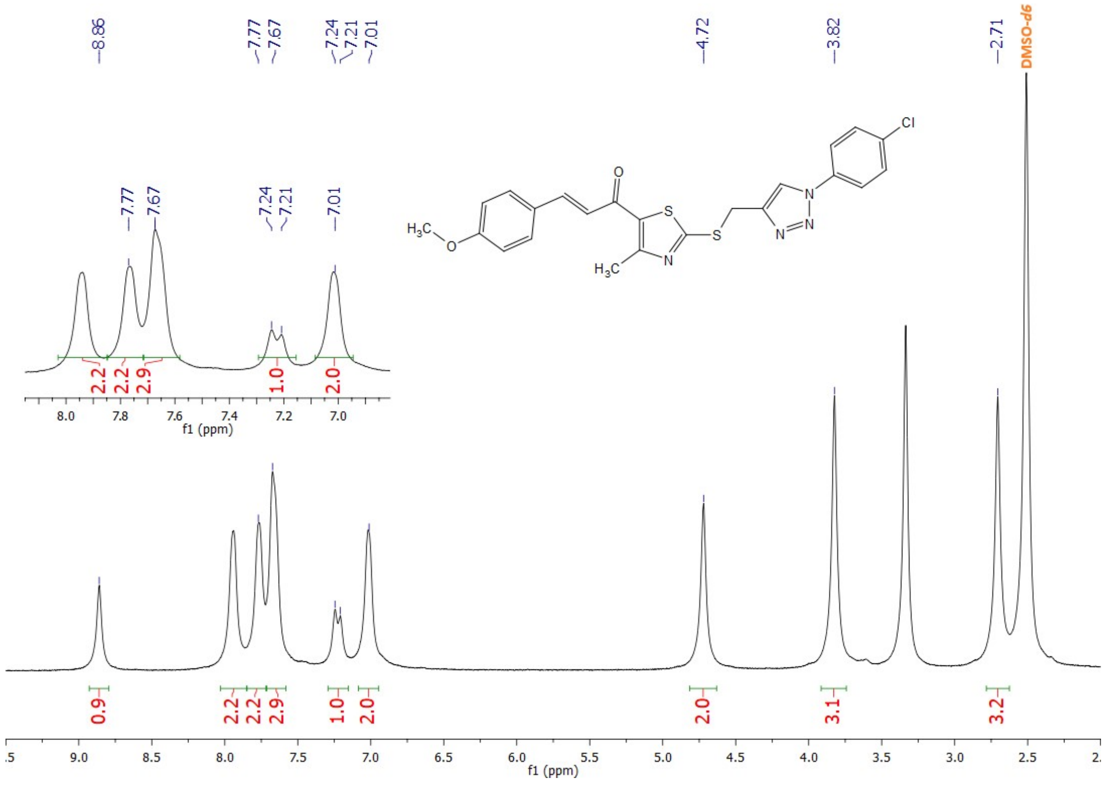

**Figure S29**. $^{1}$H NMR (400 MHz, DMSO-$d_6$) spectrum of **10j**

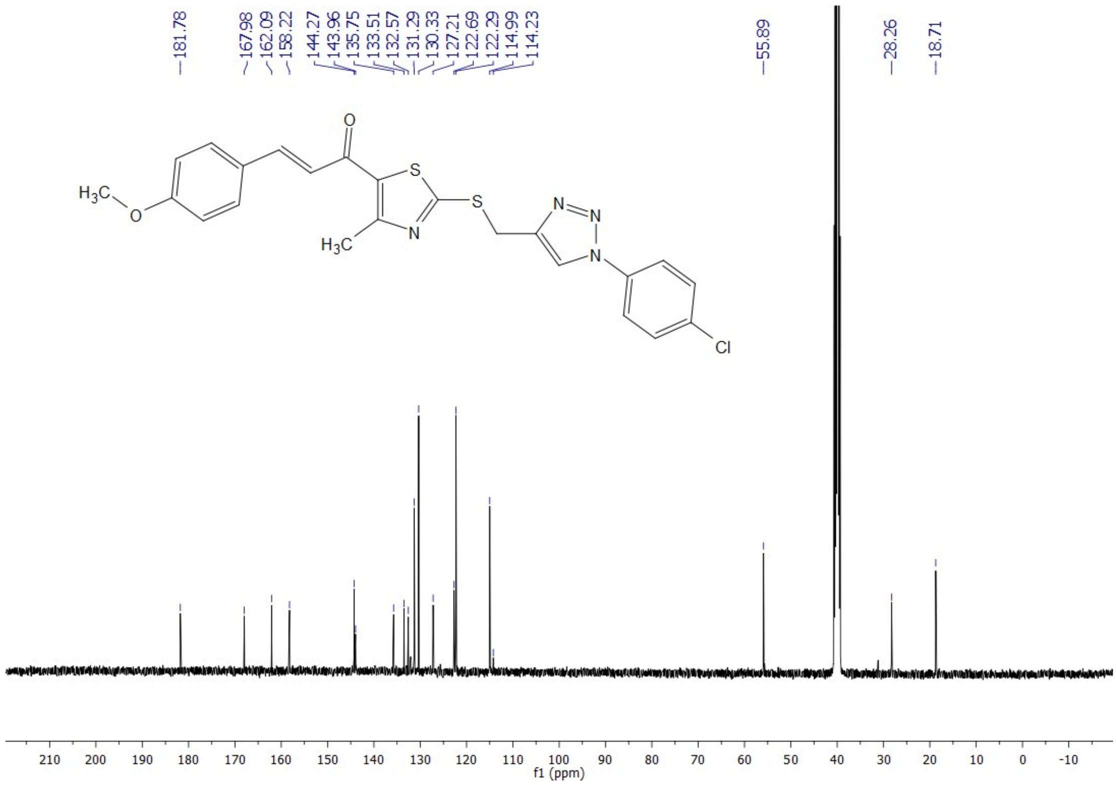

**Figure S30**. $^{13}$C NMR (100 MHz, DMSO-$d_6$) spectrum of **10j**

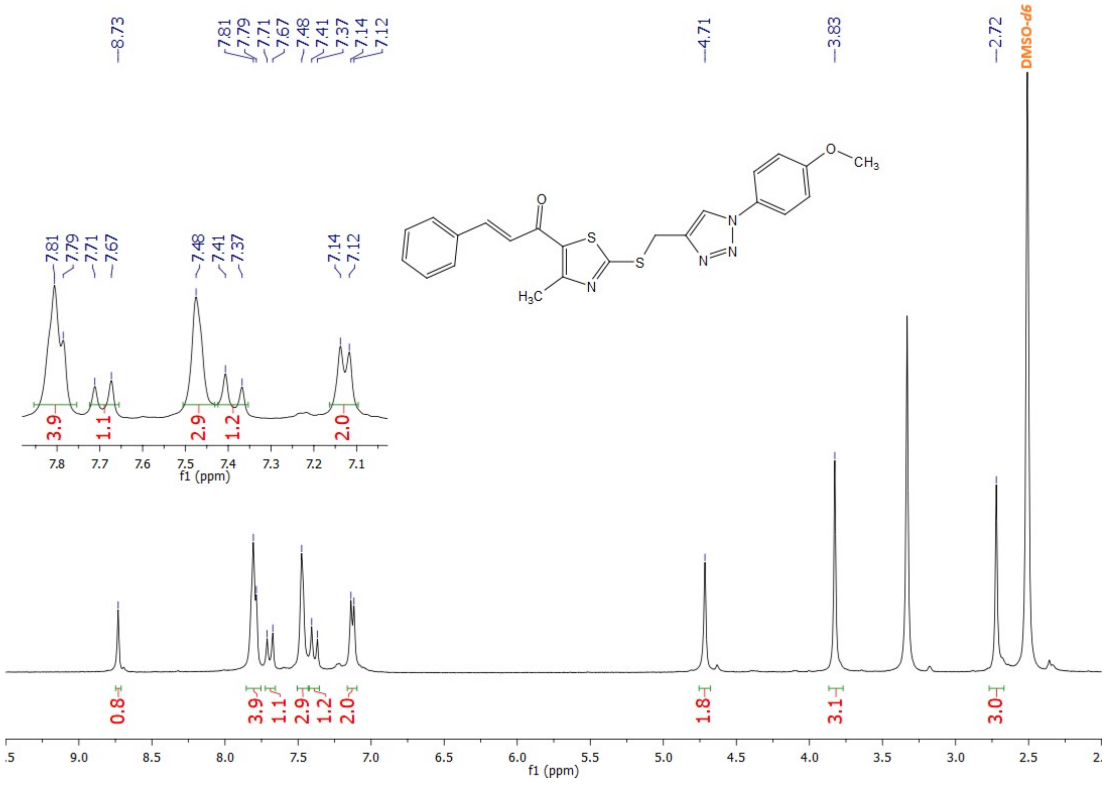

**Figure S31**. $^{1}$H NMR (400 MHz, DMSO-$d_6$) spectrum of **10k**

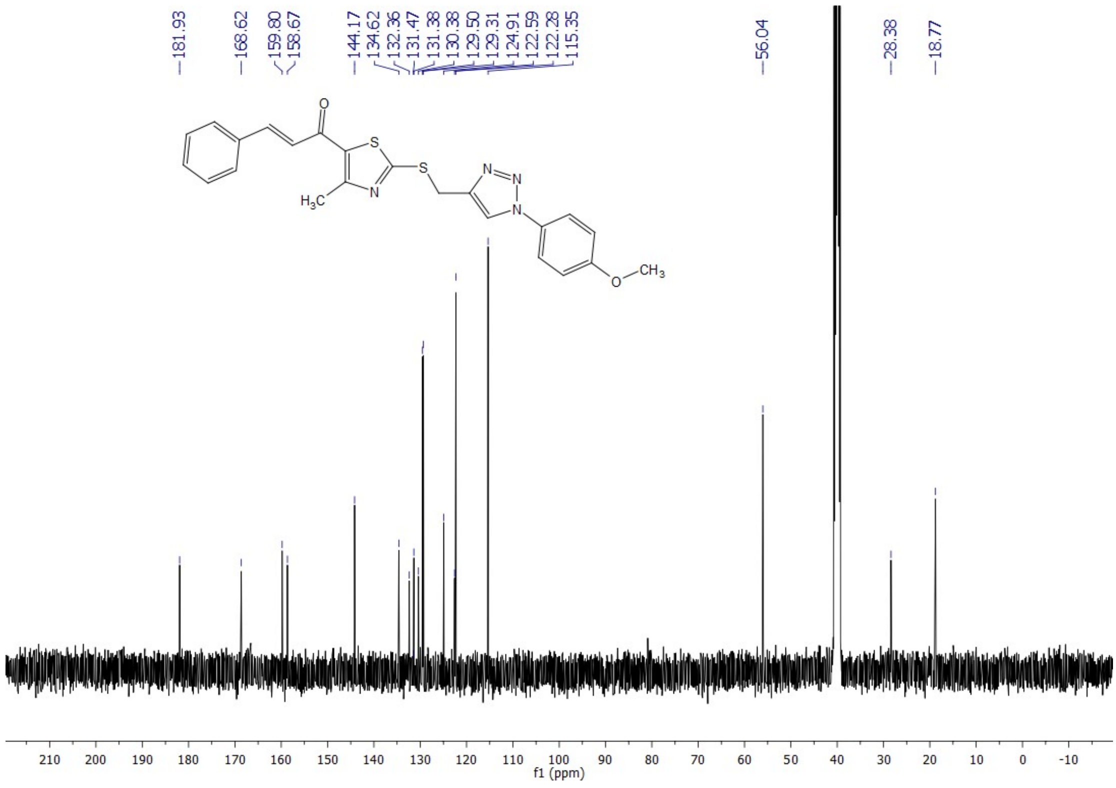

**Figure S32**. $^{13}$C NMR (100 MHz, DMSO-$d_6$) spectrum of **10k**

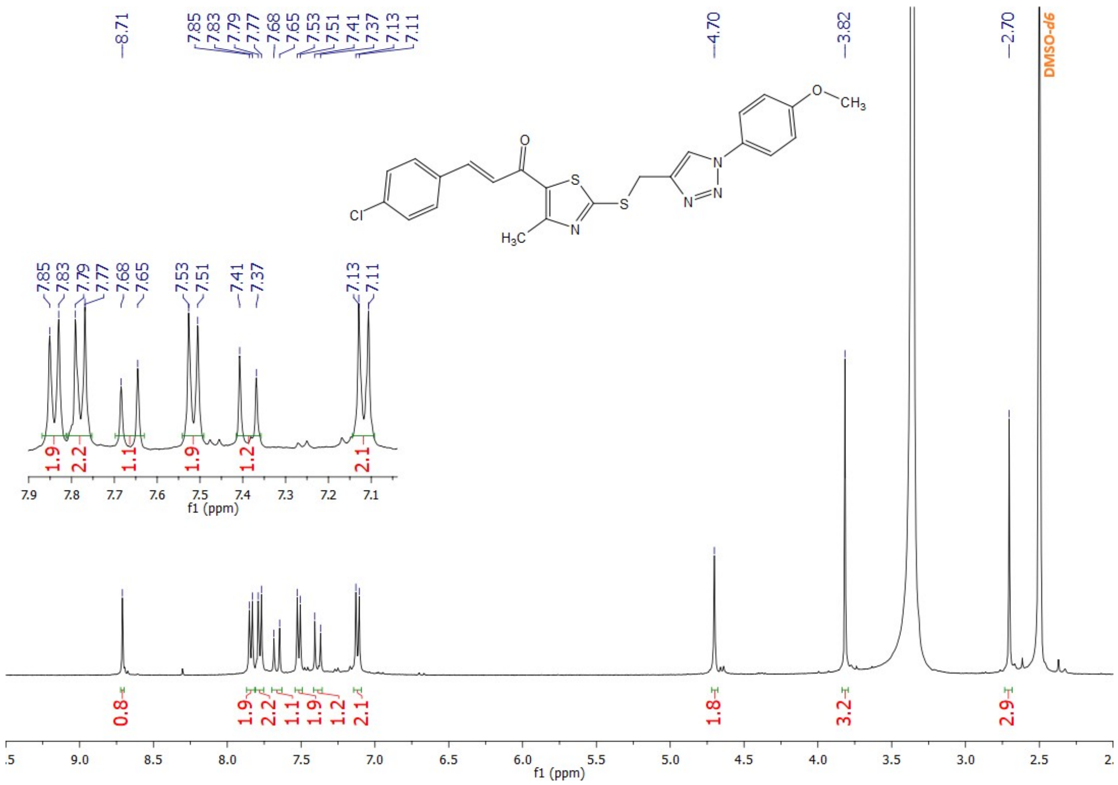

**Figure S33**. $^{1}$H NMR (400 MHz, DMSO-$d_6$) spectrum of **10l**

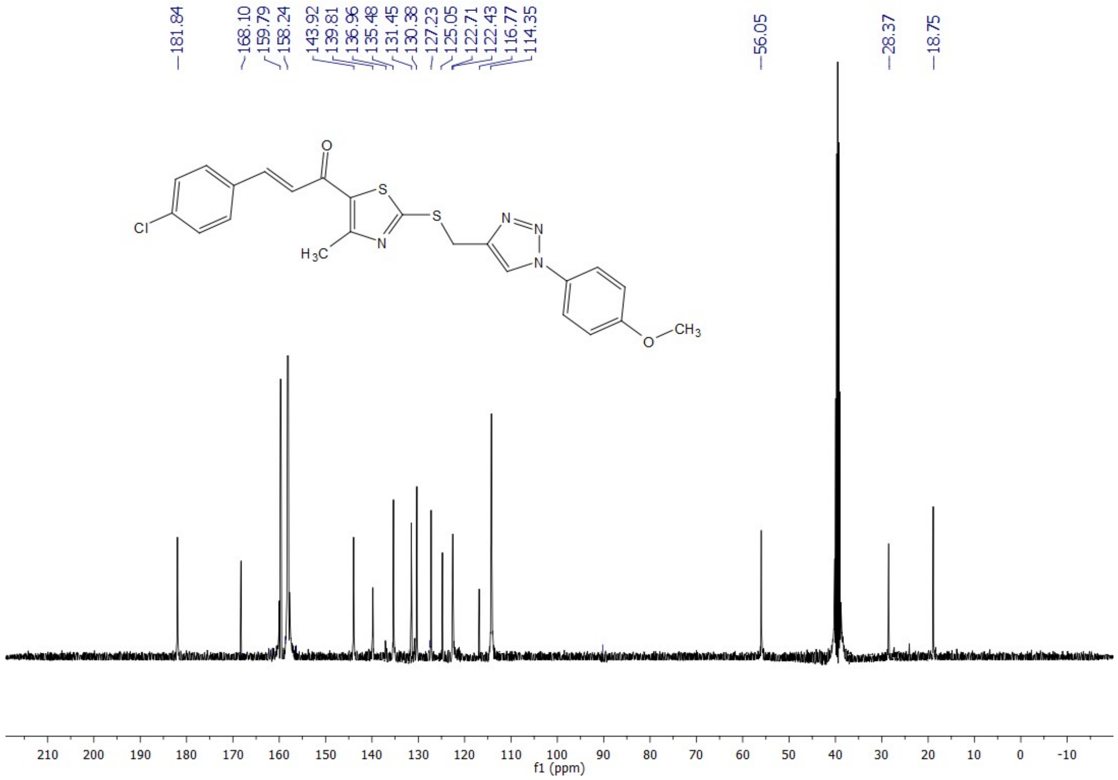

**Figure S34**. $^{13}$C NMR (100 MHz, DMSO-$d_6$) spectrum of **10l**

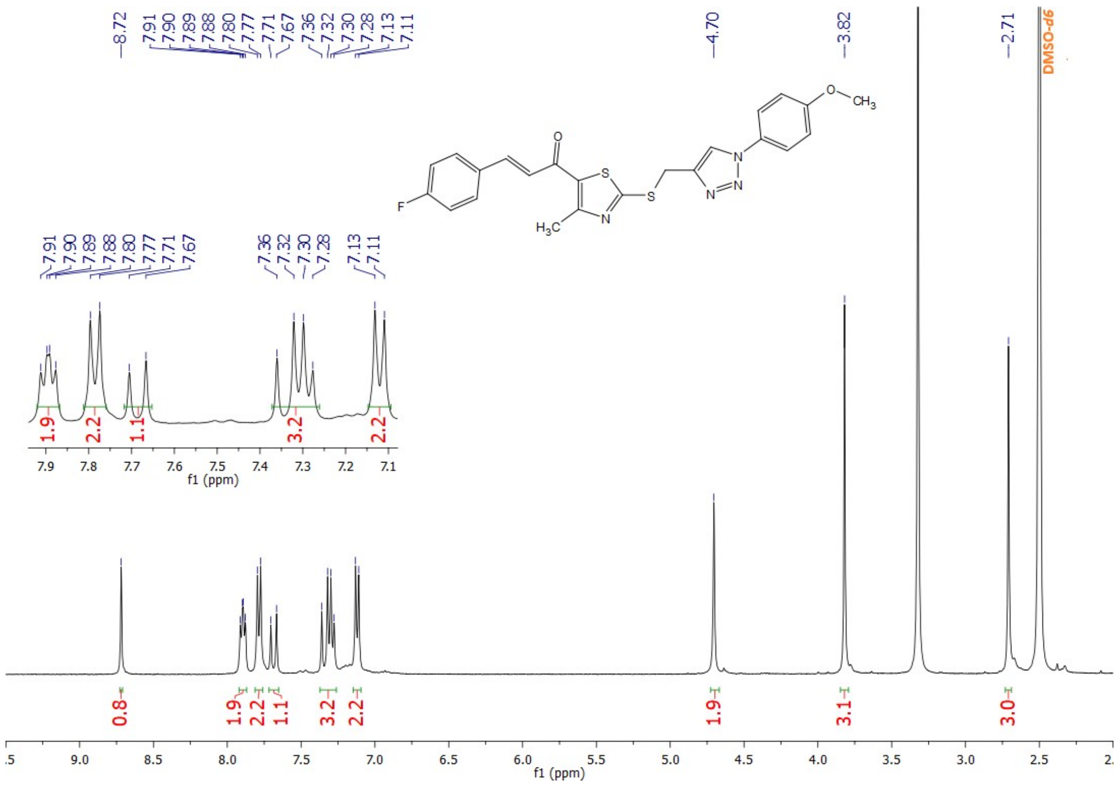


**Figure S35**. $^{1}$H NMR (400 MHz, DMSO-$d_6$) spectrum of **10m**

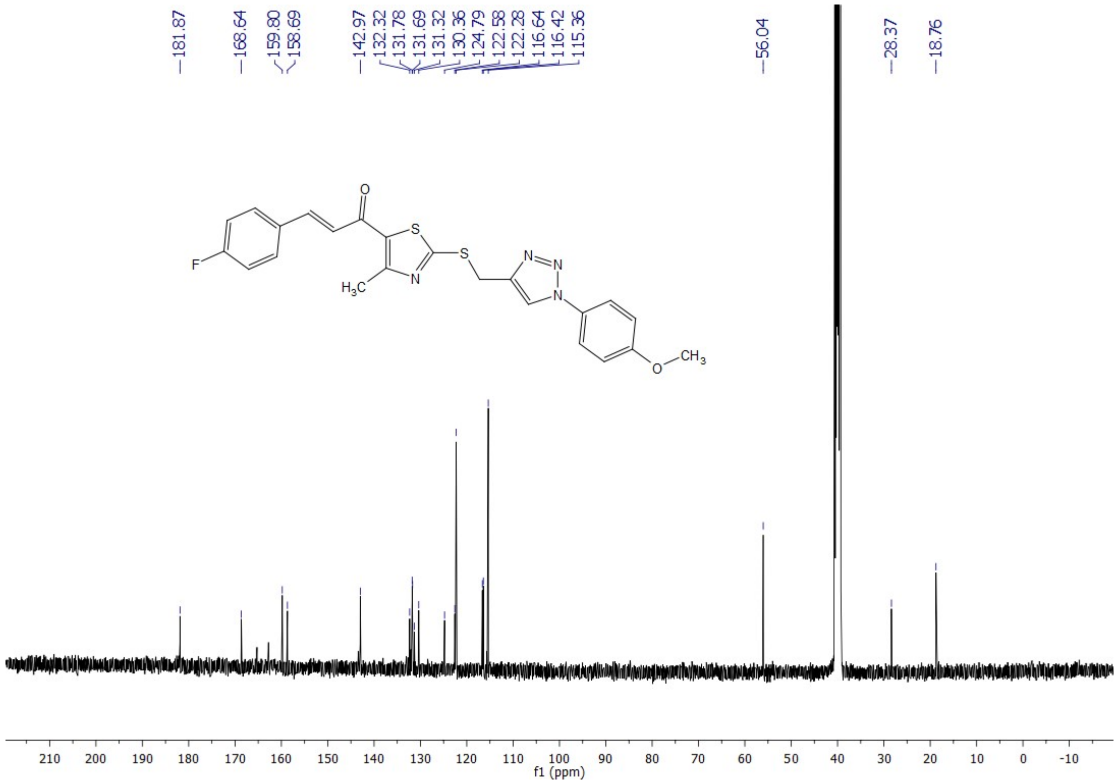


**Figure S36**. $^{13}$C NMR (100 MHz, DMSO-$d_6$) spectrum of **10m**

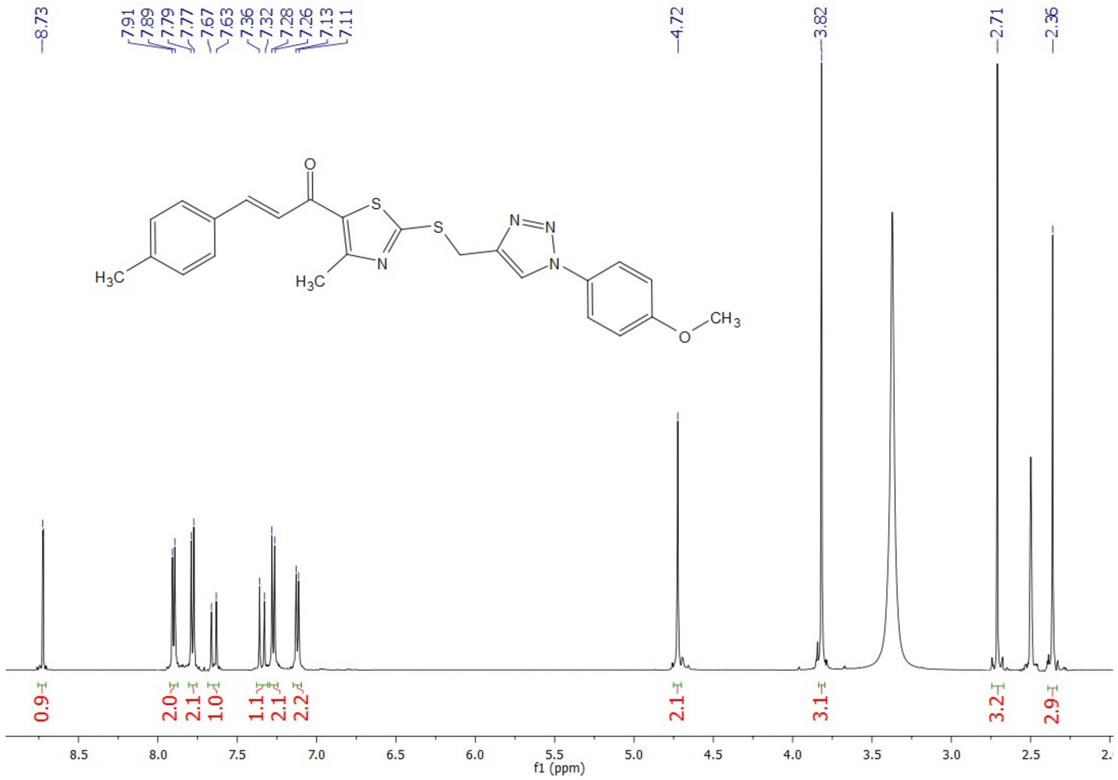


**Figure S37**. $^{1}H$ NMR (400 MHz, DMSO-$d_6$) spectrum of **10n**

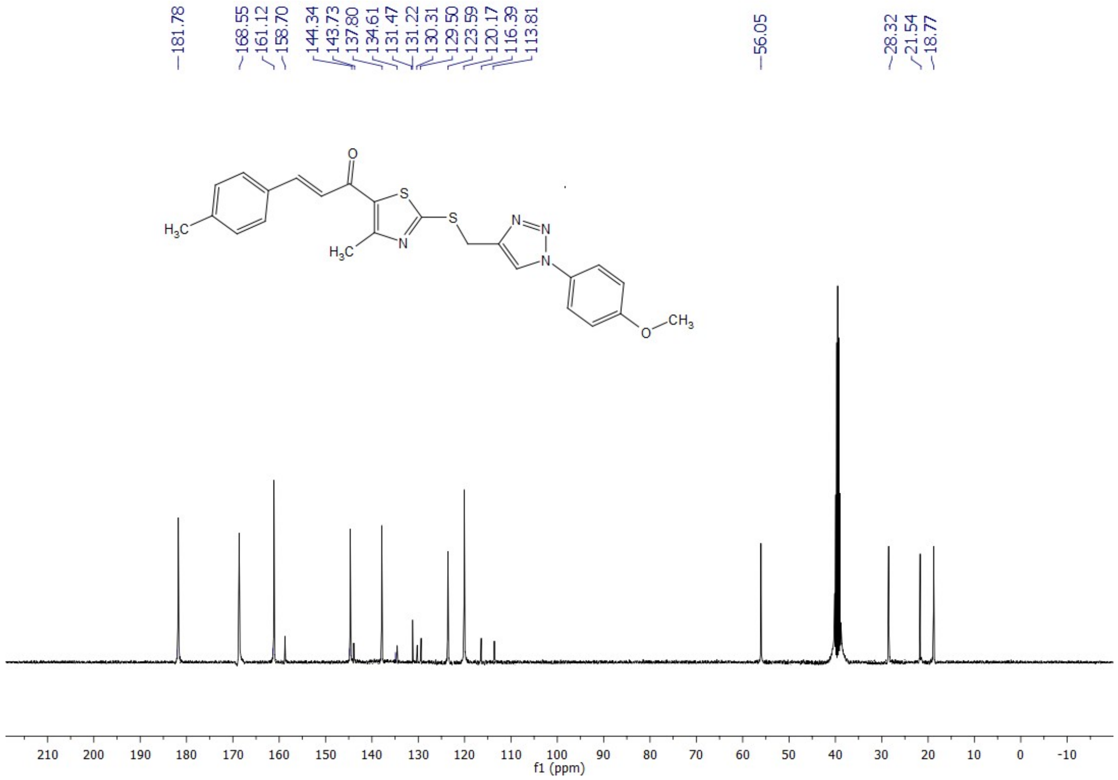


**Figure S38**. $^{13}C$ NMR (100 MHz, DMSO-$d_6$) spectrum of **10n**

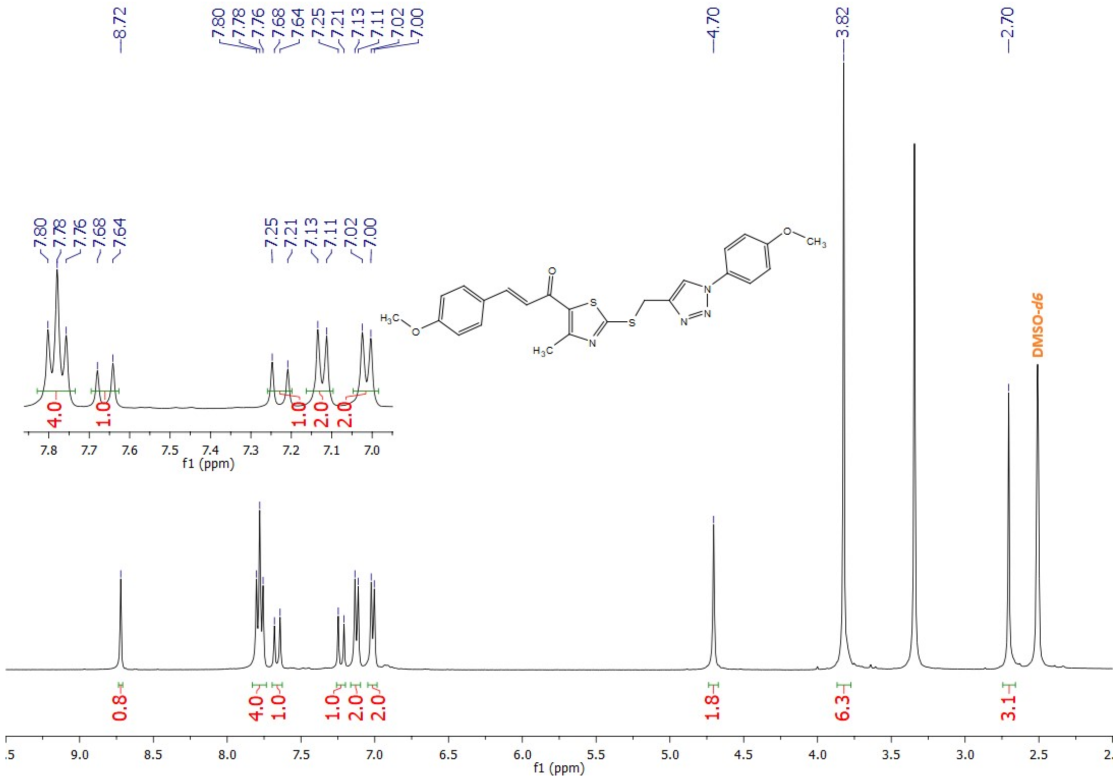

**Figure S39**. $^{1}$H NMR (400 MHz, DMSO-$d_6$) spectrum of **10o**

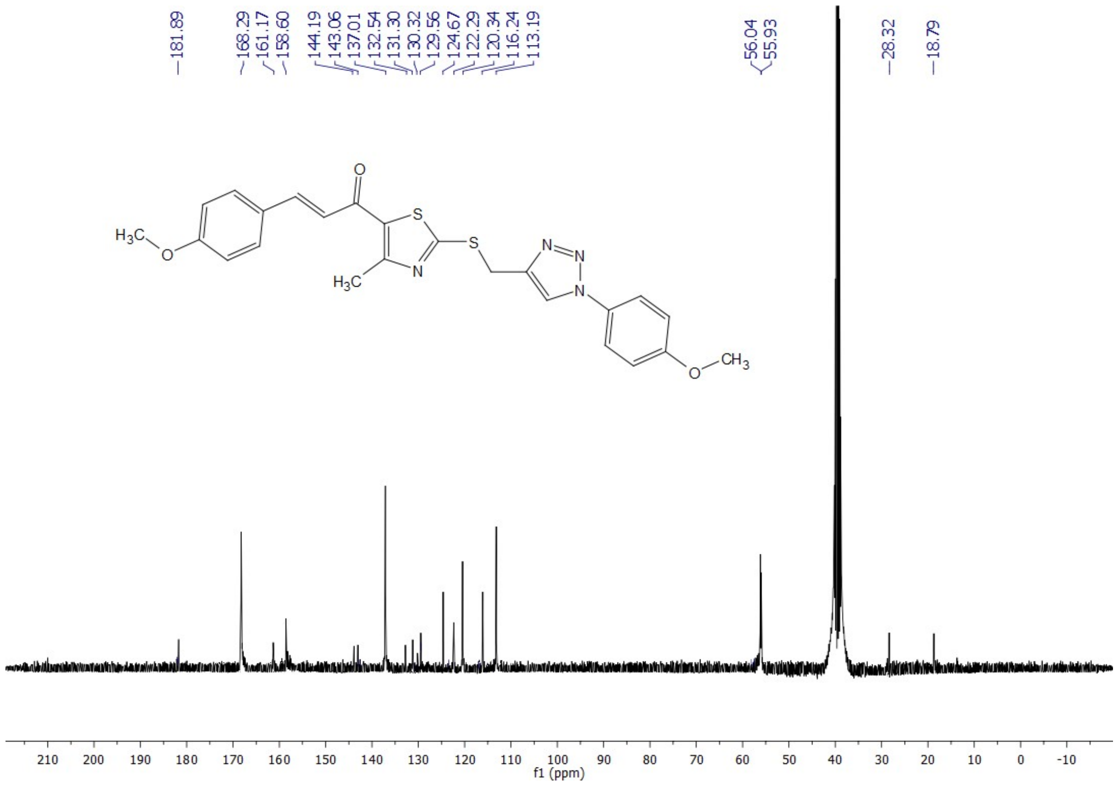

**Figure S40**. $^{13}$C NMR (100 MHz, DMSO-$d_6$) spectrum of **10o**

# Appendix A

## 4. EXPERIMENTAL

### 4.1. Chemistry

**Materials and methods**

All reagents and solvents were of general purpose or analytical grade and purchased from Sigma Aldrich Ltd, Fisher Scientific, Fluka and Acros. 1H and 13C NMR spectra were recorded with a Bruker Avance III spectrometer operating at 400, 100 MHz respectively, with Me4Si as internal standard and DMSO-d6 as a solvent. Elemental analysis was performed by the regional center for mycology and biotechnology (Cairo, Egypt). TLC was carried out on precoated silica plates (Keisel gel 60 F254, BDH) using Hexane: Ethyl acetate, 1 : 2, v/v. Compounds were visualized by illumination under UV light (254 nm). Melting points were determined on an electrothermal instrument and are uncorrected. All solvents were dried prior to use and stored over 4 Å molecular sieves, under nitrogen. All the compounds were ≥ 95% pure.

### 4.2. Biological evaluation

#### 4.2.1 Cell Viability assay (MTT assay)

MTT assay was performed to investigate the effect of the synthesized compounds on mammary epithelial cells (MCF-10A). The cells were propagated in medium consisting of Ham's F-12 medium/ Dulbecco's modified Eagle's medium (DMEM) (1:1) supplemented with 10% foetal calf serum, 2 mM glutamine, insulin (10 µg/mL), hydrocortisone (500 ng/mL) and epidermal growth factor (20 ng/mL). Trypsin ethylenediamine tetra acetic acid (EDTA) was used to passage the cells after every 2-3 days. 96-well flat-bottomed cell culture plates were used to seed the cells at a density of 104 cells $mL^{-1}$. The medium was aspirated from all the wells of culture plates after 24 h followed by the addition of synthesized compounds (in 200 µL medium to yield a

final concentration of 0.1% (v/v) dimethyl sulfoxide) into individual wells of the plates. Four wells were designated to a single compound. The plates were allowed to incubate at 37°C for 96 h. Afterwards, the medium was aspirated and 3-[4,5-dimethylthiazol-2-yl]-2,5-diphenyltetrazolium bromide (MTT) (0.4 mg/mL) in medium was added to each well and subsequently incubated for 3 h. The medium was aspirated and 150 μL dimethyl sulfoxide (DMSO) was added to each well. The plates were vortexed followed by the measurement of absorbance at 540 nm on a microplate reader. The results were presented as inhibition (%) of proliferation in contrast to controls comprising 0.1% DMSO.

**4.2.2. Assay for antiproliferative effect**

To explore the antiproliferative potential of compounds propidium iodide fluorescence assay was performed using different cell lines such as Panc-1 (pancreas cancer cell line), MCF-7 (breast cancer cell line), HT-29 (colon cancer cell line) and A-549 (epithelial cancer cell line), respectively. All cell lines were obtained from ATCC (American Type Cell Culture) via Holding company for biological products and vaccines (VACSERA), Cairo, Egypt. To calculate the total nuclear DNA, a fluorescent dye (propidium iodide, PI) is used which can attach to the DNA, thus offering a quick and precise technique. PI cannot pass through the cell membrane, and its signal intensity can be considered as directly proportional to quantity of cellular DNA. Cells whose cell membranes are damaged or have changed permeability are counted as dead ones. The assay was performed by seeding the cells of different cell lines at a density of 3000-7500 cells/well (in200μl medium) in culture plates followed by incubation for 24h at 37 °C in humidified 5%CO2/95% air atmospheric conditions. The medium was removed; the compounds were added to the plates at 10 μM concentrations (in 0.1% DMSO) in triplicates, followed by incubation for 48h. DMSO (0.1%) was used as

control. After incubation, medium was removed followed by the addition of PI (25 µl, 50µg/mL in water/medium) to each well of the plates. At -80 °C, the plates were allowed to freeze for 24 h, followed by thawing at 25 °C. A fluorometer (Polar-Star BMG Tech) was used to record the readings at excitation and emission wavelengths of 530 and 620 nm for each well. The percentage cytotoxicity of compounds was calculated using the following formula:

$$\% \text{ Cytotoxicity} = \frac{A_C - A_{TC}}{A_C} \times 100$$

Where A*TC*= Absorbance of treated cells and AC= Absorbance of control. Erlotinib was used as positive control in the assay.

**4.2.3. EGFR inhibitory assay**

Baculoviral expression vectors including pBlueBacHis2B and pFASTBacHTc were used separately to clone 1.6 kb cDNA coding for EGFR cytoplasmic domain (EGFR-CD, amino acids 645–1186). 5′ upstream to the EGFR sequence comprised a sequence that encoded (His)6. Sf-9 cells were infected for 72h for protein expression. The pellets of Sf-9 cells were solubilized in a buffer containing sodium vanadate (100 µM), aprotinin (10 µg/mL), triton (1%), HEPES buffer(50mM), ammonium molybdate (10 µM), benzamidine HCl (16 µg/mL), NaCl (10 mM),leupeptin (10 µg/mL) and pepstatin (10 µg/mL) at 0°C for 20 min at pH 7.4, followed by centrifugation for 20 min. To eliminate the nonspecifically bound material, a Ni-NTA super flow packed column was used to pass through and wash the crude extract supernatant first with 10mM and then with 100 mM imidazole. Histidine-linked proteins were first eluted with 250 and then with 500 mM imidazole subsequent to dialysis against NaCl (50 mM), HEPES (20 mM), glycerol (10%) and 1 µg/mL each of aprotinin, leupeptin and pepstatin for 120 min. The purification was performed either at 4 °C or on ice. To record

autophosphorylation level, EGFR kinase assay was carried out on the basis of DELFIA/Time-Resolved Fluorometry. The compounds were first dissolved in DMSO absolute, subsequent to dilution to appropriate concentration using HEPES (25 mM) at pH 7.4. Each compound (10 µL) was incubated with recombinant enzyme (10 µL, 5 ng for EGFR, 1:80 dilution in 100 mM HEPES) for 10 min at 25°C, subsequent to the addition of 5X buffer (10 µL, containing 2 mM MnCl2, 100 µM Na3VO4, 20 mM HEPES and 1 mM DTT) and ATP-MgCl2 (20 µL, containing 0.1 mM ATP and 50 mM MgCl2) and incubation for 1h. The negative and positive controls were included in each plate by the incubation of enzyme either with or without ATP-MgCl2. The liquid was removed after incubation, and the plates were washed thrice using a wash buffer. The Europium-tagged antiphosphotyrosine antibody (clone PT66) (PerkinElmer, 10 ng/well in a 20 µL final volume, resulting in a final concentration of around 0.5 nM) (75 µL, 400 ng) was added to each well followed by incubation of 1h and then washing of the plates using buffer. The enhancement solution was added to each well and the signal was recorded at excitation and emission wavelengths of 340 at 615 nm. The autophosphorylation percentage inhibition by compounds was calculated using the following equation:

$$100\% - [(negative\ control)/(positive\ control) - (negative\ control)]$$

Using the curves of percentage inhibition of eight concentrations of each compound, IC50 was calculated. The majority of signals detected by antiphosphotyrosine antibody were from EGFR because the enzyme preparation contained low impurities.

**4.2.4. HER-2 inhibitory assay**

The ADP-Glo™ Kinase Assay (Promega Corporation) was used for kinase activity detection, related kinases information: HER-2 (ab60866, Abcam), with a concentration

of 100 ng/mL. Target compounds were dissolved in DMSO to obtain drug solutions with different concentrations, and the compound concentrations in the final reaction system (100 μL) were 1 nM, 20 nM, 40 nM, 80 nM, and 100 nM, respectively. 10μL of the solution containing compounds was transferred to a 96-well plate, then added 40 μL of 1 × kinase buffer (50 mM HEPES, pH 7.5) to each well, and mixed the mixture in the 96-well plate on a shaker for 10 min. Next, added 25 μL of ADP-Glo™ reagent to the above wells, mixed and incubated for another 40 min. Distributed 10 μL kinase detection reagent to the reaction and incubated for 30 min. Finally, the full-wavelength microplate reader was used to record the OD value.

**4.2.5. VEGFR-2 inhibitory assay**

Prepare all the solutions using autoclaved, deionized water and analytical grade reagents. Prepare and store all the reagents at room temperature (unless indicated otherwise). EDTA solution (0.5 M, pH 8.0): add 95 mL ultra-pure water to 14.612 g EDTA, adjust the pH to 8.0 using NaOH solution, add 5 mL ultra-pure water; 1×Kinase Assay Buffer: add 25 mL HEPES solution (1 M), 190.175 mg EGTA, 5 mL MgCl2 solution (1 M), 1 mL DTT, 50 μL tween-20, 450 mL ultrapure water, adjust pH to 7.5 and constant volume to 500 mL using ultrapure water; 4×Stop solution (40 mM): Mix 0.8 mL of the foregoing EDTA solution, 1 mL 10×Detection Buffer and 8.2 mL ultra-pure water; 1×Detection Buffer: Mix 1 mL 10×Detection Buffer with 9 mL water; 4×VEGFR Kinase solution (1.74 nM, 521 times diluted, stored on ice): 1.2 μL VEGFR mother liquor (0.909 μM) was added to 624 μL 1×Kinase Assay Buffer and mixed; 4×ULight™-labeled JAK1 (substrate) (200 nM, 25 times diluted): 24 μL ULight™-labeled JAK1 (mother liquor concentration 5 μM) was added to 576 μL 1×Kinase Assay Buffer and mixed; 4×ATP Solution (40 μM, 250 times diluted): add 3 μL ATP solution (10 mM) to 747 μL 1×Kinase Assay Buffer and mixed; 4×Detection Mix (8 nM, 390.6

times diluted): 3 μL Europium- antiphospho-tyrosine antibody (PT66) (3.125 μM) was added to 1169 μL1×Detection Buffer and mixed; 2×substrate/ATP Mix: 560 μL foregoing 4×ULight™-labeled JAK1 and 560 μL 4×ATP solution and mixed (prepared before use). The assays used an ULight-labeled peptide substrate and a Europium-W1024-labeled anti-phosphotyrosine antibody. The VEGFR-2 was purchased from Carna Biosciences, Inc. (New York, USA). The 384-well plates were obtained from PerkinElmer. Compounds were dissolved in DMSO and diluted to 11 concentrations at a tripling rate from 2.500 μM to 0.042 nM and added 2.5 μL to 384-well plates. 5 μL 2×VEGFR-2 kinase solution (0.5 nM) was added to 384-well plates homogeneous mixing and pre-reaction at room temperature for 30 min. Next, 2.5 μL 4×Ultra ULightTM-JAK-1(Tyr1023) Peptide (200 nM)/ATP (40 μM) was added to the corresponding wells of a 384-well plate. Negative control: 2.5 μL/well 4×substrate/ATP mixture and 7.5 μL 1×kinase assay buffer in 384-well plate well. Positive control: 2.5 μL/well 4×substrate/ATP mixture, 2.5 μL/well 1×kinase assay buffer with 16% DMSO, 5 μL/well 2×VEGFR-2 kinase solution was added to the 384-well plate, The final concentration of DMSO in the mixing system was 4%. After incubation at room temperature and dark for 60 min, 5 μL 4×stop solution was added to corresponding wells to react for 5 min and then 5 μL 4×detection mix was added to the corresponding wells of a 384-well plate. The mixture was centrifugally mixed and stayed for 60 min at room temperature for color development. The plate was read using an Envision plate reader. The inhibition rate (%) = (positive well reading-compound well reading)/(positive well reading-negative well reading)×100. The corresponding $IC_{50}$ values were calculated using GraphPad Prism 5.0.

### 4.2.6 BRAF kinase assay

$V^{600E}$ mutant BRAF kinase assay was performed to investigate the activity of tested compounds against BRAF. Mouse full-length GST-tagged $BRAF^{V600E}$ (7.5 ng, Invitrogen, PV3849) was pre-incubated with drug (1 µL) and assay dilution buffer (4 µL) for 60 min at 25°C. In assay dilution buffer, a solution (5 µL) containing $MgCl_2$ (30 mM), ATP (200 µM), recombinant human full length (200 ng) and *N*-terminal His-tagged MEK1 (Invitrogen) was added to start the assay, subsequent to incubation for 25 min at 25°C. The assay was stopped using 5X protein denaturing buffer (LDS) solution (5 µL). To further denature the protein, heat (70° C) was applied for 5 min. 4-12% precast NuPage gel plates (Invitrogen) were used to carry out electrophoresis (at 200 V). 10 µL of each reaction was loaded into the precast plates and electrophoresis was allowed to proceed. After completion of electrophoresis, the front part of the precast gel plate (holding hot ATP) was cut and afterwards cast-off. The dried gel was developed using a phosphor screen. A reaction without active enzyme was used as negative control while that containing no inhibitor served as positive control. To study the effect of compounds on cell-based pERK1/2 activity in cancer cells, commercially available ELISA kits (Invitrogen) were used according to manufacturer's instructions.

### 4.2.7. Bax activation assay

Bring all reagents, except the human Bax-α Standard, to room temperature for at least 30 minutes prior to opening. The human Bax-α Standard solution should not be left at room temperature for more than 10 minutes. All standards, controls and samples should be run in duplicate. Refer to the Assay Layout Sheet to determine the number of wells to be used and put any remaining wells with the desiccant back into the pouch and seal the Ziploc. Store unused wells at 4 °C. Pipet 100 µL of Assay Buffer into the S0 (0 pg/mL standard) wells. Pipet 100 µL of Standards #1 through #6 into the appropriate

wells. Pipet 100 µL of the Samples into the appropriate wells. Tap the plate gently to mix the contents. Seal the plate and incubate at room temperature on a plate shaker for 1 hour at ~500 rpm. Empty the contents of the wells and wash by adding 400 µL of wash solution to every well. Repeat the wash 4 more times for a total of 5 washes. After the final wash, empty or aspirate the wells and firmly tap the plate on a lint free paper towel to remove any remaining wash buffer. Pipet 100 µL of yellow Antibody (**E63,** Abcam ab32503, diluted 1:100 with buffer solution) into each well, except the Blank. Seal the plate and incubate at room temperature on a plate shaker for 1 hour at ~500 rpm. Empty the contents of the wells and wash by adding 400 µL of wash solution to every well. Repeat the wash 4 more times for a total of 5 washes. After the final wash, empty or aspirate the wells and firmly tap the plate on a lint free paper towel to remove any remaining wash buffer. Add 100 µL of blue Conjugate to each well, except the Blank. Seal the plate and incubate at room temperature on a plate shaker for 30 minutes at ~500 rpm. Empty the contents of the wells and wash by adding 400 µL of wash solution to every well. Repeat the wash 4 more times for a total of 5 washes. After the final wash, empty or aspirate the wells and firmly tap the plate on a lint free paper towel to remove any remaining wash buffer. Pipet 100 µL of Substrate Solution into each well. Incubate for 30 minutes at room temperature on a plate shaker at ~500 rpm. Pipet 100 µL Stop Solution to each well. Blank the plate reader against the Blank wells, read the optical density at 450 nm. Calculate the average net Optical Density (OD) bound for each standard and sample by subtracting the average Blank OD from the average OD for each standard and sample. Using linear graph paper, plot the Average Net OD for each standard versus Bax concentration in each standard. Approximate a straight line through the points. The concentration of Bax in the unknowns can be determined by interpolation.

**4.2.8. Bcl-2 inhibition assay**

Mix all the reagents thoroughly without foaming before use. Wash the microwells twice with approximately 300 µL Wash Buffer per well with thorough aspiration of microwell contents between washes. Take caution not to scratch the surface of the microwells. After the last wash, empty the wells and tap microwell strips on absorbent pad or paper towel to remove excess Wash Buffer. Use the microwell strips immediately after washing or place upside down on a wet absorbent paper for not longer than 15 minutes. Do not allow wells to dry. Add 100 µL of Sample Diluent in duplicate to all standard wells and to the blank wells. Prepare standard (1:2 dilution) in duplicate ranging from 32 ng/mL to 0.5 ng/mL. Add 100 µL of Sample Diluent, in duplicate, to the blank wells. Add 80 µL of Sample Diluent, in duplicate, to the sample wells. Add 20 µL of each Sample, in duplicate, to the designated wells. Add 50 µL of diluted biotin-conjugate to all wells, including the blank wells. Cover with a plate cover and incubate at room temperature, on a microplate shaker at 100 rpm if available, for 2 hours. Remove plate cover and empty the wells. Wash microwell strips 3 times as described in step 2. Add 100 µL of diluted Streptavidin-HRP to all wells, including the blank wells. Cover with a plate cover and incubate at room temperature, on a microplate shaker at 100 rpm if available, for 1 hour. Remove the plate cover and empty the wells. Wash microwell strips 3 times as described in step 2. Proceed to the next step. Pipette 100 µl of mixed TMB Substrate Solution to all wells, including the blanks. Incubate the microwell strips at room temperature (18° to 25°C) for about 15 minutes, if available on a rotator set at 100 rpm. Avoid direct exposure to intense light. The point, at which the substrate reaction is stopped, is often determined by the ELISA reader. Many ELISA readers record absorbance only up to 2.0 O.D. Therefore, the color development within individual microwells must be watched by the person running the assay and the

substrate reaction stopped before positive wells are no longer properly detectable. Stop the enzyme reaction by quickly pipetting 100 μL of Stop Solution into each well, including the blank wells. It is important that the Stop Solution is spread quickly and uniformly throughout the microwells to completely inactivate the enzyme. Results must be read immediately after the Stop Solution is added or within one hour if the microwell strips are stored at 2 - 8°C in the dark. Read the absorbance of each microwell on a spectrophotometer using 450 nm as the primary wavelength.

**4.3. Molecular Docking**

The crystal structures of EGFR (PDB code: 1M17), VEGFR-2 (PDB ID: 3U6J), and HER2 (PDB code: 3RCD) were downloaded from the Protein Data Bank. Structures of compounds **10k**, **10e** and **10h** were drawn and optimized using Marvin Sketch and Avogadro molecular editors. The proteins were prepared using Autodock tools where the co-crystallized ligands and water molecules were removed then kollman charges and polar hydrogens were added. The grid dimensions for EGFR and HER-2 were set to 80x80x80. Autodock vina was used for molecular docking and the best docking poses were visualized using Discovery Studio Visualizer.

**4.4. ADME prediction**

The physicochemical and pharmacokinetic parameters of **10k** and **10e** were predicted using the SwissADME tool (http://www.swissadme.ch/index.php). Lipophilicity was estimated through five independent models: WLOGP, XLOGP3, MLOGP, iLogP, and SILICOS-IT. The arithmetic mean of these models was calculated to provide a consensus log Po/w value. The bioavailability radar illustrates six different physicochemical properties: size, polarity, lipophilicity, solubility, flexibility, and saturation.

**Table S1.** Physicochemical Properties of compounds **10k** and **10e**

| **Physicochemical Properties** | | |
|---|---|---|
| **Property** | **Compound 10k** | **Compound 10e** |
| **Formula** | $C_{23}H_{20}N_4O_2S_2$ | $C_{23}H_{20}N_4O_2S_2$ |
| **Molecular weight** | 448.56 g/mol | 448.56 g/mol |
| **Num. heavy atoms** | 31 | 31 |
| **Num. arom. heavy atoms** | 22 | 22 |
| **Fraction Csp3** | 0.13 | 0.13 |
| **Num. rotatable bonds** | 8 | 8 |
| **Num. H-bond acceptors** | 5 | 5 |
| **Num. H-bond donors** | 0 | 0 |
| **Molar Refractivity** | 124.50 | 124.50 |
| **TPSA** | 123.44 $Å^2$ | 123.44 $Å^2$ |

**Table S2.** Lipophilicity Metrics of compound compounds **10k** and **10e**

| **Lipophilicity** | | |
|---|---|---|
| **Property** | **Compound 10k** | **Compound 10e** |
| **Log $P_{o/w}$ (iLOGP)** | 4.24 | 4.15 |
| **Log $P_{o/w}$ (XLOGP3)** | 5.23 | 5.23 |
| **Log $P_{o/w}$ (WLOGP)** | 4.97 | 4.97 |
| **Log $P_{o/w}$ (MLOGP)** | 2.66 | 2.66 |
| **Log $P_{o/w}$ (SILICOS-IT)** | 5.60 | 5.60 |
| **Consensus Log $P_{o/w}$** | 4.54 | 4.52 |

**Table S3.** Water Solubility Data for compound compounds **10k** and **10e**

| **Water Solubility** | | |
|---|---|---|
| **Property** | **Compound 10k** | **Compound 10e** |
| **Log *S* (ESOL)** | -5.91 | -5.91 |
| **Solubility** | 5.48e-04 mg/ml ; 1.22e-06 mol/l | 5.48e-04 mg/ml ; 1.22e-06 mol/l |
| **Class** | Moderately soluble | Moderately soluble |
| **Log *S* (Ali)** | -7.57 | -7.57 |
| **Solubility** | 1.21e-05 mg/ml ; 2.69e-08 mol/l | 1.21e-05 mg/ml ; 2.69e-08 mol/l |
| **Class** | Poorly soluble | Poorly soluble |
| **Log *S* (SILICOS-IT)** | -7.43 | -7.43 |
| **Solubility** | 1.66e-05 mg/ml ; 3.71e-08 mol/l | 1.66e-05 mg/ml ; 3.71e-08 mol/l |
| **Class** | Poorly soluble | Poorly soluble |

**Table S4.** Pharmacokinetics Profile of compound compounds **10k** and **10e**

| Pharmacokinetics | | |
|---|---|---|
| **Property** | **Compound 10k** | **Compound 10e** |
| **GI absorption** | Low | Low |
| **BBB permeant** | No | No |
| **P-gp substrate** | No | No |
| **CYP1A2 inhibitor** | No | No |
| **CYP2C19 inhibitor** | Yes | Yes |
| **CYP2C9 inhibitor** | Yes | Yes |
| **CYP2D6 inhibitor** | No | No |
| **CYP3A4 inhibitor** | Yes | Yes |
| **Log $K_p$ (skin permeation)** | -5.32 cm/s | -5.32 cm/s |

**Table S5.** Drug-likeness Evaluation of compounds **10k** and **10e**

| Drug-likeness | | |
|---|---|---|
| **Property** | **Compound 10k** | **Compound 10e** |
| **Lipinski** | Yes; 0 violation | Yes; 0 violation |
| **Ghose** | Yes | Yes |
| **Veber** | Yes | Yes |
| **Egan** | Yes | Yes |
| **Muegge** | No; 1 violation: XLOGP3>5 | No; 1 violation: XLOGP3>5 |
| **Bioavailability Score** | 0.55 | 0.55 |

**Table S6.** Medicinal Chemistry Analysis of compounds **10k** and **10e**

| Medicinal Chemistry | | |
|---|---|---|
| **Property** | **Compound 10k** | **Compound 10e** |
| **PAINS** | 0 alert | 0 alert |
| **Brenk** | 1 alert: michael_acceptor_1 | 1 alert: michael_acceptor_1 |
| **Lead-likeness** | No; 3 violations: MW>350, Rotors>7, XLOGP3>3.5 | No; 3 violations: MW>350, Rotors>7, XLOGP3>3.5 |
| **Synthetic accessibility** | 3.95 | 3.94 |

### 4.5. Statistical analysis

Computerized Prism 5 program was used to statistically analyzed data using one-way ANOVA test followed by Tukey's as post ANOVA for multiple comparison at $P \leq .05$. Data were presented as mean ± SEM.

| Conc. (nM) | A-549 | MCF-7 | Panc-1 | HT-29 |
|---|---|---|---|---|
| | | K3 | | |
| 100 | 16.1 | 21.7 | 19.6 | 28.8 |
| 50 | 29.9 | 36.7 | 38.9 | 42.1 |
| 25 | 42.2 | 51.6 | 50.1 | 54.6 |
| 12.5 | 51.3 | 68.1 | 54.8 | 67.7 |
| 6.25 | 73.1 | 83.0 | 71.6 | 87.9 |
| 3.125 | 92.6 | 96.3 | 93.3 | 99.1 |
| 1.56 | 100 | 100 | 100 | 100 |
| | | K10 | | |
| 100 | 22.1 | 35.2 | 29.8 | 40.1 |
| 50 | 36.3 | 48.1 | 41.9 | 59.0 |
| 25 | 51.1 | 57.3 | 62.2 | 71.7 |
| 12.5 | 65.8 | 70.9 | 71.5 | 82.9 |
| 6.25 | 81.2 | 84.8 | 86.7 | 99.9 |
| 3.125 | 97.3 | 98.1 | 99.9 | 100 |
| 1.56 | 100 | 100 | 100 | 100 |

| Conc. (nM) | A-549 | MCF-7 | Panc-1 | HT-29 |
|---|---|---|---|---|
| | | P11 | | |
| 100 | 37.4 | 32.5 | 29.3 | 34.6 |
| 50 | 53.1 | 45.2 | 42.3 | 49.2 |
| 25 | 62.5 | 59.1 | 52.1 | 61.3 |
| 12.5 | 74.3 | 70.3 | 70.4 | 70.9 |
| 6.25 | 96.6 | 90.5 | 81.9 | 89.4 |
| 3.125 | 100 | 100 | 100 | 100 |
| 1.56 | 100 | 100 | 100 | 100 |
| | | P9 | | |
| 100 | 53.7 | 45.8 | 47.5 | 51.3 |
| 50 | 78.4 | 62.4 | 52.4 | 63.3 |
| 25 | 83.5 | 80.9 | 84.6 | 77.4 |
| 12.5 | 99.3 | 87.5 | 97.4 | 93.7 |
| 6.25 | 100 | 98.2 | 100 | 100 |
| 3.125 | 100 | 100 | 100 | 100 |
| 1.56 | 100 | 100 | 100 | 100 |
| | | P4 | | |

| | | | | |
|---|---|---|---|---|
| 100 | 17.8 | 17.2 | 14.5 | 24.3 |
| 50 | 25.2 | 21.9 | 20.2 | 33.7 |
| 25 | 32.7 | 30.3 | 27.6 | 41.6 |
| 12.5 | 34.5 | 39.7 | 36.7 | 48.2 |
| 6.25 | 61.6 | 55.1 | 47.3 | 61.1 |
| 3.125 | 76.5 | 71.6 | 57.1 | 87.4 |
| 1.56 | 99.1 | 83.4 | 71.2 | 99.3 |
| K8 | | | | |
| 100 | 34.5 | 32.2 | 31.6 | 34.2 |
| 50 | 49.6 | 45.8 | 42.3 | 41.9 |
| 25 | 65.3 | 59.4 | 50.2 | 54.3 |
| 12.5 | 77.1 | 70.5 | 66.7 | 66.4 |
| 6.25 | 93.8 | 92.7 | 80.4 | 79.7 |
| 3.125 | 100 | 100 | 98.2 | 97.8 |
| 1.56 | 100 | 100 | 100 | 100 |
| P2 | | | | |
| 100 | 33.6 | 29.2 | 24.3 | 34.2 |
| 50 | 45.7 | 37.4 | 27.8 | 42.3 |
| 25 | 60.5 | 43.5 | 37.4 | 56.7 |
| 12.5 | 73.4 | 51.9 | 48.7 | 68.2 |
| 6.25 | 81.1 | 78.3 | 73.2 | 80.5 |
| 3.125 | 97.2 | 94.7 | 89.5 | 98.8 |
| 1.56 | 100 | 100 | 96.1 | 100 |
| P7 | | | | |
| 100 | 28.7 | 25.2 | 27.4 | 29.3 |
| 50 | 36.9 | 30.4 | 34.3 | 37.2 |
| 25 | 45.4 | 46.7 | 41.2 | 44.5 |
| 12.5 | 51.5 | 54.3 | 54.1 | 52.6 |
| 6.25 | 80.1 | 72.8 | 69.1 | 72.2 |
| 3.125 | 89.8 | 83.3 | 85.5 | 90.4 |
| 1.56 | 99.2 | 97.3 | 97.6 | 100 |
| P8 | | | | |
| 100 | 45.4 | 49.2 | 42.2 | 47.3 |
| 50 | 58.6 | 61.1 | 58.4 | 66.2 |
| 25 | 72.3 | 75.3 | 69.4 | 76.4 |
| 12.5 | 84.1 | 92.1 | 85.2 | 92.5 |
| 6.25 | 96.5 | 100 | 99.3 | 100 |
| 3.125 | 100 | 100 | 100 | 100 |
| 1.56 | 100 | 100 | 100 | 100 |
| P3 | | | | |
| 100 | 25.7 | 27.1 | 25.7 | 27.8 |
| 50 | 42.6 | 43.3 | 38.4 | 42.7 |
| 25 | 53.4 | 57.2 | 50.5 | 55.5 |

| | | | | |
|---|---|---|---|---|
| 12.5 | 66.5 | 60.5 | 58.9 | 68.3 |
| 6.25 | 84.1 | 85.6 | 86.2 | 87.9 |
| 3.125 | 100 | 100 | 93.8 | 100 |
| 1.56 | 100 | 100 | 100 | 100 |
| | | P10 | | |
| 100 | 30.2 | 33.6 | 31.6 | 36.5 |
| 50 | 49.4 | 47.1 | 46.7 | 45.8 |
| 25 | 62.8 | 60.2 | 57.1 | 62.4 |
| 12.5 | 79.3 | 73.9 | 77.4 | 73.5 |
| 6.25 | 89.5 | 86.5 | 88.5 | 89.7 |
| 3.125 | 97.6 | 94.8 | 95.7 | 100 |
| 1.56 | 100 | 100 | 100 | 100 |
| | | P13 | | |
| 100 | 33.4 | 36.3 | 33.5 | 38.3 |
| 50 | 57.2 | 55.5 | 55.6 | 46.4 |
| 25 | 72.3 | 69.4 | 63.8 | 61.3 |
| 12.5 | 80.9 | 85.1 | 85.1 | 79.2 |
| 6.25 | 87.4 | 89.5 | 85.3 | 90.4 |
| 3.125 | 100 | 100 | 100 | 100 |
| 1.56 | 100 | 100 | 100 | 100 |
